# Highly Stable, Readily Reducible, Fluorescent, Trifluoromethylated 9‐Borafluorenes

**DOI:** 10.1002/chem.201905559

**Published:** 2020-09-21

**Authors:** Florian Rauch, Sonja Fuchs, Alexandra Friedrich, Daniel Sieh, Ivo Krummenacher, Holger Braunschweig, Maik Finze, Todd B. Marder

**Affiliations:** ^1^ Institute for Inorganic Chemistry and Institute for Sustainable Chemistry & Catalysis with Boron (ICB) Julius-Maximilians-Universität Würzburg Am Hubland 97074 Würzburg Germany

**Keywords:** borafluorenes, boron, EPR spectroscopy, fluorescence, heterocycles

## Abstract

Three different perfluoroalkylated borafluorenes (^**F**^ **Bf**) were prepared and their electronic and photophysical properties were investigated. The systems have four trifluoromethyl moieties on the borafluorene moiety as well as two trifluoromethyl groups at the *ortho* positions of their *exo*‐aryl moieties. They differ with regard to the *para* substituents on their *exo*‐aryl moieties, being a proton (^**F**^ **Xyl^F^Bf**, ^F^Xyl: 2,6‐bis(trifluoromethyl)phenyl), a trifluoromethyl group (^**F**^ **Mes^F^Bf**, ^F^Mes: 2,4,6‐tris(trifluoromethyl)phenyl) or a dimethylamino group (***p*** **‐NMe_2_‐^F^Xyl^F^Bf**, *p*‐NMe_2_‐^F^Xyl: 4‐(dimethylamino)‐2,6‐bis(trifluoromethyl)phenyl), respectively. All derivatives exhibit extraordinarily low reduction potentials, comparable to those of perylenediimides. The most electron‐deficient derivative ^**F**^ **Mes^F^Bf** was also chemically reduced and its radical anion isolated and characterized. Furthermore, all compounds exhibit very long fluorescent lifetimes of about 250 ns up to 1.6 μs; however, the underlying mechanisms responsible for this differ. The donor‐substituted derivative ***p*** **‐NMe_2_‐^F^Xyl^F^Bf** exhibits thermally activated delayed fluorescence (TADF) from a charge‐transfer (CT) state, whereas the ^**F**^ **Mes^F^Bf** and ^**F**^ **Xyl^F^Bf** borafluorenes exhibit only weakly allowed locally excited (LE) transitions due to their symmetry and low transition‐dipole moments.

## Graphical abstract

**Having your cake and eating it too**: Functionalization of 9‐borafluorenes with trifluoromethyl groups makes them exceptionally easy to reduce while maintaining excellent stability towards hydrolysis. The systems also exhibit long‐lived fluorescence or thermally activated delayed fluorescence (TADF) depending on the *exo*‐aryl *para*‐substituent.

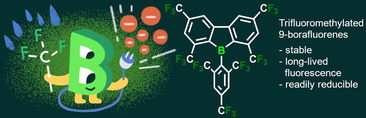

## Introduction

**Figure 1:**
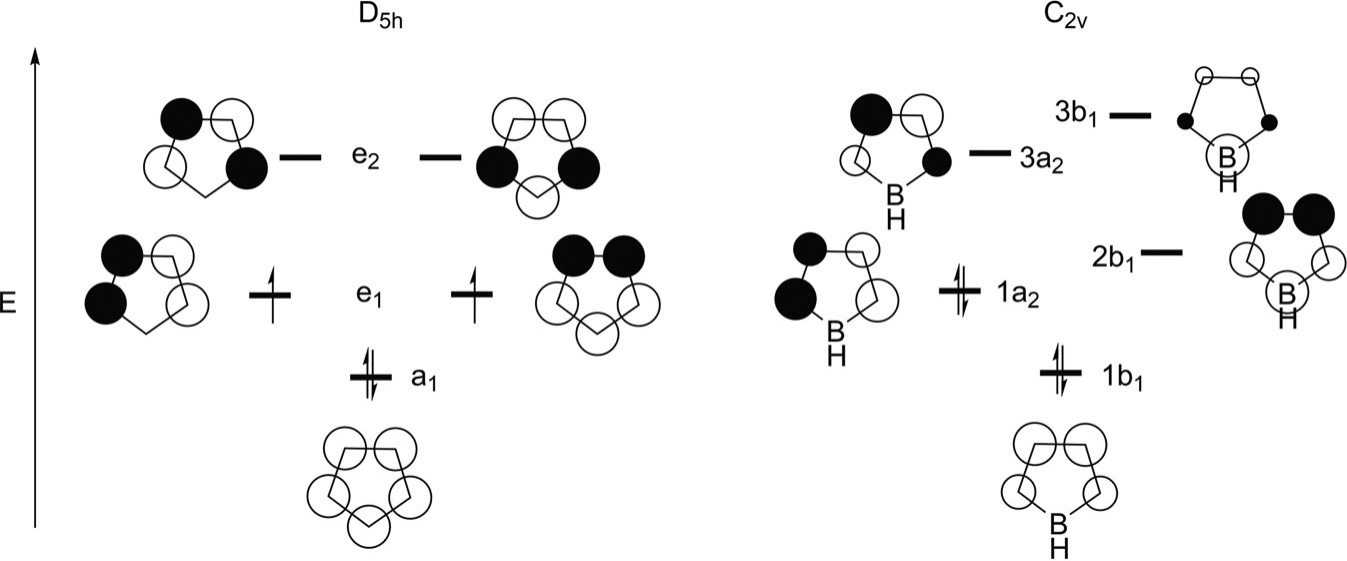
Frontier orbitals of the cyclopentadienyl cation (left) and borole (right).

Boron‐containing organic π‐systems, especially triarylboranes^1^, ^2^, ^3^, ^4^, ^5^, ^6^, ^7^, ^8^, ^9^, ^10^, ^11^, ^12^, ^13^ and, more recently, boron‐containing polyaromatics are of much current interest.^3^, ^10^, ^14^, ^15^, ^16^, ^17^, ^18^, ^19^, ^20^ Three‐coordinate boron is isoelectronic with a carbonium ion, having an unoccupied p‐orbital, making it inherently electron deficient and Lewis acidic. Thus, three‐coordinate boranes can be employed as π‐acceptors, single‐electron or electron‐pair acceptors. Such boranes have been used in linear^21^, ^22^, ^23^, ^24^, ^25^, ^26^, ^27^, ^28^, ^29^, ^30^, ^31^, ^32^, ^33^, ^34^, ^35^, ^36^, ^37^, ^38^, ^39^, ^40^ and non‐linear^41^, ^42^, ^43^, ^44^, ^45^, ^46^, ^47^, ^48^, ^49^, ^50^, ^51^, ^52^, ^53^ optical materials, anion sensors,^8^, ^54^, ^55^, ^56^ frustrated Lewis pairs (FLPs),^57^, ^58^, ^59^, ^60^, ^61^, ^62^, ^63^ as well as in organic light‐emitting diodes (OLEDs).^64^, ^65^, ^66^ There are numerous examples, both aromatic and antiaromatic, of boron‐containing conjugated cyclic π‐systems.^16^, ^20^ The subclass of boroles is of special interest.^3^, ^18^, ^67^, ^68^, ^69^, ^70^, ^71^, ^72^, ^73^, ^74^, ^75^, ^76^, ^77^, ^78^, ^79^, ^80^, ^81^, ^82^ They are isoelectronic with the antiaromatic cyclopentadiene cation, having 4 π electrons; however, the introduction of the boron vertex lowers the symmetry (Figure 1). This leads to a singlet ground state with a small HOMO–LUMO gap, resulting in the intense color of boroles, even though the molar extinction coefficient (*ϵ*) is quite small for their S_1_←S_0_ transitions.

**Figure 2:**
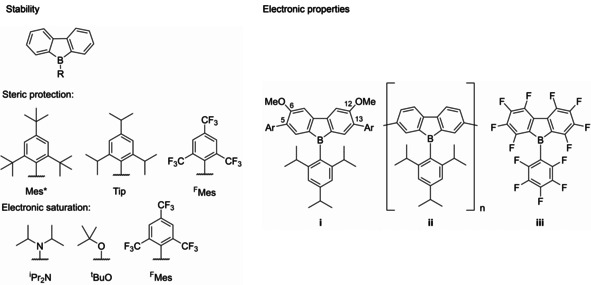
Selected examples of borafluorenes. A variety of *exo*‐aryl substituents and the nature of their respective influence on the stability of the borafluorene (left).^87^, ^88^ It is important to note, that *ortho*‐trifluoromethyl substituted aryls provide both steric as well as electronic stabilization. Examples of the functionalization of the borafluorene backbone to tune the electronic properties (right).^86^, ^95^, ^96^, ^97^

Because of that, they are highly reactive towards nucleophiles and thereby unsuitable for many applications. Steric shielding, which works well for triarylboranes,^33^, ^83^, ^84^ only stabilizes boroles to a certain degree.^79^, ^85^ Through benzannulation, the stability of boroles can be greatly increased,^86^, ^87^, ^88^, ^89^ but the anti‐aromatic character is significantly decreased due to delocalization of π‐electron density over the biphenylene backbone. This leads to a stabilization of the HOMO as well as a destabilization of the LUMO resulting in a larger HOMO–LUMO gap and loss of the characteristic strong color of boroles. This also results in a lower Lewis acidity and, subsequently, significantly more stable systems.^16^, ^77^ Detailed studies by Martin and co‐workers demonstrate, however, that sterically less hindered derivatives, in particular, retain characteristic borole reactivity.^90^, ^91^, ^92^, ^93^, ^94^ Compared with their triarylborane derivatives, borafluorenes are usually more Lewis acidic and exhibit more positive reduction potentials.^95^ The stability of these systems can be further improved by sterically shielding^87^, ^88^ or electronically saturating the boron center,^86^ through direct n‐to‐p conjugation or indirect F−B interaction (Figure 2).

Yamaguchi and co‐workers reported air and moisture‐stable borafluorene derivatives employing either Tip (2,4,6‐tris(triisopropyl)phenyl) or the even bulkier Mes* (2,4,6‐tris(*tert*‐butyl)phenyl) substituents.^86^, ^87^ It was found that the Tip derivatives could be used as turn‐on type fluoride sensors, whereas the Mes* compounds showed no reaction with fluoride. Recently, Rupar and co‐workers have studied these effects in detail.^88^ They found that although Tip‐substituted derivatives still decompose slowly (<10 % decomp. over 24 h) in wet solvents, the corresponding ^F^Mes derivatives exhibit higher stability (5 % decomp. over 24 h). Derivatives containing π‐bonding moieties were found to be much more sensitive towards moisture (*i*PrN_2_: 50 % decomp. over 1 h; *t*BuO: 10 % decomp. over 1 h). *ortho*‐Trifluoromethyl‐substituted aryls exhibit a strong stabilizing effect on boranes.^33^, ^79^, ^98^, ^99^, ^100^, ^101^, ^102^ In addition to the steric effect, a direct interaction of the lone pairs of the fluorine atoms with the empty p orbital of the boron center is observed. This is supported by B−F distances which are much shorter than the sum of their van der Waals radii (3.39 Å)^103^ in crystal structures. The electronic properties of borafluorenes can be easily tuned to fit different applications by the introduction of different substitution patterns on the biphenyl backbone (Figure 2, right). The introduction of methoxy groups at the 6 and 12 positions leads to a small hypsochromic shift of both the absorption and emission wavelength,^86^ whereas elongation of the π‐system with electron‐rich conjugated systems attached at the 5 and 13 positions leads to a bathochromic shift of both the absorption and emission wavelength.^87^ The photophysical properties of borafluorenes can also be modified by coordination of Lewis bases. Both Yamaguchi and co‐workers and Rivard and co‐workers observed turn‐on fluorescence upon adduct formation.^86^, ^104^ Wilson and Gillard and co‐workers observed turn‐off fluorescence of a borafluorenium cation upon coordination of a Lewis base at low temperature, resulting in thermochromism.^105^ Piers and co‐workers investigated the properties of a highly electron‐deficient perfluorinated borafluorene **iii**.^95^, ^96^ Although they only observed a reduction corresponding to the perfluoroaryls in the cyclic voltammogram, a reaction with the relatively mild reducing agent CoCp_2_ (CoCp_2_/CoCp_2_^+^: −1.3 vs. Fc/Fc^+^) was observed, underlining the electron‐deficient nature of the compound. In competition experiments with the strong Lewis acid B(C_6_F_5_)_3_, preference towards the borafluorene derivative was observed, especially with sterically demanding Lewis bases. The (sp^2^‐C)−F bonds in this compound, however, are still reactive towards nucleophiles. In contrast, perfluoroalkyl groups are inert towards nucleophiles, provide a strong inductive electron‐withdrawing effect and have been previously employed in the synthesis of electron‐deficient triarylboranes.^102^ To the best of our knowledge, there have been no photophysical studies of borafluorenes with electron‐deficient biphenyl backbones. We envisioned that judicious incorporation of trifluoromethyl groups both in the biphenyl core and at the *exo*‐aryl moiety would provide a significant stability enhancement while retaining the low‐lying LUMO typical of non‐annulated boroles.

## Results and Discussion

### Synthesis

**Scheme 1:**
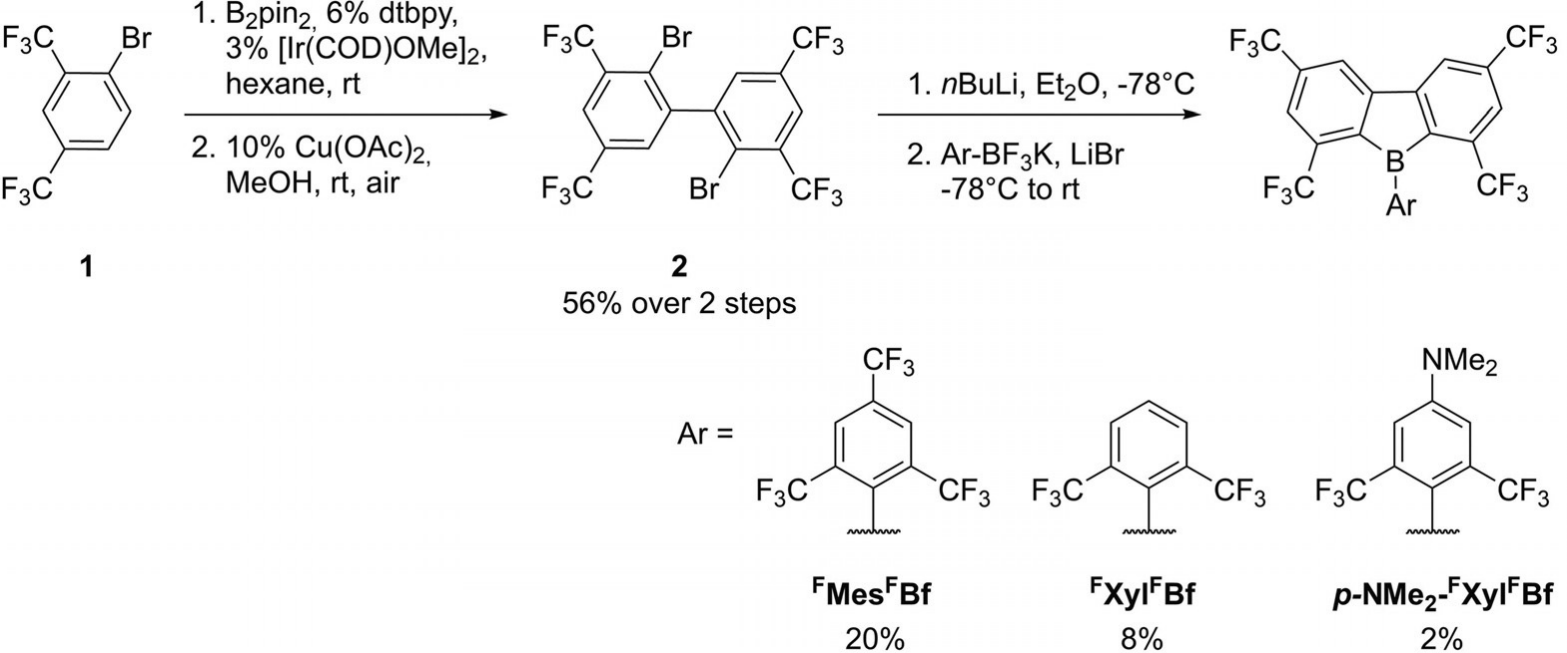
Synthesis of ^**F**^ **Mes^F^Bf**, ^**F**^ **Xyl^F^Bf** and ***p*** **‐NMe_2_‐^F^Xyl^F^Bf**.

In order to maximize the stability of the trifluoromethylated borafluorenes (^**F**^ **Bf**) we chose three different *meta*‐fluoroxylene (1,3‐bis(trifluoromethyl)benzene) derivatives as the *exo*‐aryl moieties. To examine the influence of the *exo*‐aryl and, specifically, substituents at the *para* position, we chose 2,6‐bis(trifluoromethyl)phenyl (^**F**^ **Xyl**), 2,4,6‐tris(trifluoromethyl)phenyl (^**F**^ **Mes**) and 4‐(dimethylamino)‐2,6‐bis(trifluoromethyl)phenyl (***p*** **‐NMe_2_‐^F^Xyl**) groups (Scheme 1).

Biphenyl derivative **2** was synthesized through regioselective C−H borylation of **1** 106, 107 *ortho* to the bromine and a subsequent copper‐catalyzed oxidative homocoupling. For the last step, **2** was dilithiated and subsequently reacted with the appropriate Ar‐BF_3_K salt. Attempts to synthesize the haloborafluorene with different BX_3_ (X=F, Cl, Br) sources failed. Attempts to use aryl boronates in place of the Ar‐BF_3_K salt were also unsuccessful. The use of aryltrifluoroborate salts as boron source was previously reported by our group for the synthesis of boroles with enhanced stability,^79^ and applied by others in the synthesis of boron polyaromatic hydrocarbons (PAHs)^108^, ^109^ and aryl borates.^110^, ^111^ Organic trifluoroborate salts are widely employed in cross‐coupling reactions as they are readily accessible and very stable.^112^, ^113^ During the synthesis we observed that adding LiBr greatly improves the reactivity of the Ar‐BF_3_K salts. It is possible that a cation‐exchange reaction generates the more reactive Ar‐BF_3_Li salt. The increased reactivity of the Ar‐BF_3_Li salt is due to the thermodynamically favorable LiF elimination. It is also possible that LiBr stabilizes the aryllithium species towards decomposition in ethereal solvents. This decomposition also explains the low yields of isolated material. It is important to note that the corresponding *ortho*‐trifluoromethylarylboron halides (X=Cl, Br) are not stable due to halide exchange.^114^ This might also explain why the synthesis of the haloborafluorenes was not possible. The compounds ^**F**^ **Mes^F^Bf**, ^**F**^ **Xyl^F^Bf**, and ***p*** **‐NMe_2_‐^F^Xyl^F^Bf** were obtained after purification through sublimation and recrystallization. Both ^**F**^ **Mes^F^Bf** and ^**F**^ **Xyl^F^Bf** are bright‐green solids. In contrast, ***p*** **‐NMe_2_‐^F^Xyl^F^Bf** is a red solid. All compounds exhibit ^1^H NMR and ^13^C{^1^H} NMR signals consistent with their proposed structures. The ^11^B{^1^H} NMR shifts for all three borafluorene derivatives are around 64 ppm and differ only slightly. The ^19^F{^1^H} NMR spectra display singlets and septets, the latter with a *J*_FF_ coupling constant of 3–4 Hz. (Table 1)

**Table 1:** ^**11**^B{^1^H} and ^19^F{^1^H} NMR shifts of ^**F**^ **Mes^F^Bf**, ^**F**^ **Xyl^F^Bf**, and ***p*** **‐NMe_2_‐^F^Xyl^F^Bf** recorded in C_6_D_6_.

	^**F**^ **Xyl^F^Bf**	^**F**^ **Mes^F^Bf**	***p‐*** **NMe_2_‐^F^Xyl^F^Bf**
^11^B{^1^H} NMR [ppm]	63.2	64.1	64.7
^19^F{^1^H} NMR [ppm] singlet	−63.4	−62.0; −63.5	−63.4
^19^F{^1^H} NMR [ppm] septets	−58.2 (*J*_FF_=4 Hz) −59.6 (*J*_FF_=4 Hz)	−58.4 (*J*_FF_=4 Hz) −59.6 (*J*_FF_=4 Hz)	−58.1 (*J*_FF_=4 Hz) −59.5 (*J*_FF_=4 Hz)

The singlets at about *δ*=−63.5 ppm correspond to the two freely rotating *para* CF_3_ groups on the borafluorene core. For ^**F**^ **Mes^F^Bf**, another singlet corresponding to the *para* CF_3_ group on the *exo*‐aryl is observed. The CF_3_ groups *ortho* to the boron center display a complex coupling pattern of two septets with small coupling constants (*J*_FF_=4 Hz). This can be attributed to through‐space F−F coupling as previously observed at low temperature (243 K) for (^F^Mes)_2_BAr compounds.^100^ The fact that the borafluorenes exhibit this phenomenon at room temperature is an indicator of the high rigidity of the systems.

**Figure 3:**
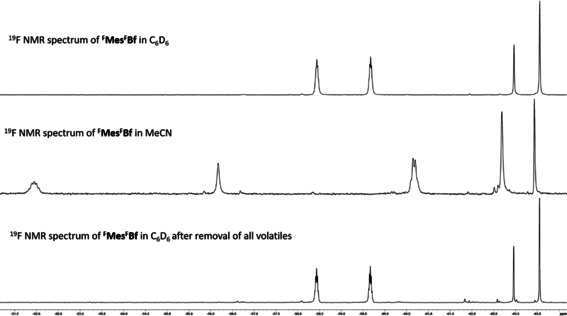
^19^F NMR spectra (188 MHz, 298 K) of ^**F**^ **Mes^F^Bf** in C_6_D_6_ (top), in CH_3_CN (middle) and in C_6_D_6_ after removal of all volatiles (bottom).

All three compounds are stable in the solid state and can be stored under ambient conditions without decomposition. In wet CDCl_3_ (1.5 equiv. H_2_O per borafluorene) at room temperature, no decomposition of either ^**F**^ **Mes^F^Bf** or ^**F**^ **Xyl^F^Bf** was observed over 4 days by NMR spectroscopy. This is surprising given that for ^**F**^ **MesBf** and **TipBf**, both less electron‐deficient compounds, decomposition rates of 5 and 10 % respectively in wet solvents over 24 h were reported.^88^ This indicates that the CF_3_‐groups *ortho* to the boron center on the borafluorene core have a stabilizing effect, likely due to steric shielding. However, ***p*** **‐NMe_2_‐^F^Xyl^F^Bf** shows very rapid decomposition when exposed to wet solvents. It is likely that the dimethylamine moiety is protonated first, thereby further increasing the electrophilicity of the boron center and decreasing its stability towards nucleophilic attack. The reaction with H_2_O leads to cleavage of one B−C bond of the borafluorene core, resulting in a BOH and CH moiety. The same reactivity towards water and other E−H bonds (E=N, O, S) was previously observed by Martin and co‐workers.^94^ Likely due to less steric hinderance in their system, a second borafluorene reacts with the decomposition product to form a B‐O‐B motif. The product of the hydrolysis of ***p*** **‐NMe_2_‐^F^Xyl^F^Bf** was isolated and studied by X‐ray diffraction (compound **D** in Figure S41, Supporting Information). All three compounds are slightly soluble in non‐polar solvents such as hexane or toluene and soluble in polar non‐coordinating solvents such as CH_2_Cl_2_ and THF. Dissolving ^**F**^ **Mes^F^Bf** in acetonitrile gives a colorless solution. Investigation of the solution using ^19^F NMR spectroscopy revealed the formation of an acetonitrile adduct, which is consistent with previous studies by Martin and co‐workers for less sterically hindered borafluorenes.^91^ The *para*‐CF_3_ groups, both on the borafluorene backbone as well as the *exo*‐aryl moiety, are influenced only weakly by the coordination of acetonitrile, because both singlets in the ^19^F NMR spectrum shift only slightly to lower field. The signals corresponding to the *ortho* CF_3_‐groups, however, change dramatically. Instead of two septets as observed for ^**F**^ **Mes^F^Bf**, one septet at −51.9 ppm (*J*_FF_=10 Hz), one broad singlet at −56.2, and a quartet at −60.7 ppm (*J*_FF_=10 Hz), are observed (Figure 3, middle).

This suggests that only one *exo*‐aryl trifluoromethyl moiety is coupling to the *ortho* trifluoromethyl groups on the borafluorene backbone. After evaporation of the acetonitrile and dissolution in C_6_D_6_, only the borafluorene was observed through ^19^F NMR spectroscopy. Thus, the formation of the adduct with acetonitrile is completely reversible.

### Crystal and molecular structures

**Figure 4:**
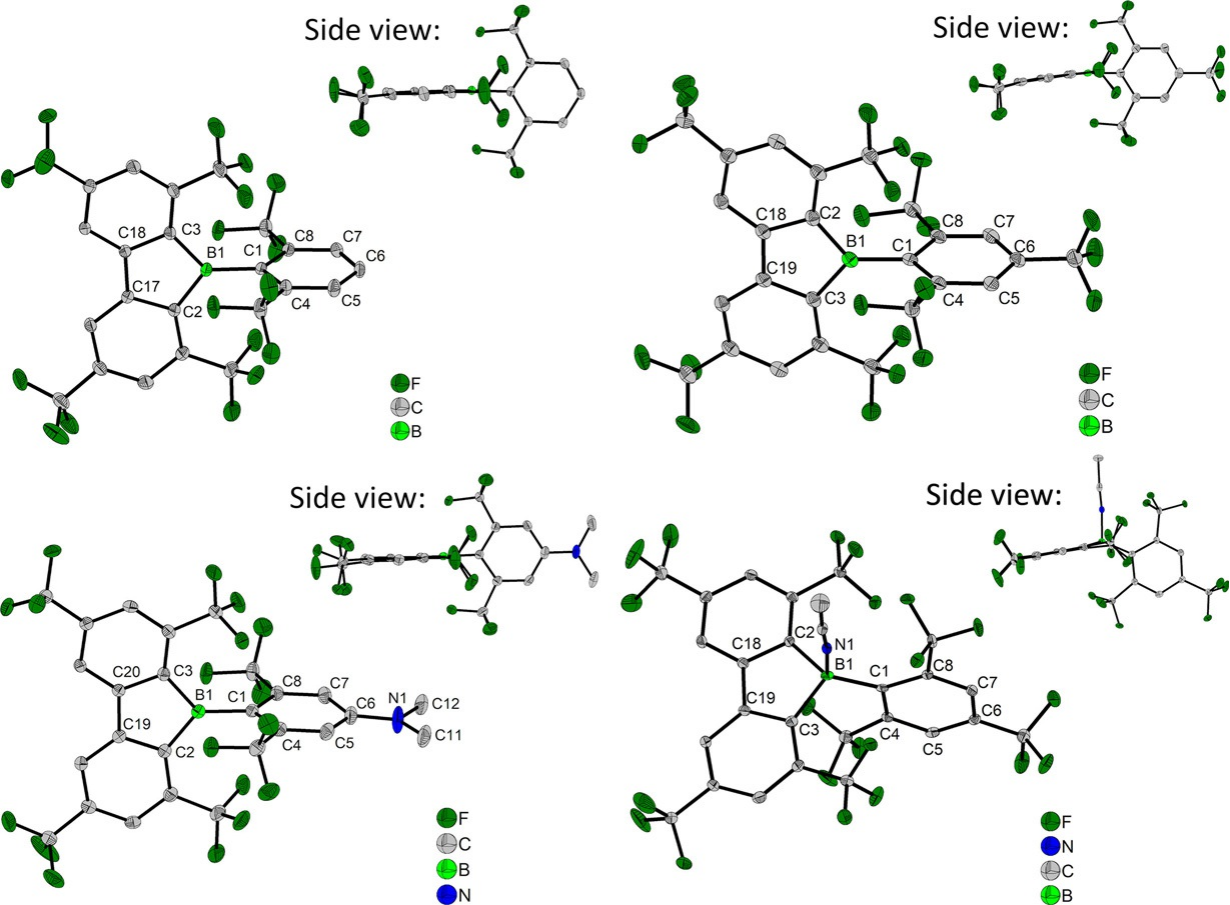
Molecular structures of ^**F**^ **Xyl^F^Bf** (top left), ^**F**^ **Mes^F^Bf** (top right), ***p*** **‐NMe_2_‐^F^Xyl^F^Bf** (bottom left) and ^**F**^ **Mes^F^Bf⋅MeCN** (bottom right) determined by single‐crystal X‐ray diffraction at 100 K. All ellipsoids are drawn at the 50 % probability level, and H atoms and solvent molecules are omitted for clarity.

Single crystals of the three borafluorenes as well as the acetonitrile adduct of ^**F**^ **Mes^F^Bf** (^**F**^ **Mes^F^Bf⋅MeCN**) suitable for X‐ray studies were obtained (Figure 4) and selected bond lengths, angles, torsion angles and short B−F contacts are listed in Table 2. The single crystals of ^**F**^ **Mes^F^Bf** and ***p*** **‐NMe_2_‐^F^Xyl^F^Bf** were obtained from a saturated hexane solution at −30 °C, that of ^**F**^ **Xyl^F^Bf** was obtained by evaporation of a saturated CH_2_Cl_2_ solution and that of ^**F**^ **Mes^F^Bf⋅MeCN** was obtained from a saturated acetonitrile solution at −30 °C.

**Table 2:** Selected bond lengths [Å] and angles [°] **in** ^**F**^ **Mes^F^Bf**, ^**F**^ **Xyl^F^Bf**, ***p‐*** **NMe_2_‐^F^Xyl^F^Bf** and ^**F**^ **Mes^F^Bf⋅MeCN**. Atom labels for the respective molecular structures are shown in Figure 4.

	^**F**^ **Mes^F^Bf**	^**F**^ **Xyl^F^Bf**	***p‐*** **NMe_2_‐^F^Xyl^F^Bf**	^**F**^ **Mes^F^Bf⋅MeCN**
B−C1 B−C2 B−C3 B−N	1.579(3) 1.581(3) 1.591(3)	1.570(2) 1.591(3) 1.591(3)	1.570(3) 1.595(3) 1.591(3)	1.652(3) 1.645(3) 1.645(3) 1.591(3)
C3−C19/C18/C20 C2−C18/C17/C19 C18/C17/C19−C19/C18/C20	1.408(3) 1.410(2) 1.481(3)	1.410(2) 1.409(2) 1.474(2)	1.409(2) 1.408(2) 1.482(2)	1.401(3) 1.410(3) 1.475(3)
∡ BC_3_−Aryl_*exo*_	89.32(7)	89.84(8)	89.27(6)	
∡ BC_12_−Aryl_*exo*_	89.43(6)	89.54(6)	88.69(5)	82.12(6)
torsion of Ar_*exo*_ out of borafluorene plane ∡ C1‐B‐C2‐C18/C17/C19	171.28(16)	176.45(16)	178.83(17)	137.6(2)
5‐ring: ∡ C2BC3	103.95(15)	103.07(13)	102.94(14)	99.74(18)
Sum ∡ CBC	359.74(16)	359.90(15)	359.99(16)	338.43(18)
sum ∡ CNC			359.84(19)	
shortest B−F contact(s)	2.392(3) 2.440(3)	2.379(2) 2.390(3)	2.366(2) 2.434(2)	2.853(3)

A comparison of the crystal structures of the three target compounds shows the following. Although all three B−C bond lengths are in a similar range for ^**F**^ **Mes^F^Bf** (1.579(3)–1.591(3) Å), the B−C1_*exo*_ distances to the ^**F**^ **Xyl** groups of ^**F**^ **Xyl^F^Bf** and ***p*** **‐NMe_2_‐^F^Xyl^F^Bf** (B−C=1.570(3) Å) are slightly shorter than the respective B−C2 and B−C3 bonds (1.591(3)–1.595(3) Å) within the borole moieties (Table 2). The boron atoms have a nearly ideal trigonal‐planar configuration with the sum of the C‐B‐C angles being 359.74(16)–359.99(16)°. In all three compounds, the C2‐B‐C3 angle (102.94(14)–103.95(15)°) within the borole moiety is much smaller than the other two C‐B‐C angles. The borole moiety shows similar bond lengths and angles in all three compounds. The angles increase from C‐B‐C to B‐C‐C (106.57(16)–107.52(15)°) and to C‐C‐C (110.98(17)–111.41(15)°). The C2−C and C3−C bond lengths (1.408(3)–1.410(2) Å) are typical for aromatic bonds, whereas the C−C bond (1.474(2)–1.482(2) Å) that is opposite to the boron atom has significant single‐bond character. The interplanar angle between the borafluorene (BC_12_) and the *exo*‐aryl substituent is close to 90° in all three compounds (88.69(5)–89.54(6)°). This is due to the large steric demand of the CF_3_ groups in the *ortho* positions of both the *exo*‐aryl moiety as well as the borafluorene core. In all three compounds, two B−F distances, each in the range of 2.366(2)–2.440(3) Å, are observed, which are significantly shorter than the sum of the van der Waals radii for boron and fluorine (3.39 Å).^103^ This was previously observed in boranes and boroles with *ortho*‐CF_3_ aryl moieties.^33^, ^79^, ^102^, ^114^, ^115^ Given that the two respective fluorine atoms are directly above and below the boron center, it is most likely that the lone pair electrons of these fluorine atoms interact with the empty p‐orbital of the boron center. The torsion angle C_*endo*_‐C2_*endo*_‐B‐C1_*exo*_ with the *endo* carbon atoms belonging to the borole moiety deviates slightly from 180° (171.28(16)–178.83(17)°). This shows that the B−C1 bond to the *exo*cyclic moiety is tilted slightly out of the borafluorene plane. The out‐of‐plane tilt increases from ***p*** **‐NMe_2_‐^F^Xyl^F^Bf** (1.17(17)°) to ^**F**^ **Xyl^F^Bf** (3.55(16)°) and ^**F**^ **Mes^F^Bf** (8.72(16)°). The magnitude of the tilt is related to the molecular packing, which is similar in all three crystal structures because the borafluorene moieties are arranged in pairs, which are related by inversion symmetry and form weak intermolecular π⋅⋅⋅π interactions between the borafluorene backbones. The strongest π⋅⋅⋅π interaction is observed in ^**F**^ **Mes^F^Bf**, which shows the smallest centroid– distance, interplanar separation, and offset shift (Table S3, Supporting Information). Hence, these pairs of molecules are closest in ^**F**^ **Mes^F^Bf**, and the exocyclic ^**F**^ **Mes** moiety is tilted out of the plane the most, away from the center of the pair in order to avoid close F⋅⋅⋅F contacts between the two molecules. In the crystal structure of the acetonitrile adduct of ^**F**^ **Mes^F^Bf**, as the hybridization at the boron is now sp^3^ rather than sp^2^, all of the B−C bonds are elongated. The C2‐B1‐C3 angle of the borole ring (99.74(18)°) is decreased compared to ^**F**^ **Mes^F^Bf** (103.95(15)°). The torsion angle of the *exo*‐aryl towards the borafluorene backbone is decreased (82.12(6)°) and the bending of the *exo*‐aryl out of the plane of the borafluorene moiety (137.6(2)°) deviates significantly from 180°. In the adduct, there is only one short B−F contact (F3−B1=2.853(3) Å) that is elongated compared to ^**F**^ **Mes^F^Bf**, but still shorter than the sum of the van der Waals radii. The B−N bond length is 1.591(3) Å, which is significantly shorter than that in the acetonitrile adduct of B(C_6_F_6_)_3_ (1.616(3) Å),^116^ but similar to that in the previously reported MeCN adduct of **PhBf** (**PhBf**⋅**MeCN**) (1.598(4) Å).^91^ The N≡C bond (1.129(3) Å) is also shorter than that in the acetonitrile adduct of B(C_6_F_6_)_3_ (1.141(2) Å) and very similar to that in **PhBf**⋅**MeCN** (1.128(4) Å).

### Electrochemistry

**Figure 5:**
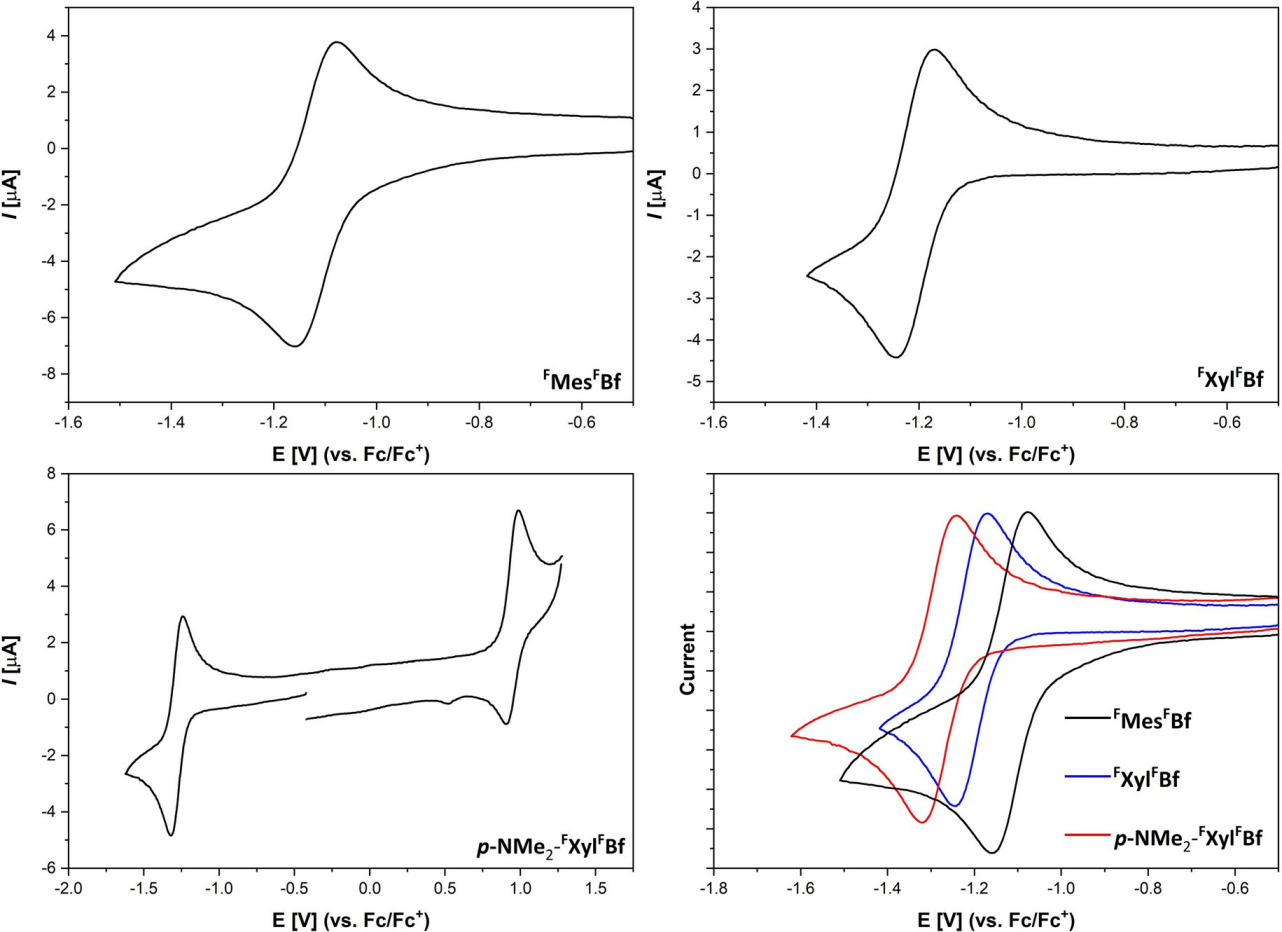
Cyclic voltammograms of the reversible redox events of ^**F**^ **Mes^F^Bf** (top left), ^**F**^ **Xyl^F^Bf** (top right) and ***p*** **‐NMe_2_‐^F^Xyl^F^Bf** (bottom left). All samples are referenced against the Fc/Fc^+^ redox couple. For better comparison, the reduction waves are plotted together (bottom right; ^**F**^ **Mes^F^Bf** (black), ^**F**^ **Xyl^F^Bf** (blue), ***p*** **‐NMe_2_‐^F^Xyl^F^Bf** (red)).

Cyclic voltammograms of the three borafluorenes were recorded in dichloromethane with [*n*Bu_4_N][PF_6_] as the electrolyte and a scan rate of 250 mV s^−1^ (Figure 5) in order to determine their reduction potentials. All measurements were referenced to the ferrocene/ferrocenium redox couple (Fc/Fc^+^). The most electron‐deficient borafluorene, ^**F**^ **Mes^F^Bf**, exhibits a reversible reduction at −1.13 V and an irreversible reduction at −2.04 V. For the slightly less electron‐deficient ^**F**^ **Xyl^F^Bf**, a reversible reduction at −1.21 V and an irreversible reduction at −2.12 V are observed. Interestingly, ***p*** **‐NMe_2_‐^F^Xyl^F^Bf** shows a reversible reduction at −1.28 V, an irreversible reduction at −2.15 V and a partially reversible oxidation at 0.95 V.

**Figure 6:**
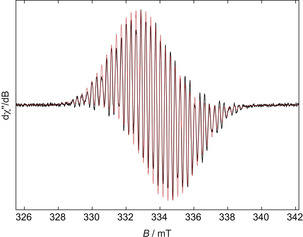
Experimental (black solid line) and simulated (red dashed line) continuous‐wave X‐band EPR spectra of [^**F**^ **Mes^F^Bf**]^⋅−^ in a THF solution at room temperature.

The three borafluorenes exhibit much higher reduction potentials (^**F**^ **Mes^F^Bf**=−1.13 V; ^**F**^ **Xyl^F^Bf**=−1.21 V and ***p*** **‐NMe_2_‐^F^Xyl^F^Bf**=−1.28 V) than any of the previously reported borafluorenes and boroles or triarylboranes (Table 3). The substitution patterns of compounds ^**F**^ **MesBC_4_Ph_4_** (*E*_1/2_=−1.52 V) and ^**F**^ **MesBf** (*E*_1/2_=−1.82 eV) allows a direct comparison of the fluorinated borafluorene backbone (^**F**^ **Bf**) with the unsubstituted borafluorene backbone (**Bf**) and the non‐annulated borole.^88^ The strong anodic shift of the ^**F**^ **Mes^F^Bf** as compared to ^**F**^ **MesBC_4_Ph_4_** and ^**F**^ **MesBf** demonstrates the strong electron‐withdrawing effect of the CF_3_ groups on the borafluorene backbone. This is likely due to the planar geometry as well as the fact that the *ortho* CF_3_‐groups of the borafluorene backbone do not display B−F interactions, and thus do not increase electron density at the boron atom. The reduction potentials of the trifluoromethylated borafluorenes do not differ strongly from one another. This indicates that the *para* substituent on the *exo*‐cyclic aryl moiety does not have a significant influence on the electron accepting properties of these borafluorenes. This is best illustrated by the fact that the π‐donating dimethylamino group only leads to a cathodic shift of 0.15 V compared to a trifluoromethyl group. This is likely due to the nearly perpendicular arrangement of the *exo*‐aryl group with respect to the borafluorene backbone, that limits π‐conjugation leaving only inductive effects of the *exo*‐aryl moiety on the borafluorene core and the boron center. To investigate further the electronic properties of borafluorene ^**F**^ **Mes^F^Bf**, CoCp_2_ was chosen as a reducing agent (*E* ^0^′(CoCp_2_)=−1.3 V vs. Fc/Fc^+^).^122^ Thus, after addition of CoCp_2_, the yellowish THF solution of ^**F**^ **Mes^F^Bf** turned dark purple and an ESR measurement confirmed the presence of the borafluorene radical anion **[^F^Mes^F^Bf]^⋅−^** (Figure 6).

**Table 3:** Tabulated first reduction potential values of various three‐coordinate boron species.

Compound	First reduction potential *E*_1/2_ [V] vs. Fc/Fc^+^		Compound	First reduction potential *E*_1/2_ [V] vs. Fc/Fc^+^
this work				
**FMesFBfa**	−1.13			
**FMesFBfa**	−1.21			
**p‐NMe2‐FXylFBfa**	−1.28			
borafluorenes			free boroles	
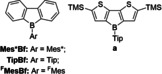			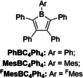	
Mes*Bf ^d^	−2.28^87^		PhBC_4_Ph_4_^a^	−1.61^117^
**TipBf** ^e^	−2.14^88^		MesBC_4_Ph_4_^a^	−1.69^85^, ^117^, ^118^
^**F**^ **MesBf** ^e^	−1.82^88^		^F^MesBC_4_Ph_4_^a^	−1.52^79^
**a^f^**	−1.98^119^			
diboraanthracene			Boron PAHs	
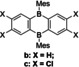			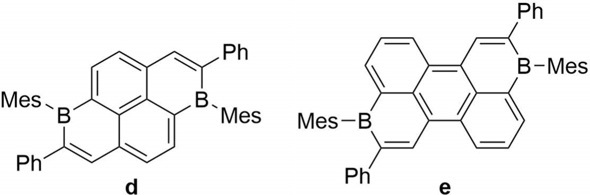	
**b** ^g^	−1.84^15^		**d** ^a^	−1.15^120^
**c** ^g^	−1.38^31^		**e** ^a^	−1.07^120^
triaryl boranes				
B(Mes)_3_^b^	−2.73^121^		B(3,5‐(CF_3_)_2_C_6_H_3_)_3_^c^	−1.61^102^
B(Mes)_2_(C_6_F_5_) ^b^	−2.10^121^		B(2,4‐(CF_3_)_2_C_6_H_3_)_3_^c^	−1.79^102^
B(Mes)(C_6_F_5_)_2_^b^	−1.72^121^		B(2,5‐(CF_3_)_2_C_6_H_3_)_3_^c^	−1.85^102^
B(C_6_F_5_)_3_^a^	−1.97^77^

Measurement conditions: [a] Platinum electrode, CH_2_Cl_2_ (solvent), [*n*Bu_4_N][PF_6_] (electrolyte); [b] Platinum electrode, THF (solvent), [*n*Bu_4_N][B(C_6_F_5_)_4_] (electrolyte); [c] Glassy carbon electrode, CH_2_Cl_2_ (solvent), [*n*Bu_4_N][B(C_6_F_5_)_4_] (electrolyte); [d] Glassy carbon electrode, THF (solvent), [*n*Bu_4_N][ClO_4_] (electrolyte); [e] Glassy carbon electrode, CH_2_Cl_2_ (solvent), [*n*Bu_4_N][PF_6_] (electrolyte); [f] Glassy carbon electrode, THF (solvent), [*n*Bu_4_N][PF_6_] (electrolyte); [g] Platinum electrode, THF (solvent), [*n*Bu_4_N][PF_6_] (electrolyte).

A THF solution of the radical anion **[^F^Mes^F^Bf]^⋅−^** shows a complex EPR signal centered at *g*_iso_=2.004 consisting of hyperfine splittings to boron (*a*(^11^B)=3.3 G; see non‐annulated borole derivatives (*a*(^11^B)=3.4–3.7 G)^85^, ^123^), the CF_3_ fluorine atoms (*a*(^19^F)=11.3 and 6.0 G) and the hydrogen atoms (*a*(^1^H)=6.1 and 2.9 G) of the borafluorene core. The relatively small boron hyperfine coupling together with the relatively large proton and fluorine hyperfine couplings indicate significant spin delocalization onto the benzene rings, to a greater extent than in other singly reduced borafluorenes.^87^

**Figure 7:**
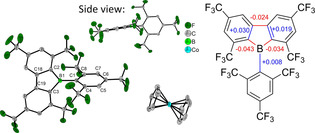
The solid‐state molecular structure of [^**F**^ **Mes^F^Bf**][CoCp_2_] determined by single‐crystal X‐ray diffraction at 100 K. All ellipsoids are drawn at the 50 % probability level. H atoms and THF solvent molecules are omitted for clarity. Only half of the symmetrically non‐equivalent molecules are shown. One of the CF_3_ groups on the borafluorene core is rotationally disordered and only the part with the higher occupancy (64 %) is shown here (left). The relevant changes in bond lengths of [^**F**^ **Mes^F^Bf**]^⋅−^ compared to the neutral starting material are shown at the right.

Crystals of the radical anion suitable for X‐ray diffraction were obtained by slow diffusion of pentane into a saturated THF solution (Figure 7). Upon reduction, a variety of changes in the structure are observed. As there are two independent molecules in the unit cell for the radical anion, both molecules are taken into account for comparison (Table S2, Supporting Information). The negative charge is apparently localized on the borafluorene core, as the *exo*‐aryl moiety is only slightly influenced, with almost all changes in bond length being within 3 esd's. Although the B−C1 bond length is slightly increased (Δ(B−C1)=+0.008 and +0.015 Å), both borafluorene B−C bonds (B−C2 and B−C3) are shortened (Δ(B−C2)=−0.034 and −0.025 Å; Δ(B−C3)=−0.043 and −0.040 Å). The neighboring borafluorene C−C bonds are elongated (Δ(C3−C19)=+0.030 and+0.027 Å; Δ(C2−C18)=+0.019 and +0.022 Å) and the C−C bond connecting the two borafluorene aryls is shortened (Δ(C18−C19)=−0.024 Å). In summary, the bond lengths within the five‐membered heterocycle equalize. This behavior was previously observed in the structures of borole radical anions and dianions.^85^ The one‐electron reduction also influences the shortest B−F distances which are elongated (mean Δ(B−F)= +0.278 Å).

### Photophysical properties

**Figure 8:**
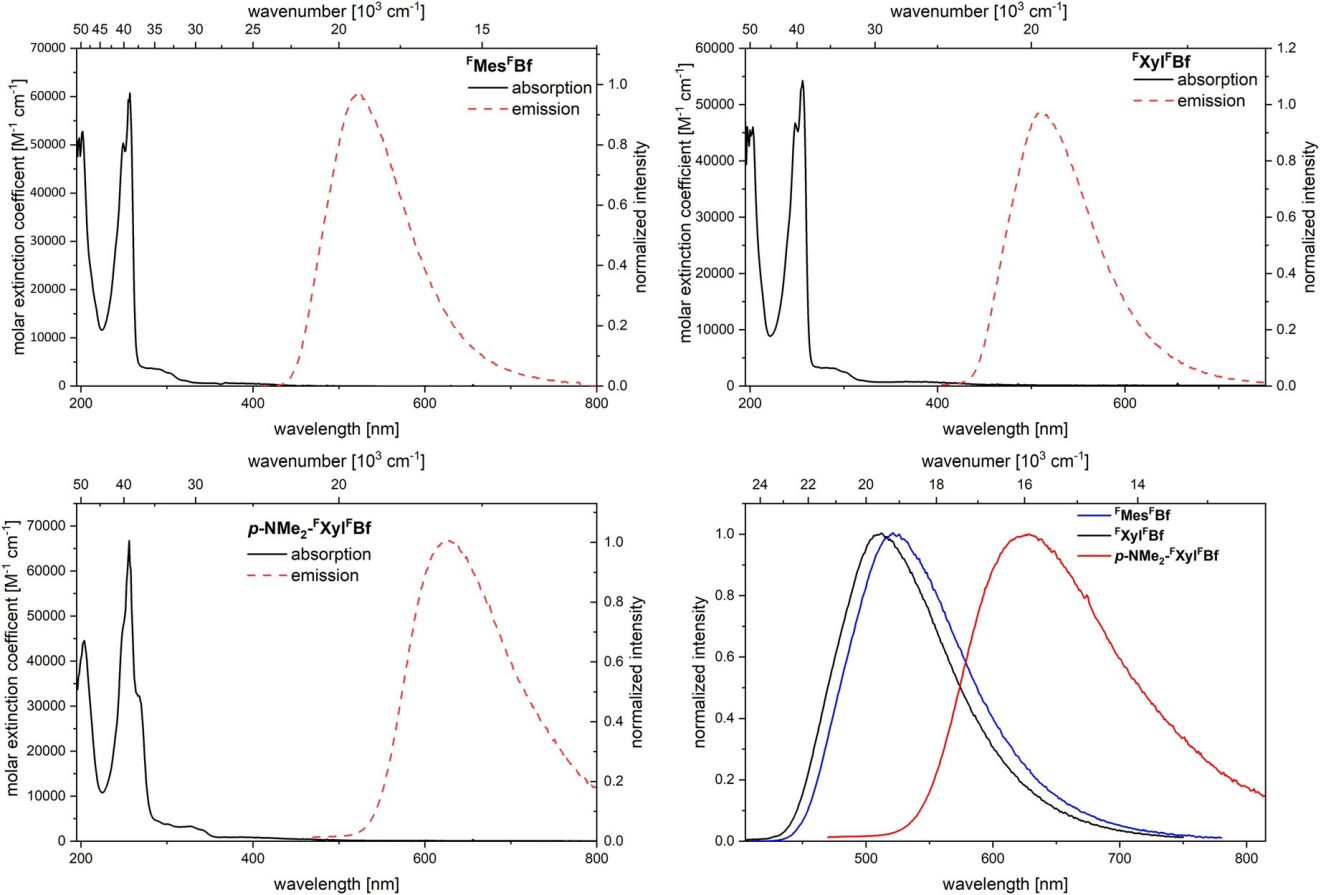
Absorption (black) and emission spectra (red) in hexane of ^**F**^ **Mes^F^Bf** (top left), ^**F**^ **Xyl^F^Bf** (top right) and ***p*** **‐NMe_2_‐^F^Xyl^F^Bf** (bottom left). For comparison, the emission spectra are plotted together (bottom right; ^**F**^ **Mes^F^Bf** (black), ^**F**^ **Xyl^F^Bf** (blue), ***p*** **‐NMe_2_‐^F^Xyl^F^Bf** (red)).

To obtain further insight into the electronic structure of the borafluorenes, absorption and emission spectra as well as quantum yields and excited‐state lifetimes were measured in hexane (Figure 8 and Table 4). Furthermore, ^**F**^ **Mes^F^Bf** was also studied in CH_2_Cl_2_ and in the solid state. Solvatochromic studies of ***p*** **‐NMe_2_‐^F^Xyl^F^Bf** could not be carried out, because no emission was detected in more polar solvents. This is most likely due to a further redshift of the emission, which in turn results in increased non‐radiative decay processes and thus a much lower quantum yield. A photophysical investigation of ^**F**^ **Mes^F^Bf⋅MeCN** is included in the Supporting Information (see also Figure S50 and Table S5).

**Table 4:** Photophysical data for borafluorenes ^**F**^ **Mes^F^Bf**, ^**F**^ **Xyl^F^Bf**, and ***p‐*** **NMe_2_‐^F^Xyl^F^Bf**.

Compound	Solvent		*λ*_abs_ [nm] (*ϵ* [10^3^ m ^−1^ cm^−1^]; log *ϵ*)		*λ*_em_ [nm]^[a]^		Apparent Stokes shift [10^3^ cm^−1^]		*Φ*_fl_		*τ* [ns] (rel %)		*τ_0_* [ns]		*k*_nr_ [10^7^ s^−1^]^[b]^		*k*_r_ [10^7^ s^−1^]^[c]^
^**F**^ **Mes^F^Bf**	hexane		290 (2.9; 3.46), 321 (0.8; 2.90), 400 (0.3; 2.48)		521		5.8		0.37		224		605		0.3		0.2
	CH_2_Cl_2_		395		540		6.8		0.18		151		835		0.1		0.005
	solid		405		527		6.0		0.06		173		2557		0.5		0.04
																	
^**F**^ **Xyl^F^Bf**	hexane		256 (54.2; 4.73), 290 (2.9; 3.46), 386 (0.4; 2.60)		510		6.3		0.30		249		820		0.3		0.1
																	
***p‐*** **NMe_2_‐^F^Xyl^F^Bf**	hexane		268 (32.3; 4.51), 326 (2.7; 3.43), 396 (0.3; 2.48)		627		9.3		0.03		9.2 (64 %), 1626 (36 %)		–		–		–

[a] Excited at the respective *λ*_abs max_ of the S_1_←S_0_ transition. [b] The non‐radiative rate constants were calculated from *k_nr_*=(1−*Φ*_fl_)/*τ*. [c] The radiative rate constants were calculated from *k*_r_=*Φ*_fl_/*τ*.

All borafluorenes exhibit very small extinction coefficients for their lowest‐energy absorption (*ϵ* **=**300–400 m ^−1^ cm^−1^; log *ϵ*=2.48–2.60) which can be classified as weakly allowed transitions,^124^ similar to those in previously reported boroles and borafluorenes. The lowest‐energy absorption of ^**F**^ **Xyl^F^Bf** (*λ*_abs, max_=386 nm) appears to be slightly hypsochromically shifted compared to ^**F**^ **Mes^F^Bf** (*λ*_abs, max_=400 nm) and ***p*** **‐NMe_2_‐^F^Xyl^F^Bf** (*λ*_abs, max_=396 nm) but, due to the broad absorption bands, this is difficult to determine accurately. All three borafluorenes exhibit broad, structureless emission bands. The emission maximum of ***p*** **‐NMe_2_‐^F^Xyl^F^Bf** is strongly bathochromically shifted (λ_em, max_=627 nm) compared to the two non‐donor‐substituted borafluorene derivatives (^**F**^ **Mes^F^Bf**: *λ*_em, max_=521 nm; ^**F**^ **Xyl^F^Bf**: *λ*_em, max_=510 nm), which indicates that the emission arises from an intramolecular charge‐transfer (ICT) transition. The quantum yields of ^**F**^ **Mes^F^Bf** (*Φ*_fl_=0.37; hexane) and ^**F**^ **Xyl^F^Bf** (*Φ*_fl_=0.30; hexane) are higher than most of the reported borafluorenes (ca. 0.1). In contrast, ***p*** **‐NMe_2_‐^F^Xyl^F^Bf** exhibits a very low quantum yield (*Φ*_fl_=0.03; hexane). To our surprise, ^**F**^ **Mes^F^Bf** and ^**F**^ **Xyl^F^Bf** exhibit very long fluorescent lifetimes in solution (^**F**^ **Mes^F^Bf**: *τ*=224 ns (hexane); *τ*=151 ns (CH_2_Cl_2_); ^**F**^ **Xyl^F^Bf**: *τ*=249 ns (hexane)) as well as in the solid state (^**F**^ **Mes^F^Bf**: *τ*=173 ns). Similar fluorescence lifetimes (116–150 ns) of borafluorenes with bulky *exo*‐aryl moieties were previously observed by Rupar and co‐workers.^89^ This results in exceptionally long natural lifetimes, *τ*_0_, uncommon for organic molecules, for which fluorescence usually takes place on a nanosecond timescale. There are, however, some exceptions such as pyrene.^36^, ^125^, ^126^ This indicates a forbidden process. It is very interesting that even though the radiative rate constants are small for organic chromophores, the non‐radiative rate constants are of the same order, resulting in moderate quantum yields. This is likely a result of the high rigidity of the systems. In contrast, ***p*** **‐NMe_2_‐^F^Xyl^F^Bf** exhibits two different radiative decay processes, a prompt (*τ*=9.2 ns) and a delayed (*τ*=1.6 μs) one. This can be caused by different processes namely TTA (triplet–triplet annihilation)^127^ or TADF (thermally activated delayed fluorescence).^66^, ^128^, ^129^, ^130^, ^131^, ^132^, ^133^, ^134^, ^135^, ^136^, ^137^, ^138^, ^139^, ^140^, ^141^, ^142^ Due to the low concentrations (≤10^−5^ m) of the compound employed, the lack of dependence on the concentration and the temperature dependence of the lifetime, we can attribute the observed behavior to TADF. The mechanism for TADF is based on a reverse intersystem crossing process (rISC) between the lowest‐energy triplet state (T_1_) and excited singlet state (S_1_) of a molecule. In order for this to occur, the energy difference between S_1_ and T_1_ (Δ*E*_S–T_) has to be sufficiently small. The most common structures to exhibit this phenomenon are twisted dipolar systems with spatially separated HOMO and LUMO such as D(donor)–π–A(acceptor) compounds. This structural motif is also found in ***p*** **‐NMe_2_‐^F^Xyl^F^Bf**. The singlet–triplet gap (Δ*E*_S‐T_) can be easily determined experimentally if phosphorescence can be observed. However, for ***p*** **‐NMe_2_‐^F^Xyl^F^Bf**, even at 77 K in a frozen glass matrix of 2‐MeTHF, no phosphorescence was observed. However, Δ*E*_S–T_ can also be calculated once the rate constant of the rISC process (*k*_rISC_) is obtained, which is given by the Arrhenius Equation (1), or Equation (2) as derived by Dias et al.^143^

(1) $$k_{rISC}=A\exp\left(-\frac{\Delta E_{S-T}}{kT}\right)$$

(2) $$k_{rISC}=\frac{\Phi_{\mathrm{rISC}}}{\tau_{\mathrm{DF}}}\left(\frac{\Phi_{\mathrm{PF}}+\Phi_{\mathrm{DF}}}{\Phi_{\mathrm{PF}}}\right)$$

with *Φ*_DF_, *Φ*_PF_ and *Φ*_rISC_ being the quantum yields of the delayed and prompt fluorescence and the reverse intersystem crossing, respectively. Given that no phosphorescence was observed at 77 K, but delayed fluorescence was, it can be assumed that Δ*E*_S–T_ is small and, as such, the rate of reverse intersystem crossing is very high, i.e.,

$\Phi_{rISC}=\frac{k_{\mathrm{rISC}}}{k_{\mathrm{rISC}}+k_{\mathrm{IC}}^{T}+k_{\mathrm{PH}}}\approx 1$

and, thus, Equation (2) can be modified to give Equation (3).

(3) $$k_{\mathrm{rISC}}=\frac{1}{\tau_{\mathrm{DF}}}\left(\frac{\Phi_{\mathrm{PF}}{+\Phi }_{\mathrm{DF}}}{\Phi_{\mathrm{PF}}}\right)=\frac{1}{\tau_{\mathrm{DF}}}\left(1+\frac{\Phi_{\mathrm{DF}}}{\Phi_{\mathrm{PF}}}\right)=\frac{1}{\tau_{\mathrm{DF}}}+\frac{1}{\tau_{\mathrm{DF}}}{}^{*}\frac{\Phi_{\mathrm{DF}}}{\Phi_{\mathrm{PF}}}$$

The ratio *Φ*_DF_/*Φ*_PF_ can be ascertained from time‐resolved measurements via Equation (4),

(4) $$\frac{\Phi_{\mathrm{DF}}}{\Phi_{\mathrm{PF}}}=\frac{B_{\mathrm{DF}}\tau_{\mathrm{DF}}}{B_{\mathrm{PF}}\tau_{\mathrm{DF}}}$$

with *B*_DF_ and *B*_PF_ being the pre‐exponential fitting parameters of the time‐resolved fluorescence lifetime measurements. As such, equation (1) can be written as Equation (5) where all parameters can be obtained from the time‐resolved fluorescence decay.

(5) $$k_{\mathrm{rISC}}=\;A\exp\left(-\frac{\Delta E_{S-T}}{kT}\right)=\frac{1}{\tau_{\mathrm{DF}}}+\frac{1}{\tau_{\mathrm{DF}}}\frac{A_{\mathrm{DF}}\tau_{\mathrm{DF}}}{A_{\mathrm{PF}}\tau_{\mathrm{DF}}}$$

**Figure 9:**
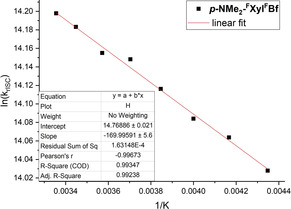
Arrhenius plot of ln(*k*_rISC_) of ***p*** **‐NMe_2_‐^F^Xyl^F^Bf** (determined from temperature‐dependent lifetime measurements) vs. 1/*T*.

Lifetimes were obtained at temperatures between 300 K and 230 K in methylcyclohexane. From the slope of a plot of ln(*k*_rISC_) versus 1/*T* (Figure 9), we obtain Δ*E*_S–*T*_=15 meV, which is comparable to values previously reported for TADF emitters.^143^

### DFT and time‐dependent (TD)‐DFT studies

**Figure 10:**
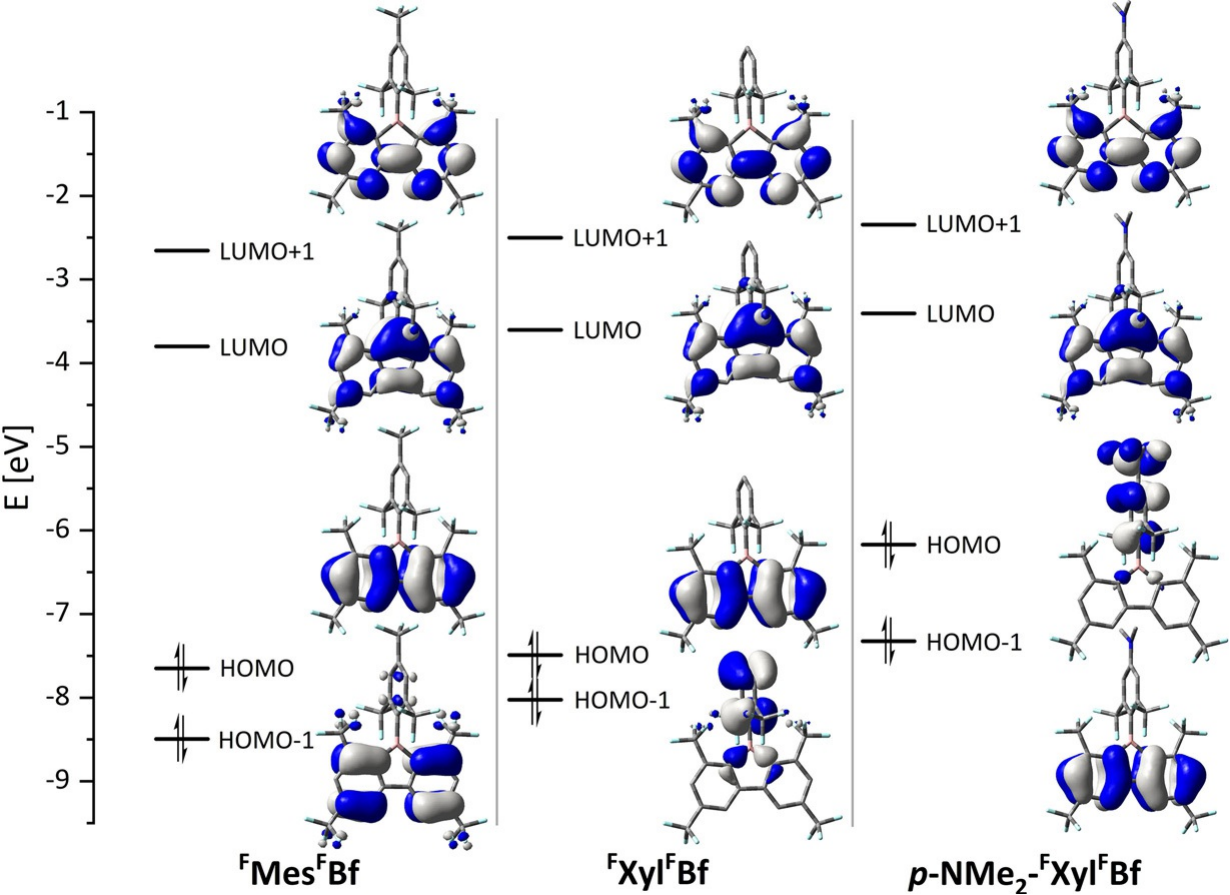
Frontier molecular orbitals of ^**F**^ **Mes^F^Bf** (left), ^**F**^ **Xyl^F^Bf** (middle), and ***p*** **‐NMe_2_‐^F^Xyl^F^Bf** (right) calculated at the B3LYP/6–31+G(d) level of theory.

Using the crystal structures as the starting geometries, the ground‐state (GS) structures were optimized using DFT calculations at the B3LYP/6‐31G+(d) level of theory. For ^**F**^ **Mes^F^Bf** and ^**F**^ **Xyl^F^Bf**, the optimized ground‐state structures exhibit C_*s*_ and *C*_2*v*_ symmetries, respectively. For ***p*** **‐NMe_2_‐^F^Xyl^F^Bf**, optimization of the GS structure with *C*_2*v*_ symmetry did not lead to a global minimum but rather to a saddle point (1 imaginary frequency remained). However, given that the *C*_1_ structure is very close to the *C*_2*v*_ symmetry one, in both geometry and energy, and exhibits almost the same transition dipole moments, the symmetry descriptors will be used as it simplifies the discussion. The optimized structures reproduce the geometries, bond lengths, angles, and shortest B−F contacts of the crystal structures reasonably well. However, as compared to the crystal structures, the optimized structures do not exhibit bending of the *exo*‐aryl out of the plane of the borafluorene backbone. Given that this torsion arises from solid‐state interactions, this is to be expected. The highest occupied molecular orbital (HOMO) and lowest unoccupied molecular orbital (LUMO) energies increase from ^**F**^ **Mes^F^Bf** to ^**F**^ **Xyl^F^Bf** to ***p*** **‐NMe_2_‐^F^Xyl^F^Bf** (Figure 10 and Table 5). The calculated LUMO energies fit well with the LUMO energies estimated from the reduction potentials obtained through cyclic voltammetry. Due to the very broad nature of the lowest‐energy absorption band, the HOMO energies of ^**F**^ **Mes^F^Bf** and ^**F**^ **Xyl^F^Bf** were not calculated from the experimental data.

**Table 5:** Frontier molecular p‐orbital energies [eV] and symmetries of ^**F**^ **Mes^F^Bf**, ^**F**^ **Xyl^F^Bf**, and ***p‐*** **NMe_2_‐^F^Xyl^F^Bf** calculated at the B3LYP/6–31+G(d) level.

	^**F**^ **Mes^F^Bf**			^**F**^ **Xyl^F^Bf**			***p‐*** **NMe_2_‐^F^Xyl^F^Bf**		
symmetry	*C_s_*			*C*_2*v*_			*C*_1_	*C*_2*v*_	
	calc.	exp.^[a]^		calc.	exp.^[a]^		calc.	calc.	exp.^[a]^
LUMO+1 [eV] (sym.)	−2.66 (A′′)			−2.50 (B_1_)			−2.34 (A_1_)	−2.32 (B_1_)	
LUMO [eV] (sym.)	−3.80 (A′′)	−4.02		−3.60 (B_1_)	−3.95		−3.41 (A_1_)	−3.42 (B_1_)	−3.88
HOMO [eV] (sym.)	−7.65 (A′′)			−7.49 (A_2_)			−6.17 (A_1_)	−6.16 (B_2_)	−6.11
HOMO−1 [eV] (sym.)	−8.49 (A′)			−8.03 (B_2_)			−7.32 (A_1_)	−7.41 (A_2_)

[a] Determined from the half‐wave potentials: HOMO=−(5.16+*E* ${}_{1/2}$_, ox_) eV, LUMO=−(5.16+*E* ${}_{1/2}$_, red_) eV.^122^, ^144^, ^145^

The LUMOs are all localized on the borafluorene moieties with their largest components on boron, and the energies differ by only 0.2–0.4 eV. For ^**F**^ **Mes^F^Bf** and ^**F**^ **Xyl^F^Bf** the HOMOs are also localized on the borafluorene moieties with boron lying on a nodal plane, and are energetically similar (Δ*E*=0.16 eV). The HOMO of ***p*** **‐NMe_2_‐^F^Xyl^F^Bf** is localized on the *exo*‐aryl moiety and lies about 1.4 eV higher in energy than the HOMOs of ^**F**^ **Mes^F^Bf** and ^**F**^ **Xyl^F^Bf**. This is due to the electron‐donating effect of the *para*‐dimethylamino‐group that increases the energy of the *exo*‐aryl fragment MO thereby raising it above the borafluorene‐centered orbital which is now HOMO−1. For both ^**F**^ **Mes^F^Bf** and ^**F**^ **Xyl^F^Bf**, HOMO−1 is localized on the *exo*‐aryl moiety; however, due to the *para*‐CF_3_ group, the HOMO−1 of ^**F**^ **Mes^F^Bf** is about 0.5 eV lower in energy. Based on the optimized ground‐state structures the nucleus‐independent chemical shift (NICS) values of the borafluorenes were calculated (Table 6).

**Table 6:** NICS(1)_zz_ values of the borole moiety of ^**F**^ **Mes^F^Bf**, ^**F**^ **Xyl^F^Bf**, ***p‐*** **NMe_2_‐^F^Xyl^F^Bf**, and TipBf^119^ calculated at the B3LYP/6–311+G(d) level.

Compound	NICS(1)_zz_	
	calcd	Lit.
^**F**^ **Mes^F^Bf**	20.7	
^**F**^ **Xyl^F^Bf**	20.2	
***p‐*** **NMe_2_‐^F^Xyl^F^Bf**	20.0	
**TipBf**	24.3	24.5^119^

It is apparent that the perfluoroalkylated borafluorenes exhibit lower NICS(1)_zz_ values as compared to that of the borafluorene **TipBf** which does not contain CF_3_ groups. This suggests a higher degree of delocalization of the electron density over the borafluorene backbone in our compounds. Thus, the antiaromatic character is less than in non‐trifluoromethylated borafluorenes. The optimized structures were then used for TD‐DFT calculations to simulate the absorption spectra. Time‐dependent DFT calculations on ^**F**^ **Mes^F^Bf** and ^**F**^ **Xyl^F^Bf** were carried out at the B3LYP/6–31+G(d) level of theory whereas for the donor substituted ***p*** **‐NMe_2_‐^F^Xyl^F^Bf** the Coulomb‐attenuated functional CAM‐B3LYP was employed using the same basis set (Table 7), because CAM‐B3LYP is better suited to systems involving charge transfer.^146^, ^147^ Furthermore, the optimized S_1_‐state geometries of ^**F**^ **Mes^F^Bf** and ^**F**^ **Xyl^F^Bf** were obtained. In order to characterize the nature of the transition the overlap coefficients (*Λ*) were determined.^146^ The calculated lowest‐energy absorptions of the borafluorenes fit well with the experimental values. For all compounds, the lowest‐energy transitions are predominantly of HOMO to LUMO character.

**Table 7:** Lowest‐energy and highest oscillator‐strength absorptions and emissions of ^**F**^ **Mes^F^Bf** and ^**F**^ **Xyl^F^Bf** calculated at the B3LYP/6–31+G(d) level of theory and lowest‐energy absorptions of ***p‐*** **NMe_2_‐^F^Xyl^F^Bf** calculated at the CAM‐B3LYP/6–31+G(d) level of theory.

Compound	State	*E* [eV]	*λ* [nm]	*λ*_exp_ [nm]	*f*	Symmetry	Major contributions	*Λ*
^**F**^ **Mes^F^Bf** (*C_s_*)	S_1_←S_0_	3.05	406	400	0.0005	A′	HOMO→LUMO (99 %)	0.65
	S_2_←S_0_	3.90	318		0	A“	HOMO−1→LUMO (99 %)	0.25
	S_7_←S_0_	4.80	258	257	0.8333	A′	HOMO−2→LUMO (24 %), HOMO→LUMO+1 (70 %)	0.73
	S_1_→S_0_	2.22	559	521	0.0038	A	H‐SOMO←L‐SOMO (99 %)	
								
^**F**^ **Xyl^F^Bf** (*C*_2*v*_)	S_1_←S_0_	3.09	401	385	0.0006	B_2_	HOMO→LUMO (99 %)	0.66
	S_2_←S_0_	3.64	340		0	A_2_	HOMO−1→LUMO (99 %)	0.23
	S_6_←S_0_	4.81	258	256	0.8413	B_2_	HOMO−3→LUMO (28 %), HOMO→LUMO+1 (68 %)	0.74
	S_1_→S_0_	2.34	531	510	0.0030	A	H‐SOMO←L‐SOMO (99 %)	
								
***p‐*** **NMe_2_‐^F^Xyl^F^Bf** (*C*_1_)	S_1_←S_0_	3.25	382	396	0	A	HOMO→LUMO (92 %)	0.15
	S_2_←S_0_	3.46	358	326	0.0006	A	HOMO−1→LUMO (97 %)	0.67
	S_3_←S_0_	4.25	292	270	0.0491	A	HOMO→L+2 (70 %), HOMO→L+3 (24 %)	0.49
	S_8_←S_0_	5.11	243	256	0.6022	A	HOMO→L+4 (86 %)	0.71

**Figure 11:**
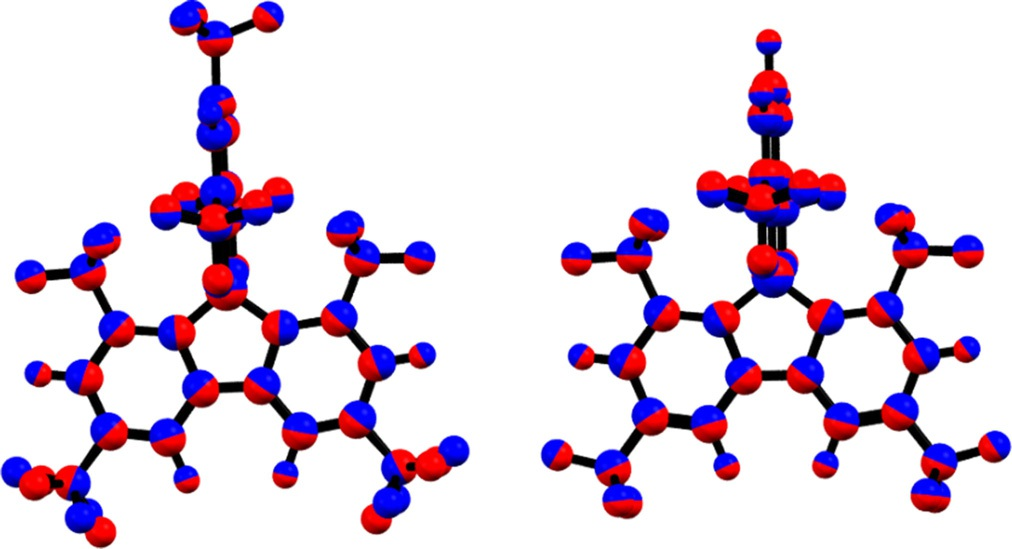
Overlap of the optimized ground state (blue) and S_1_ (red) geometries of ^**F**^ **Mes^F^Bf** (left) and ^**F**^ **Xyl^F^Bf** (right).

For both ^**F**^ **Mes^F^Bf** and ^**F**^ **Xyl^F^Bf** these can be classified as locally excited (LE) transitions (*Λ*≈0.65) as both HOMO and LUMO are localized on the borafluorene backbone. This is surprising, as LE transitions usually exhibit high extinction coefficients and ^**F**^ **Mes^F^Bf** and ^**F**^ **Xyl^F^Bf** exhibit weakly allowed lowest energy absorptions. However, the calculated oscillator strengths of the S_1_←S_0_ transitions of ^**F**^ **Mes^F^Bf** and ^**F**^ **Xyl^F^Bf** are also very small. From the symmetries of the frontier molecular orbitals it is possible to determine whether these transitions are forbidden by symmetry. The symmetries of the HOMOs of ^**F**^ **Mes^F^Bf** (C_s_) and ^**F**^ **Xyl^F^Bf** (*C*_2*v*_) are A′′ and A_2_, respectively, and the LUMO symmetries are A′′ and B_1_. Transitions are allowed by symmetry if the initial and final states multiplied by the *x*‐, *y*‐, and *z*‐characters of the electronic dipole operator contain the totally symmetric irreducible representation (*C_s_*: A′ and *C*_2*v*_: A_1_). For ^**F**^ **Mes^F^Bf** (*C_s_*), the lowest‐energy transition is forbidden in the *z*‐direction, whereas *x* and *y* are allowed, making the transition an allowed transition. For ^**F**^ **Xyl^F^Bf** (*C*_2*v*_), the *x*‐ and *z*‐directions are forbidden, whereas the *y*‐direction is allowed, making the transition an allowed transition. However, for both molecules, the dipole moment is oriented along the *z*‐axis, resulting in a very small transition dipole moment in the *x*‐ and *y*‐directions, which results in very low oscillator strengths. So, the lowest‐energy transitions are allowed, but exhibit only small changes in dipole moment resulting in weak absorptions. This can be, in part, attributed to the boron center, because its contribution to the LUMO gives the transition a π–n character. The S_2_←S_0_ transitions, in both cases, are predominantly HOMO−1 to LUMO transitions. The HOMO−1 of ^**F**^ **Mes^F^Bf** and ^**F**^ **Xyl^F^Bf** are of A′ and B_2_ symmetry, respectively, and, thus, are symmetry forbidden. Both ^**F**^ **Mes^F^Bf** and ^**F**^ **Xyl^F^Bf**, exhibit large oscillator strengths for their S_7_←S_0_ and S_6_←S_0_ transitions, respectively. In both cases, these transitions have predominantly HOMO to LUMO+1 character. Both HOMO and LUMO+1 are delocalized over the borafluorene backbone without contributions from the boron center. The optimized S_1_ geometries of ^**F**^ **Mes^F^Bf** and ^**F**^ **Xyl^F^Bf** differ only slightly from their ground‐state structures (Figure 11).

In comparison, in the S_1_ structure of ^**F**^ **Mes^F^Bf**, only the *para*‐CF_3_ groups on the borafluorene backbone are rotated and in both ^**F**^ **Mes^F^Bf** and ^**F**^ **Xyl^F^Bf** the *ortho*‐CF_3_ groups on the *exo*‐aryl are slightly bent away from the boron center. The calculated emission maxima of ^**F**^ **Mes^F^Bf** and ^**F**^ **Xyl^F^Bf**, fit the experimental data in hexane and also exhibit very low oscillator strengths. Even though the optimized structures do not exhibit a higher symmetry it can be assumed that a similar phenomenon as for the absorption takes place and is the reason for the observed long lifetimes. Interestingly, as previously discussed, the reasonably high quantum yields observed are due to extremely slow non‐radiative decay processes.

The photophysical properties of ***p*** **‐NMe_2_‐^F^Xyl^F^Bf** differ strongly from those of ^**F**^ **Mes^F^Bf** and ^**F**^ **Xyl^F^Bf**. This is, in part, due to the fact that the nature of the lowest‐energy absorption has CT rather than LE character (*Λ*=0.15). This is not surprising, given that ***p*** **‐NMe_2_‐^F^Xyl^F^Bf** is a donor–acceptor system. The S_1_←S_0_ transition of ***p*** **‐NMe_2_‐^F^Xyl^F^Bf** exhibits an oscillator strength of 0. Using the optimized structure of *C*_2*v*_ symmetry as an approximation, it becomes apparent that this transition is symmetry forbidden and, furthermore, the overlap between HOMO and LUMO is minuscule due to the nearly perpendicular arrangement of the *exo*‐aryl group with respect to the borafluorene core. The HOMO is of B_2_ symmetry and the LUMO has B_1_ symmetry. This is the same as for the S_2_←S_0_ transitions of ^**F**^ **Mes^F^Bf** and ^**F**^ **Xyl^F^Bf**, because the HOMO and HOMO−1 are inverted compared to those of ***p*** **‐NMe_2_‐^F^Xyl^F^Bf**. For ***p*** **‐NMe_2_‐^F^Xyl^F^Bf**, however, the S_2_←S_0_ transition is allowed, but analogously to the ^**F**^ **Mes^F^Bf** and ^**F**^ **Xyl^F^Bf** cases, exhibits a very low oscillator strength. This explains the low extinction coefficient observed for the lowest‐energy absorption of ***p*** **‐NMe_2_‐^F^Xyl^F^Bf**. Furthermore, we optimized the S_1_ structure of ***p*** **‐NMe_2_‐^F^Xyl^F^Bf** as well as its T_1_ structure in order to calculate the S_1_–T_1_ energy gap. Both optimizations were carried out using the PCM solvent‐correction model due to the charge‐transfer nature of the transitions and the high dipole moment of both S_1_ and T_1_. Comparing the energies of both structures results in Δ*E*_S–T_=423 meV which is almost 30 times higher than the experimentally determined gap. It is noteworthy that the experimental determination of the gap is highly flawed due to approximations as well as unpredictable solvent effects at lower temperature. However, this should still give a good estimate, but the calculations fail to match the experimental value at this level of theory, illustrating the difficulty of predicting phenomena such as TADF accurately.

## Conclusions

Herein, we reported the synthesis and properties of three trifluoromethylated borafluorenes ^**F**^ **Mes^F^Bf**, ^**F**^ **Xyl^F^Bf**, and ***p*** **‐NMe_2_‐^F^Xyl^F^Bf**. The copper‐catalyzed homocoupling of boronate esters provides a convenient route to 2,2′‐dibromobiphenyl derivatives, which can be lithiated and then reacted with stable and accessible aryl‐BF_3_K salts for the synthesis of borafluorenes. All of the borafluorenes exhibit a rigid geometry with the *exo*‐aryl group lying perpendicular to the borafluorene plane. All three borafluorenes exhibit exceptionally positively shifted reduction potentials, emphasizing the electron‐withdrawing nature of the CF_3_ groups. This allowed us to use a mild reducing agent (CoCp_2_ *E* ^0^=−1.3 eV vs. Fc/Fc^+^) to reduce the most anodically shifted borafluorene ^**F**^ **Mes^F^Bf**. The resulting radical anion of ^**F**^ **Mes^F^Bf** exhibits a strong delocalization of the additional electron over the borafluorene backbone as evidenced by EPR spectroscopy and its solid‐state structure. The trifluoromethylated borafluorenes exhibit unusually long excited‐state lifetimes and weakly allowed lowest‐energy transitions. For ^**F**^ **Mes^F^Bf** and ^**F**^ **Xyl^F^Bf**, this is the result of the transitions being forbidden in the *z*‐direction which coincides with the dipole moment and the transition dipole moment being negligible in the *x*‐ and *y*‐directions. The same is apparently true for their emissions, as both compounds exhibit fluorescence lifetimes of *τ*>200 ns in hexane. Even with small oscillator strengths, the two compounds exhibit fluorescence quantum yields of 0.37 and 0.30, respectively, because their rigidity results in exceptionally slow non‐radiative decay. In contrast, the twisted donor–acceptor system ***p*** **‐NMe_2_‐^F^Xyl^F^Bf** has a symmetry forbidden lowest‐energy transition and exhibits TADF, with a singlet–triplet energy gap Δ*E*_S–T_ experimentally determined to be only 15 meV. The compounds ^**F**^ **Mes^F^Bf** and ^**F**^ **Xyl^F^Bf** are highly stable towards hydrolysis, which makes them interesting potential building blocks for organic materials.

## Conflict of interest

The authors declare no conflict of interest.

## Supporting information

As a service to our authors and readers, this journal provides supporting information supplied by the authors. Such materials are peer reviewed and may be re‐organized for online delivery, but are not copy‐edited or typeset. Technical support issues arising from supporting information (other than missing files) should be addressed to the authors.

Supplementary

## References

[1] C. D. Entwistle , T. B. Marder , Angew. Chem. Int. Ed. 2002, 41, 2927–2931; 10.1002/1521-3773(20020816)41:16<2927::AID-ANIE2927>3.0.CO;2-L 12203415

[2] C. D. Entwistle , T. B. Marder , Chem. Mater. 2004, 16, 4574–4585.

[3] S. Yamaguchi , A. Wakamiya , Pure Appl. Chem. 2006, 78, 1413.

[4] F. Jäkle , Coord. Chem. Rev. 2006, 250, 1107–1121.

[5] R. Stahl , C. Lambert , C. Kaiser , R. Wortmann , R. Jakober , Chem. Eur. J. 2006, 12, 2358–2370. 16358349 10.1002/chem.200500948

[6] M. Elbing , G. C. Bazan , Angew. Chem. Int. Ed. 2008, 47, 834–838; 10.1002/anie.200703722 18081115

[7] Z. M. Hudson , S. Wang , Acc. Chem. Res. 2009, 42, 1584–1596. 19558183 10.1021/ar900072u

[8] C. R. Wade , A. E. J. Broomsgrove , S. Aldridge , F. P. Gabbaï , Chem. Rev. 2010, 110, 3958–3984. 20540560 10.1021/cr900401a

[9] Z. M. Hudson , S. Wang , Dalton Trans. 2011, 40, 7805–7816. 21603687 10.1039/c1dt10292c

[10] A. Wakamiya , S. Yamaguchi , Bull. Chem. Soc. Jpn. 2015, 88, 1357–1377.

[11] L. Ji , S. Griesbeck , T. B. Marder , Chem. Sci. 2017, 8, 846–863. 28572897 10.1039/c6sc04245g PMC5452272

[12] S.-Y. Li , Z.-B. Sun , C.-H. Zhao , Inorg. Chem. 2017, 56, 8705–8717. 28165231 10.1021/acs.inorgchem.6b02847

[13] G. Turkoglu , M. E. Cinar , T. Ozturk , Molecules 2017, 22, 1522. 10.3390/molecules22091522 PMC6151606 28902157

[14] C. Dou , S. Saito , K. Matsuo , I. Hisaki , S. Yamaguchi , Angew. Chem. Int. Ed. 2012, 51, 12206–12210; 10.1002/anie.201206699 23081889

[15] C. Hoffend , M. Diefenbach , E. Januszewski , M. Bolte , H. W. Lerner , M. C. Holthausen , M. Wagner , Dalton Trans. 2013, 42, 13826–13837. 23752707 10.1039/c3dt51035b

[16] A. Escande , M. J. Ingleson , Chem. Commun. 2015, 51, 6257–6274. 10.1039/c5cc00175g 25680083

[17] V. M. Hertz , N. Ando , M. Hirai , M. Bolte , H.-W. Lerner , S. Yamaguchi , M. Wagner , Organometallics 2016, 35, 2512–2519.

[18] B. Su , R. Kinjo , Synthesis 2017, 49, 2985–3034.

[19] E. von Grotthuss , A. John , T. Kaese , M. Wagner , Asian J. Org. Chem. 2018, 7, 37–53.

[20] Y. Su , R. Kinjo , Chem. Soc. Rev. 2019, 48, 3613–3659. 30977491 10.1039/c9cs00072k

[21] N. Matsumi , K. Naka , Y. Chujo , J. Am. Chem. Soc. 1998, 120, 5112–5113.

[22] S. Yamaguchi , S. Akiyama , K. Tamao , J. Am. Chem. Soc. 2000, 122, 6335–6336.

[23] W.-L. Jia , D. Song , S. Wang , J. Org. Chem. 2003, 68, 701–705. 12558388 10.1021/jo020695h

[24] A. Wakamiya , T. Ide , S. Yamaguchi , J. Am. Chem. Soc. 2005, 127, 14859–14866. 16231940 10.1021/ja0537171

[25] I. Yamaguchi , B.-J. Choi , T.-A. Koizumi , K. Kubota , T. Yamamoto , Macromolecules 2007, 40, 438–443.

[26] U. Megerle , F. Selmaier , C. Lambert , E. Riedle , S. Lochbrunner , Phys. Chem. Chem. Phys. 2008, 10, 6245–6251. 18936848 10.1039/b806131a

[27] A. Lorbach , M. Bolte , H. Li , H.-W. Lerner , M. C. Holthausen , F. Jäkle , M. Wagner , Angew. Chem. Int. Ed. 2009, 48, 4584–4588; 10.1002/anie.200901226 19455524

[28] L. Weber , V. Werner , M. A. Fox , T. B. Marder , S. Schwedler , A. Brockhinke , H.-G. Stammler , B. Neumann , Dalton Trans. 2009, 2823–2831. 19333506 10.1039/b821208b

[29] L. Weber , D. Eickhoff , T. B. Marder , M. A. Fox , P. J. Low , A. D. Dwyer , D. J. Tozer , S. Schwedler , A. Brockhinke , H.-G. Stammler , B. Neumann , Chem. Eur. J. 2012, 18, 1369–1382. 22213064 10.1002/chem.201102059

[30] Z. Zhou , A. Wakamiya , T. Kushida , S. Yamaguchi , J. Am. Chem. Soc. 2012, 134, 4529–4532. 22369126 10.1021/ja211944q

[31] C. Reus , S. Weidlich , M. Bolte , H.-W. Lerner , M. Wagner , J. Am. Chem. Soc. 2013, 135, 12892–12907. 23899377 10.1021/ja406766e

[32] J. Yoshino , Y. Nakamura , S. Kunitomo , N. Hayashi , H. Higuchi , Tetrahedron Lett. 2013, 54, 2817–2820.

[33] X. Yin , J. Chen , R. A. Lalancette , T. B. Marder , F. Jäkle , Angew. Chem. Int. Ed. 2014, 53, 9761–9765; 10.1002/anie.201403700 25044554

[34] A. Ito , K. Kawanishi , E. Sakuda , N. Kitamura , Chem. Eur. J. 2014, 20, 3940–3953. 24644157 10.1002/chem.201304207

[35] Z. Zhang , R. M. Edkins , J. Nitsch , K. Fucke , A. Eichhorn , A. Steffen , Y. Wang , T. B. Marder , Chem. Eur. J. 2015, 21, 177–190. 25413782 10.1002/chem.201405621

[36] J. Merz , J. Fink , A. Friedrich , I. Krummenacher , H. H. Al Mamari , S. Lorenzen , M. Haehnel , A. Eichhorn , M. Moos , M. Holzapfel , H. Braunschweig , C. Lambert , A. Steffen , L. Ji , T. B. Marder , Chem. Eur. J. 2017, 23, 13164–13180. 28718975 10.1002/chem.201702594

[37] S. Griesbeck , E. Michail , C. Wang , H. Ogasawara , S. Lorenzen , L. Gerstner , T. Zang , J. Nitsch , Y. Sato , R. Bertermann , M. Taki , C. Lambert , S. Yamaguchi , T. B. Marder , Chem. Sci. 2019, 10, 5405–5422. 31217943 10.1039/c9sc00793h PMC6549598

[38] X. Jia , J. Nitsch , L. Ji , Z. Wu , A. Friedrich , F. Kerner , M. Moos , C. Lambert , T. B. Marder , Chem. Eur. J. 2019, 25, 10845–10857. 31210396 10.1002/chem.201902258

[39] J. Merz , A. Steffen , J. Nitsch , J. Fink , C. B. Schürger , A. Friedrich , I. Krummenacher , H. Braunschweig , M. Moos , D. Mims , C. Lambert , T. B. Marder , Chem. Sci. 2019, 10, 7516–7534. 31588303 10.1039/c9sc02420d PMC6761871

[40] J. He , F. Rauch , A. Friedrich , D. Sieh , T. Ribbeck , I. Krummenacher , H. Braunschweig , M. Finze , T. B. Marder , Chem. Eur. J. 2019, 25, 13777–13784. 31471986 10.1002/chem.201903118 PMC6899742

[41] Z. Yuan , N. J. Taylor , T. B. Marder , I. D. Williams , S. K. Kurtz , L.-T. Cheng , J. Chem. Soc. Chem. Commun. 1990, 1489–1492.

[42] Z. Yuan , N. J. Taylor , Y. Sun , T. B. Marder , I. D. Williams , L.-T. Cheng , J. Organomet. Chem. 1993, 449, 27–37.

[43] Z. Yuan , N. J. Taylor , R. Ramachandran , T. B. Marder , Appl. Organomet. Chem. 1996, 10, 305–316.

[44] Z.-Q. Liu , Q. Fang , D. Wang , G. Xue , W.-T. Yu , Z.-S. Shao , M.-H. Jiang , Chem. Commun. 2002, 2900–2901. 10.1039/b207210f 12478803

[45] Z.-Q. Liu , Q. Fang , D. Wang , D.-X. Cao , G. Xue , W.-T. Yu , H. Lei , Chem. Eur. J. 2003, 9, 5074–5084. 14562325 10.1002/chem.200304833

[46] M. Charlot , L. Porres , C. D. Entwistle , A. Beeby , T. B. Marder , M. Blanchard-Desce , Phys. Chem. Chem. Phys. 2005, 7, 600–606. 19787875 10.1039/b416605a

[47] Z. Yuan , C. D. Entwistle , J. C. Collings , D. Albesa-Jové , A. S. Batsanov , J. A. K. Howard , N. J. Taylor , H. M. Kaiser , D. E. Kaufmann , S.-Y. Poon , W.-Y. Wong , C. Jardin , S. Fathallah , A. Boucekkine , J.-F. Halet , T. B. Marder , Chem. Eur. J. 2006, 12, 2758–2771. 16429474 10.1002/chem.200501096

[48] C. D. Entwistle , J. C. Collings , A. Steffen , L.-O. Pålsson , A. Beeby , D. Albesa-Jove , J. M. Burke , A. S. Batsanov , J. A. K. Howard , J. A. Mosely , S.-Y. Poon , W.-Y. Wong , F. Ibersiene , S. Fathallah , A. Boucekkine , J.-F. Halet , T. B. Marder , J. Mater. Chem. 2009, 19, 7532–7544.

[49] J. C. Collings , S.-Y. Poon , C. Le Droumaguet , M. Charlot , C. Katan , L.-O. Pålsson , A. Beeby , J. A. Mosely , H. M. Kaiser , D. Kaufmann , W.-Y. Wong , M. Blanchard-Desce , T. B. Marder , Chem. Eur. J. 2009, 15, 198–208. 19058267 10.1002/chem.200801719

[50] L. Ji , R. M. Edkins , L. J. Sewell , A. Beeby , A. S. Batsanov , K. Fucke , M. Drafz , J. A. K. Howard , O. Moutounet , F. Ibersiene , A. Boucekkine , E. Furet , Z. Liu , J.-F. Halet , C. Katan , T. B. Marder , Chem. Eur. J. 2014, 20, 13618–13635. 25168267 10.1002/chem.201402273

[51] P. Chen , A. S. Marshall , S. H. Chi , X. Yin , J. W. Perry , F. Jakle , Chem. Eur. J. 2015, 21, 18237–18247. 26514664 10.1002/chem.201502268

[52] S. Griesbeck , Z. Zhang , M. Gutmann , T. Lühmann , R. M. Edkins , G. Clermont , A. N. Lazar , M. Haehnel , K. Edkins , A. Eichhorn , M. Blanchard-Desce , L. Meinel , T. B. Marder , Chem. Eur. J. 2016, 22, 14701–14706. 27627995 10.1002/chem.201602639

[53] S. Griesbeck , E. Michail , F. Rauch , H. Ogasawara , C. Wang , Y. Sato , R. M. Edkins , Z. Zhang , M. Taki , C. Lambert , S. Yamaguchi , T. B. Marder , Chem. Eur. J. 2019, 25, 13164–13175. 31322301 10.1002/chem.201902461 PMC6857003

[54] S. Yamaguchi , S. Akiyama , K. Tamao , J. Am. Chem. Soc. 2001, 123, 11372–11375. 11707112 10.1021/ja015957w

[55] T. W. Hudnall , C.-W. Chiu , F. P. Gabbaï , Acc. Chem. Res. 2009, 42, 388–397. 19140747 10.1021/ar8001816

[56] K. Parab , K. Venkatasubbaiah , F. Jäkle , J. Am. Chem. Soc. 2006, 128, 12879–12885. 17002382 10.1021/ja063302v

[57] G. C. Welch , R. R. S. Juan , J. D. Masuda , D. W. Stephan , Science 2006, 314, 1124–1126. 17110572 10.1126/science.1134230

[58] G. C. Welch , D. W. Stephan , J. Am. Chem. Soc. 2007, 129, 1880–1881. 17260994 10.1021/ja067961j

[59] S. J. Geier , D. W. Stephan , J. Am. Chem. Soc. 2009, 131, 3476–3477. 19243095 10.1021/ja900572x

[60] D. W. Stephan , G. Erker , Angew. Chem. Int. Ed. 2010, 49, 46–76; 10.1002/anie.200903708 20025001

[61] D. W. Stephan , G. Erker , Chem. Sci. 2014, 5, 2625–2641.

[62] D. W. Stephan , J. Am. Chem. Soc. 2015, 137, 10018–10032. 26214241 10.1021/jacs.5b06794

[63] D. W. Stephan , G. Erker , Angew. Chem. Int. Ed. 2015, 54, 6400–6441; 10.1002/anie.201409800 25974714

[64] Y. Shirota , J. Mater. Chem. 2000, 10, 1–25.

[65] A. Wakamiya , K. Mori , S. Yamaguchi , Angew. Chem. Int. Ed. 2007, 46, 4273–4276; 10.1002/anie.200604935 17378005

[66] K. Suzuki , S. Kubo , K. Shizu , T. Fukushima , A. Wakamiya , Y. Murata , C. Adachi , H. Kaji , Angew. Chem. Int. Ed. 2015, 54, 15231–15235; 10.1002/anie.201508270 26563845

[67] J. J. Eisch , N. K. Hota , S. Kozima , J. Am. Chem. Soc. 1969, 91, 4575–4577.

[68] J. J. Eisch , J. E. Galle , S. Kozima , J. Am. Chem. Soc. 1986, 108, 379–385. 22175451 10.1021/ja00263a006

[69] S. Kim , K.-H. Song , S. O. Kang , J. Ko , Chem. Commun. 2004, 68–69. 10.1039/b312028g 14737336

[70] H. Braunschweig , I. Fernández , G. Frenking , T. Kupfer , Angew. Chem. Int. Ed. 2008, 47, 1951–1954; 10.1002/anie.200704771 18228230

[71] C. Fan , W. E. Piers , M. Parvez , Angew. Chem. Int. Ed. 2009, 48, 2955–2958; 10.1002/anie.200805865 19145617

[72] J. Köhler , S. Lindenmeier , I. Fischer , H. Braunschweig , T. Kupfer , D. Gamon , C.-W. Chiu , J. Raman Spectrosc. 2010, 41, 636–641.

[73] A. Steffen , R. M. Ward , W. D. Jones , T. B. Marder , Coord. Chem. Rev. 2010, 254, 1950–1976.

[74] H. Braunschweig , T. Kupfer , Chem. Commun. 2011, 47, 10903–10914. 10.1039/c1cc13071d 21735004

[75] H. Braunschweig , C.-W. Chiu , D. Gamon , M. Kaupp , I. Krummenacher , T. Kupfer , R. Müller , K. Radacki , Chem. Eur. J. 2012, 18, 11732–11746. 22886835 10.1002/chem.201201317

[76] T. Araki , A. Fukazawa , S. Yamaguchi , Angew. Chem. Int. Ed. 2012, 51, 5484–5487; 10.1002/anie.201201156 22499433

[77] H. Braunschweig , I. Krummenacher , J. Wahler , Adv. Organomet. Chem. 2013, 61, 1–53.

[78] H. Braunschweig , C. Hörl , L. Mailänder , K. Radacki , J. Wahler , Chem. Eur. J. 2014, 20, 9858–9861. 24964998 10.1002/chem.201403101

[79] Z. Zhang , R. M. Edkins , M. Haehnel , M. Wehner , A. Eichhorn , L. Mailander , M. Meier , J. Brand , F. Brede , K. Müller-Buschbaum , H. Braunschweig , T. B. Marder , Chem. Sci. 2015, 6, 5922–5927. 28791091 10.1039/c5sc02205c PMC5523080

[80] J. H. Barnard , S. Yruegas , K. Huang , C. D. Martin , Chem. Commun. 2016, 52, 9985–9991. 10.1039/c6cc04330e 27345619

[81] W. Zhang , B. Zhang , D. Yu , G. He , Sci. Bull. 2017, 62, 899–900. 10.1016/j.scib.2017.05.020 36659458

[82] M. Meier , L. Ji , J. Nitsch , I. Krummenacher , A. Deißenberger , D. Auerhammer , M. Schäfer , T. B. Marder , H. Braunschweig , Chem. Eur. J. 2019, 25, 4707–4712. 30786077 10.1002/chem.201805454

[83] H. C. Brown , V. H. Dodson , J. Am. Chem. Soc. 1957, 79, 2302–2306.

[84] P. J. Grisdale , J. L. R. Williams , M. E. Glogowski , B. E. Babb , J. Org. Chem. 1971, 36, 544–549.

[85] H. Braunschweig , V. Dyakonov , J. O. C. Jimenez-Halla , K. Kraft , I. Krummenacher , K. Radacki , A. Sperlich , J. Wahler , Angew. Chem. Int. Ed. 2012, 51, 2977–2980; 10.1002/anie.201108632 22334371

[86] S. Yamaguchi , T. Shirasaka , S. Akiyama , K. Tamao , J. Am. Chem. Soc. 2002, 124, 8816–8817. 12137533 10.1021/ja026689k

[87] A. Wakamiya , K. Mishima , K. Ekawa , S. Yamaguchi , Chem. Commun. 2008, 579–581. 10.1039/b716107g 18209795

[88] M. F. Smith , S. J. Cassidy , I. A. Adams , M. Vasiliu , D. L. Gerlach , D. A. Dixon , P. A. Rupar , Organometallics 2016, 35, 3182–3191.

[89] S. J. Cassidy , I. Brettell-Adams , L. E. McNamara , M. F. Smith , M. Bautista , H. Cao , M. Vasiliu , D. L. Gerlach , F. Qu , N. I. Hammer , D. A. Dixon , P. A. Rupar , Organometallics 2018, 37, 3732–3741.

[90] S. Yruegas , J. J. Martinez , C. D. Martin , Chem. Commun. 2018, 54, 6808–6811. 10.1039/C8CC01529E 29547215

[91] K. R. Bluer , L. E. Laperriere , A. Pujol , S. Yruegas , V. A. K. Adiraju , C. D. Martin , Organometallics 2018, 37, 2917–2927.

[92] S. Yruegas , J. H. Barnard , K. Al-Furaiji , J. L. Dutton , D. J. D. Wilson , C. D. Martin , Organometallics 2018, 37, 1515–1518.

[93] T. A. Bartholome , K. R. Bluer , C. D. Martin , Dalton Trans. 2019, 48, 6319–6322. 30942222 10.1039/c9dt01032g

[94] L. E. Laperriere , S. Yruegas , C. D. Martin , Tetrahedron 2019, 75, 937–943.

[95] P. A. Chase , P. E. Romero , W. E. Piers , M. Parvez , B. O. Patrick , Can. J. Chem. 2005, 83, 2098–2105.

[96] P. A. Chase , W. E. Piers , B. O. Patrick , J. Am. Chem. Soc. 2000, 122, 12911–12912.

[97] I. A. Adams , P. A. Rupar , Macromol. Rapid Commun. 2015, 36, 1336–1340. 25945461 10.1002/marc.201500107

[98] T. Shinji , A. Mitsuhiro , O. Michinori , T. Fumio , Bull. Chem. Soc. Jpn. 2000, 73, 2357–2362.

[99] J. Wang , Y. Wang , T. Taniguchi , S. Yamaguchi , S. Irle , J. Phys. Chem. A 2012, 116, 1151–1158. 22208822 10.1021/jp209264j

[100] Z. Zhang , R. M. Edkins , J. Nitsch , K. Fucke , A. Steffen , L. E. Longobardi , D. W. Stephan , C. Lambert , T. B. Marder , Chem. Sci. 2015, 6, 308–321. 28966759 10.1039/c4sc02410a PMC5586071

[101] X. Yin , F. Guo , R. A. Lalancette , F. Jäkle , Macromolecules 2016, 49, 537–546.

[102] R. J. Blagg , E. J. Lawrence , K. Resner , V. S. Oganesyan , T. J. Herrington , A. E. Ashley , G. G. Wildgoose , Dalton Trans. 2016, 45, 6023–6031. 26215924 10.1039/c5dt01918d

[103] M. Mantina , A. C. Chamberlin , R. Valero , C. J. Cramer , D. G. Truhlar , J. Phys. Chem. A 2009, 113, 5806–5812. 19382751 10.1021/jp8111556 PMC3658832

[104] C. J. Berger , G. He , C. Merten , R. McDonald , M. J. Ferguson , E. Rivard , Inorg. Chem. 2014, 53, 1475–1486. 24428809 10.1021/ic402408t

[105] W. Yang , K. E. Krantz , L. A. Freeman , D. A. Dickie , A. Molino , A. Kaur , D. J. D. Wilson , R. J. Gilliard, Jr. , Chem. Eur. J. 2019, 25, 12512–12516. 31334883 10.1002/chem.201903348

[106] T. Ishiyama , J. Takagi , K. Ishida , N. Miyaura , N. R. Anastasi , J. F. Hartwig , J. Am. Chem. Soc. 2002, 124, 390–391. 11792205 10.1021/ja0173019

[107] I. A. I. Mkhalid , J. H. Barnard , T. B. Marder , J. M. Murphy , J. F. Hartwig , Chem. Rev. 2010, 110, 890–931. 20028025 10.1021/cr900206p

[108] K. Schickedanz , T. Trageser , M. Bolte , H.-W. Lerner , M. Wagner , Chem. Commun. 2015, 51, 15808–15810. 10.1039/c5cc07208e 26366478

[109] K. Schickedanz , J. Radtke , M. Bolte , H.-W. Lerner , M. Wagner , J. Am. Chem. Soc. 2017, 139, 2842–2851. 28125773 10.1021/jacs.7b00268

[110] S. Konishi , T. Iwai , M. Sawamura , Organometallics 2018, 37, 1876–1883.

[111] M. W. Drover , K. Nagata , J. C. Peters , Chem. Commun. 2018, 54, 7916–7919. 10.1039/c8cc04321c PMC6095131 29951661

[112] G. A. Molander , J. Org. Chem. 2015, 80, 7837–7848. 26150178 10.1021/acs.joc.5b00981

[113] A. J. J. Lennox , G. C. Lloyd-Jones , Chem. Soc. Rev. 2014, 43, 412–443. 24091429 10.1039/c3cs60197h

[114] S. M. Cornet , K. B. Dillon , C. D. Entwistle , M. A. Fox , A. E. Goeta , H. P. Goodwin , T. B. Marder , A. L. Thompson , Dalton Trans. 2003, 4395–4405.

[115] R. J. Blagg , T. R. Simmons , G. R. Hatton , J. M. Courtney , E. L. Bennett , E. J. Lawrence , G. G. Wildgoose , Dalton Trans. 2016, 45, 6032–6043. 26541517 10.1039/c5dt03854e

[116] H. Jacobsen , H. Berke , S. Döring , G. Kehr , G. Erker , R. Fröhlich , O. Meyer , Organometallics 1999, 18, 1724–1735.

[117] H. Braunschweig , I. Krummenacher , Electrochemical Behavior and Redox Chemistry of Boroles, in Organic Redox Systems (Ed.: T. Nishinaga), Wiley, New York, 2015, pp. 503–522.

[118] P. Bissinger , H. Braunschweig , A. Damme , C. Hörl , I. Krummenacher , T. Kupfer , Angew. Chem. Int. Ed. 2015, 54, 359–362; 10.1002/anie.201409513 25389108

[119] A. Iida , S. Yamaguchi , J. Am. Chem. Soc. 2011, 133, 6952–6955. 21504199 10.1021/ja2019977

[120] J. M. Farrell , C. Mützel , D. Bialas , M. Rudolf , K. Menekse , A.-M. Krause , M. Stolte , F. Würthner , J. Am. Chem. Soc. 2019, 141, 9096–9104. 31117551 10.1021/jacs.9b04675

[121] S. A. Cummings , M. Iimura , C. J. Harlan , R. J. Kwaan , I. V. Trieu , J. R. Norton , B. M. Bridgewater , F. Jäkle , A. Sundararaman , M. Tilset , Organometallics 2006, 25, 1565–1568.

[122] N. G. Connelly , W. E. Geiger , Chem. Rev. 1996, 96, 877–910. 11848774 10.1021/cr940053x

[123] H. Braunschweig , F. Breher , C.-W. Chiu , D. Gamon , D. Nied , K. Radacki , Angew. Chem. Int. Ed. 2010, 49, 8975–8978; 10.1002/anie.201003611 20941718

[124] K. M. M. Josef , Lichtabsorption und Photochemie organischer Moleküle, VCH, Weinheim, 1989.

[125] A. G. Crawford , A. D. Dwyer , Z. Liu , A. Steffen , A. Beeby , L.-O. Pålsson , D. J. Tozer , T. B. Marder , J. Am. Chem. Soc. 2011, 133, 13349–13362. 21751803 10.1021/ja2006862

[126] T. M. Figueira-Duarte , K. Müllen , Chem. Rev. 2011, 111, 7260–7314. 21740071 10.1021/cr100428a

[127] M. K. Manna , S. Shokri , G. P. Wiederrecht , D. J. Gosztola , A. J.-L. Ayitou , Chem. Commun. 2018, 54, 5809–5818. 10.1039/c8cc01553h 29748666

[128] C.-C. Tsai , W.-C. Huang , H.-Y. Chih , Y.-C. Hsh , C.-W. Liao , C.-H. Lin , Y.-X. Kang , C.-H. Chang , Y. J. Chang , C.-W. Lu , Org. Electron. 2018, 63, 166–174.

[129] T. Hatakeyama , K. Shiren , K. Nakajima , S. Nomura , S. Nakatsuka , K. Kinoshita , J. Ni , Y. Ono , T. Ikuta , Adv. Mater. 2016, 28, 2777–2781. 26865384 10.1002/adma.201505491

[130] M.-Y. Zhang , Z.-Y. Li , B. Lu , Y. Wang , Y.-D. Ma , C.-H. Zhao , Org. Lett. 2018, 20, 6868–6871. 30359038 10.1021/acs.orglett.8b02995

[131] T.-L. Wu , M.-J. Huang , C.-C. Lin , P.-Y. Huang , T.-Y. Chou , R.-W. Chen-Cheng , H.-W. Lin , R.-S. Liu , C.-H. Cheng , Nat. Photonics 2018, 12, 235–240.

[132] I. S. Park , K. Matsuo , N. Aizawa , T. Yasuda , Adv. Funct. Mater. 2018, 28, 1802031.

[133] S.-Y. Li , Z.-B. Sun , C.-H. Zhao , ACS Omega 2018, 3, 12730–12736. 31457999 10.1021/acsomega.8b02004 PMC6645423

[134] D.-G. Chen , T.-C. Lin , C.-L. Chen , Y.-T. Chen , Y.-A. Chen , G.-H. Lee , P.-T. Chou , C.-W. Liao , P.-C. Chiu , C.-H. Chang , Y.-J. Lien , Y. Chi , ACS Appl. Mater. Interfaces 2018, 10, 12886–12896. 29582654 10.1021/acsami.8b00053

[135] C. Tu , W. Liang , ACS Omega 2017, 2, 3098–3109. 31457642 10.1021/acsomega.7b00514 PMC6641182

[136] Y.-J. Lien , T.-C. Lin , C.-C. Yang , Y.-C. Chiang , C.-H. Chang , S.-H. Liu , Y.-T. Chen , G.-H. Lee , P.-T. Chou , C.-W. Lu , Y. Chi , ACS Appl. Mater. Interfaces 2017, 9, 27090–27101. 28731681 10.1021/acsami.7b08258

[137] Y. H. Lee , S. Park , J. Oh , J. W. Shin , J. Jung , S. Yoo , M. H. Lee , ACS Appl. Mater. Interfaces 2017, 9, 24035–24042. 28653832 10.1021/acsami.7b05615

[138] Y. Kitamoto , T. Namikawa , T. Suzuki , Y. Miyata , H. Kita , T. Sato , S. Oi , Org. Electron. 2016, 34, 208–217.

[139] M. Numata , T. Yasuda , C. Adachi , Chem. Commun. 2015, 51, 9443–9446. 10.1039/c5cc00307e 25959457

[140] Y. Kitamoto , T. Namikawa , D. Ikemizu , Y. Miyata , T. Suzuki , H. Kita , T. Sato , S. Oi , J. Mater. Chem. C 2015, 3, 9122–9130.

[141] M. Stanoppi , A. Lorbach , Dalton Trans. 2018, 47, 10394–10398. 29737352 10.1039/c8dt01255e

[142] Y. Liu , C. Li , Z. Ren , S. Yan , M. R. Bryce , Nat. Rev. Mater. 2018, 3, 18020.

[143] F. B. Dias , T. J. Penfold , A. P. Monkman , Methods Appl. Fluores. 2017, 5, 012001. 10.1088/2050-6120/aa537e 28276340

[144] D. Reitzenstein , T. Quast , F. Kanal , M. Kullmann , S. Ruetzel , M. S. Hammer , C. Deibel , V. Dyakonov , T. Brixner , C. Lambert , Chem. Mater. 2010, 22, 6641–6655.

[145] D. Tsiplakides , D. Archonta , C. G. Vayenas , Top. Catal. 2007, 44, 469–479.

[146] M. J. G. Peach , P. Benfield , T. Helgaker , D. J. Tozer , J. Chem. Phys. 2008, 128, 044118. 18247941 10.1063/1.2831900

[147] T. Yanai , D. P. Tew , N. C. Handy , Chem. Phys. Lett. 2004, 393, 51–57.

