# Supplementary material for: Highly Stable, Readily Reducible, Fluorescent, Trifluoromethylated 9‐Borafluorenes
Source: Chemistry. 2020 Sep 21;26(56):12794–808. doi: 10.1002/chem.201905559 (PMC7589458; doi:10.1002/chem.201905559)
Supplement: Supplementary file 1 — Supplementary [file CHEM-26-12794-s001.pdf]

Chemistry–A European Journal

Supporting Information

Highly Stable, Readily Reducible, Fluorescent, Trifluoromethylated 9-Borafluorenes

Florian Rauch+, Sonja Fuchs+, Alexandra Friedrich, Daniel Sieh, Ivo Krummenacher, Holger Braunschweig, Maik Finze, and Todd B. Marder*[a]

Table of contents

General experimental details 3

Synthetic procedure 6

NMR-Data 16

Single-crystal X-ray diffraction 36

Electrochemistry 45

Photophysical properties 46

TD-DFT calculations 49

Theoretical calculation: Cartesian coordinate 54

General experimental details

Unless otherwise noted, the following conditions apply.

All syntheses were carried out using standard Schlenk and glovebox techniques under an argon atmosphere. The solvents used were dried using a solvent purification system (SPS) from Innovative Technology and were degassed and stored over molecular sieves under argon. Deuterated solvents (CD_2Cl_2, $CDCl_3$, C_6D_6, acetone-d_6, and DMSO-d_6) used for NMR spectroscopy were purchased from Cambridge Isotope Laboratories. C_6D_6 was dried over molecular sieves and stored under an argon atmosphere before use. Trimethylborate was purchased from Sigma Aldrich and distilled before use. *n*-Butyllithium (2.5 M solution in hexane) was purchased from Acros Organics and used as received. The compounds 1-bromo-bis-2,4-(trifluoromethyl)benzene and 3,5-bis-trifluoromethylaniline were purchased from Fluorochem and used as received. B_2pin_2 was kindly provided by AllyChem Co. Ltd. (Dalian, China). Tris-1,3,5-trifluoromethylbenzene was purchased from Fluorochem, distilled and degassed before use. Bis-1,3-trifluoromethylbenzene was purchased from ABCR, distilled and degassed before use. $CoCp_2$ was kindly provided by Prof. Dr. Udo Radius. $[Ir(COD)(OMe)]_2$ was prepared according to a literature procedure.[1] All other starting materials were purchased from commercial sources and were used without further purification.

Column chromatography was performed with silica gel 60 (40-63 µ) (purchased from VWR), or alumina 90 active basic (purchased from Merck), and automated flash chromatography was performed on silica gel (Biotage SNAP catridge KP-Sil 10 g or KP-Sil 100 g), obtained from Biotage, using a Biotage® Isolera Four Flash system. Solvents were generally removed using a rotary evaporator *in vacuo* at a maximum temperature of 55 °C.

NMR spectra were recorded on a Bruker Avance 200 (operating at ^{1}H: 199.9 MHz, ^{11}B{^{1}H}: 64.1 MHz, ^{19}F{^{1}H}: 188.1 MHz) or a Bruker Avance 500 FT NMR spectrometer (operating at ^{1}H: 500 MHz, ^{11}B{^{1}H}: 160 MHz, ^{13}C{^{1}H}: 126 MHz, ^{19}F{^{1}H}: 470.6 MHz. Chemical shifts (δ) are given in ppm and are referenced to external $BF_3{\cdot}Et_2O$ (^{11}B{^{1}H}) and $CFCl_3$ (^{19}F{^{1}H}). ^{1}H NMR spectra were referenced via residual proton resonances of $CDCl_3$ (7.26 ppm), CD_2Cl_2 (5.32 ppm), acetone-d_6 (2.05 ppm), and C_6D_6 (7.16 ppm).[2] ^{13}C{^{1}H} spectra were referenced to $CDCl_3$ (77.16 ppm), CD_2Cl_2(53.84 ppm), acetone-d_6 (29.84 ppm)and C_6D_6 (128.06 ppm).[2] GCMS analyses were performed on an Agilent Technologies GCMS system (GC 7890A, EI-MS 5975C). HRMS were recorded using a Thermo Scientific Exactive Plus Orbitrap MS system with either an Atmospheric Sample Analysis Probe (ASAP) or by Electro-Spray Ionization (ESI).

Single-crystal X-ray diffraction: Crystals suitable for single-crystal X-ray diffraction were selected, coated in perfluoropolyether oil, and mounted on MiTeGen sample holders. Diffraction data were collected on Bruker X8 Apex II 4-circle diffractometers with CCD area detectors using Mo-Kα radiation monochromated by graphite (**^{F}Mes^{F}Bf•MeCN**, **^{F}XylBF$_3$K**, compound **1**) or multi-layer focusing mirrors (**^{F}Mes^{F}Bf**, **^{F}Xyl^{F}Bf**, ***p*-NMe$_2$-^{F}Xyl^{F}Bf**, **[^{F}Mes^{F}Bf]$^{\cdot-}$**, ***p*-NMe$_2$-^{F}Xyl^{F}Bf•HF**, compounds **D** and **2**). The crystals were cooled using an Oxford Cryostreams low-temperature device. Data were collected at 100 K. The images were processed and corrected for Lorentz-polarization effects and absorption as implemented in the Bruker

software packages. The structures were solved using the intrinsic phasing method (SHELXT)[3] and Fourier expansion technique. All non-hydrogen atoms were refined in anisotropic approximation, with hydrogen atoms ‘riding’ in idealized positions, by full-matrix least squares against F^2 of all data, using SHELXL[4] software and the SHELXLE graphical user interface.[5] The crystal of **^{F}Mes^{F}Bf•MeCN** was a pseudo-merohedral twin with domains rotated by 179.9° around real axis [100]. The twin fraction was refined to 13.5%. Diamond[6] software was used for graphical representation. Other structural information was extracted using OLEX2[7] software. Crystal data and experimental details are listed in Table S1; full structural information has been deposited with Cambridge Crystallographic Data Centre. CCDC-1940986 (**^{F}Mes^{F}Bf**), 1940987 (**^{F}Xyl^{F}Bf**), 1940988 (***p*-NMe$_2$-^{F}Xyl^{F}Bf**), 1940989 (**^{F}Mes^{F}Bf•MeCN**), 1940990 (**[^{F}Mes^{F}Bf]$^{\bullet-}$**), 1940991 (***p*-NMe$_2$-^{F}Xyl^{F}Bf•HF**), 1940992 (**D**), 1949706 (**^{F}XylBF$_3$K**), 1949707 (**1**), and 1949708 (**2**).

Photophysical measurements: All measurements were performed in standard quartz cuvettes (1 cm x 1 cm cross-section). UV–visible absorption spectra were recorded using an Agilent 8453 diode array UV-visible spectrophotometer.

Extinction coefficients of **^{F}Mes^{F}Bf**, **^{F}Xyl^{F}Bf** and ***p*-NMe$_2$-^{F}Xyl^{F}Bf** were calculated from 6 independently prepared samples in hexane.

Emission spectra were recorded using an Edinburgh Instruments FLSP920 spectrometer equipped with a double monochromator for both excitation and emission, operating in right-angle geometry mode, and all spectra were fully corrected for the spectral response of the instrument. All solutions used for photophysical measurements had a concentration lower than 2×10^{-5} M to minimize inner filter effects during fluorescence measurements.

Fluorescence quantum yields were measured using a calibrated integrating sphere (inner diameter: 150 mm) from Edinburgh Instruments combined with the FLSP920 spectrometer described above. For solution-state and solid-state measurements, the longest-wavelength absorption maximum of the compound in the respective solvent was chosen as the excitation wavelength.

Fluorescence lifetimes were recorded using the time-correlated single-photon counting (TCSPC) method using the same FLSP920 spectrometer described above. Solutions were excited with a picosecond pulsed diode laser at an emission maximum of 376.6 nm. The full width at half maximum (FWHM) of the laser pulses were ca. 72 ps, while the instrument response function (IRF) had a FWHM of ca. 1.0 ns, measured from the scatter of a Ludox solution at the excitation wavelength. Decays were recorded to at least 10000 counts in the peak channel with a record length of at least 1000 channels. The band pass of the monochromator was adjusted to give a signal count rate of <10 kHz. Iterative reconvolution of the IRF with one decay function and non-linear least-squares analysis were used to analyze the data. The quality of the fit was judged by the calculated value of the reduced χ^2 and visual inspection of the weighted residuals.

Electrochemical measurements: All cyclic voltammetry experiments were conducted in an argon-filled glovebox using a Gamry Instruments Reference 600 potentiostat. A standard three-electrode cell configuration was employed using a platinum disk working electrode, a platinum wire counter electrode,

and a silver wire reference electrode separated by a Vycor frit, serving as the reference electrode. The redox potentials are referenced to the ferrocene/ferrocenium ([Fc/Fc$^+$]) redox couple by using decamethylferrocene ([Cp*$_2$Fe]; $E_{1/2}$ = –0.532 V in CH_2Cl_2) as an internal standard. Tetra-*n*-butylammonium hexafluorophosphate ([nBu$_4$N][PF$_6$]) was employed as the supporting electrolyte. Compensation for resistive losses (*iR* drop) was employed for all measurements

EPR measurements at X-band (9.37 GHz) were carried out at room temperature using a Bruker ELEXSYS E580 CW EPR spectrometer. CW EPR spectra were measured using 1 mW microwave power and 0.5 G field modulation at 100 kHz, with a conversion time of 20 ms. The spectral simulations were performed using MATLAB 8.6 and the EasySpin 5.2.11 toolbox.[8]

Theoretical Studies: All calculations (DFT and TD-DFT) were carried out with the Gaussian 09 (9.E.01)[9] program package and were performed on a parallel cluster system. GaussView (6.0.16) and multiwfn[10] were used to visualize the results, to measure calculated structural parameters, and to plot orbital surfaces (isovalue: ± 0.030 [e a_0^{-3}]$^{1/2}$). The ground-state geometries were optimized using the B3LYP functional[11] in combination with the 6-31+G(d) basis set.[12, 13] The D3 dispersion correction of Grimme and coworkers was used.[14] The polarizable continuum model (PCM) was used to include solvent effects for the ground state structures. The ultrafine integration grid and symmetry constraints were used for all molecules. Frequency calculation were performed on the optimized structures to confirm them to be local minima showing no negative (imaginary) frequencies. Based on these optimized structures, the lowest-energy vertical transitions (gas-phase and solvent correction using the polarizable continuum model) were calculated (singlets, 25 states) by TD-DFT, using the Coulomb attenuated functional CAM-B3LYP[15] as well as B3LYP. The CAM-B3LYP has been shown to more accurately describe ICT systems in comparison to B3LYP.[16] The optimized ground-state geometries were used as starting coordinates for TD-DFT geometry optimizations. The S_1 states of **^{F}Mes^{F}Bf** and **^{F}Xyl^{F}Bf** were optimized using eight excited states with the B3LYP functional in combination with the 6-31+g(d) basis set and D3 dispersion correction and no symmetry constraints. The S_1 state of ***p*-NMe$_2$-^{F}Xyl^{F}Bf** was optimized using eight states with the CAM-B3LYP functional in combination with the 6-31+G(d) basis set and D3 dispersion correction and no symmetry constraints.

Synthetic procedures

Synthesis of 2-(2-bromo-3,5-bis(trifluoromethyl)phenyl)-4,4,5,5-tetramethyl-1,3,2-dioxaborolane

B_2pin_2 (3.9 g, 15.4 mmol), 4,4'-di-tert-butyl-2,2'-bipyridine (247 mg, 0.9 mmol) and $[Ir(COD)OMe]_2$ (305 mg, 0.46 mmol) were dissolved in hexane and stirred for 10 min, then 1-bromo-2,4-bis(trifluoromethyl)benzene (4.5 g, 2.6 mL, 15.4 mmol) was added dropwise. The suspension was stirred for 12 h and afterwards filtered through a silica plug with hexane as the eluent. The solvent was removed under reduced pressure to obtain 2-(2-bromo-3,5-bis(trifluoromethyl)phenyl)-4,4,5,5-tetramethyl-1,3,2-dioxaborolane (4.54 g, 10.8 mmol) as a white solid in 70% yield.

^{1}H NMR (500 MHz, 298 K, $CDCl_3$): δ(ppm) = 7.93 (m, 2 H), 1.41 (s, 12 H)

^{11}B{^{1}H} NMR (64.1 MHz, 298 K, $CDCl_3$): δ(ppm) = 30.5

^{19}F{^{1}H} NMR (188.1 MHz, 298 K, $CDCl_3$): δ(ppm) = –62.8 (s, 3 F), –63.0 (s, 3 F)

^{13}C{^{1}H} NMR (126 MHz, 298 K, $CDCl_3$): δ(ppm) = 138.0 (br), 135.4 (q, $^3J_{CF}$ = 4 Hz,), 131.5 (q, $^2J_{CF}$ = 32 Hz), 129.4 (q, $^2J_{CF}$ = 34 Hz), 129.4 (br), 126.5 (m), 123.4 (q, $^1J_{CF}$ = 271 Hz), 122.6 (q, $^1J_{CF}$ = 274 Hz), 85.4 (s, 2C, C_q), 24.9 (s, 4C, CH_3)

HRMS (APCI$^+$): *m/z* calculated for $[C_{14}H_{15}B_1Br_1F_6O_2]^+$ 419.0247, found 419.0245 $[M]^+$ ($|\Delta|$ = 0.4 ppm)

Synthesis of 2,2'-dibromo-3,3',5,5'-tetrakis(trifluoromethyl)-1,1'-biphenyl (2)

The compound 2-(2-bromo-3,5-bis(trifluoromethyl)phenyl)-4,4,5,5-tetramethyl-1,3,2-dioxaborolane (0.875 mg, 2.08 mmol) and $Cu(OAc)_2$ (0.075 g, 0.41 mmol) were dissolved in 200 mL of methanol open to air and the reaction was stirred for 12 h at room temperature. All volatiles were removed under reduced pressure and the resulting solid was dissolved in hexane and filtered through a silica plug using hexane as the eluent. The volatiles were removed under reduced pressure and compound **2** was obtained as a white solid (0.49 g, 0.83 mmol) in 80% yield.

^{1}H NMR (500 MHz, 298 K, C_6D_6): δ(ppm) = 7.79 (d, 2 H, ^4J = 2 Hz), 6.85 (d, 2 H, ^4J = 2 Hz)

^{13}C{^{1}H} NMR (126 MHz, 298 K, C_6D_6): δ(ppm) = 144.2 (2C), 132.6 (q, $^2J_{CF}$ = 32 Hz, 2C), 130.5 (q, $^3J_{CF}$ = 1 Hz, 2C), 130.5 (q, $^2J_{CF}$ = 34 Hz, 2C), 126.2 (m, 2C), 125.3 (m, 2C), 123.3, (q, $^1J_{CF}$ = 273 Hz, 2C), 122.7 (q, $^1J_{CF}$ = 274 Hz, 2C)

^{19}F{^{1}H} NMR (470.6 MHz, 298 K, C_6D_6): δ(ppm) = –62.5 (s, 6 F), –62.9 (s, 6 F)

HRMS (APCI$^+$): *m/z* calculated for $[C_{16}H_4Br_1F_{12}]^+$ 504.9279, found 504.9276 $[M]^+$ ($|\Delta|$ = 0.1 ppm)

Synthesis of 9-(2,4,6-tris(trifluoromethyl)phenyl)-2,4,6,8-tetrakis(trifluoromethyl)-dibenzoborole (^{F}Mes^{F}Bf)

In a Schlenk tube compound **2** (1.00 g, 1.71 mmol) was dissolved in 30 mL of diethylether. A 2.5 M *n*BuLi solution in hexane (0.22 g, 1.4 mL, 3.51 mmol) was added dropwise at –78 °C and the mixture was stirred for 45 min. Meanwhile, ^{F}Mes-BF_3K (0.66 g, 1.71 mmol) and LiBr (0.15 g, 1.71 mmol) were suspended in 10 mL of diethylether and stirred for 45 min. Then, the suspension was added dropwise to the lithiated compound **(2)** and the reaction was slowly warmed to room temperature overnight. After addition of a few drops of *iso*-propanol, the solvent was removed under reduced pressure. The resulting solid was purified via sublimation (180 °C, 5 x 10^{-2} mbar) and washed with hexane to obtain compound **^{F}Mes^{F}Bf** (0.25 g, 0.35 mmol) as a bright green solid in 20% yield. Single crystals of compound **^{F}Mes^{F}Bf** were obtained by recrystallization from hexane.

^{1}H NMR (500 MHz, 298 K, C_6D_6): δ(ppm) = 7.93 (s, 2 H), 7.43 (s, 2 H), 7.11 (s, 2 H)

^{11}B{^{1}H} NMR (160 MHz, 298 K, C_6D_6): δ(ppm) = 64.1 (br)

^{13}C{^{1}H} NMR (126 MHz, 298 K, $CDCl_3$): δ(ppm) =152.9 (2C), 139.9 (br, 2C), 137.7 (q, $^2J_{CF}$ = 33 Hz, 2C), 136.3 (q, $^2J_{CF}$ = 33 Hz, 2C),134.0 (q, $^2J_{CF}$ = 33 Hz, 2C), 133.4 (q, $^2J_{CF}$ = 35 Hz), 124.1 (q, $^1J_{CF}$ = 274 Hz2C), 122.7 (q, $^1J_{CF}$ = 278 Hz2C), 122.6 (q, $^1J_{CF}$ = 273 Hz), 122.4 (q, $^1J_{CF}$ = 274 Hz, 2C) 124.2 (m, 2C), 120.4 (m, 2C)

^{19}F{^{1}H} NMR (471 MHz, 298 K, C_6D_6): δ(ppm) = –58.4 (sept, 6 F, J_{FF} = 4 Hz), –59.6 (sept, 6 F, J_{FF} = 4 Hz), –62.0 (s, 3 F), –63.5 (s, 6 F)

^{19}F{^{1}H} NMR (471 MHz, 298 K, $CDCl_3$): δ(ppm) = –58.6 (sept, 6 F, J_{FF} = 4 Hz), –59.6 (sept, 6 F, J_{FF} = 4 Hz), –63.1 (s, 3 F), –64.0 (s, 6 F)

HRMS (APCI$^-$): *m/z* calculated for $[C_{25}H_6B_1F_{21}]^-$ 716.0233, found 716.0245 $[M]^-$ ($|\Delta|$ = 1.6 ppm)

Synthesis of 9-(2,6-bis(trifluoromethyl)phenyl)-2,4,6,8-tetrakis(trifluoromethyl)-dibenzoborole (^{F}Xyl^{F}Bf)

In a Schlenk tube, compound **2** (1.00 g, 1.71 mmol) was dissolved in 30 mL of diethylether. A 2.5 M *n*BuLi solution in hexane (0.22 g, 1.40 mL, 3.51 mmol) was added dropwise at –78 °C and the reaction mixture was stirred for 30 min. Meanwhile, ^{F}Xyl-BF_3K (0.55 g, 1.71 mmol) and LiBr (1.71 mmol, 0.15 g) were suspended in 10 mL of diethylether and stirred for 30 min. Then the suspension was added dropwise to the lithiated compound **(2)** and the reaction was slowly warmed to room temperature overnight. After addition of a few drops of *iso*-propanol, the solvent was removed under reduced pressure. The resulting solid was purified via sublimation (170 °C, 5 x 10^{-2} mbar) and recrystallization from hexane to obtain **^{F}Xyl^{F}Bf** (91 mg, 139 mmol) as a bright green solid in 8% yield. Single crystals of compound **^{F}Xyl^{F}Bf** for X-ray diffraction were obtained by crystallization from dichloromethane**.**

^{1}H NMR (500 MHz, 298 K, C_6D_6): δ(ppm) = 7.49 (s, 2 H), 7.31 (d, 2 H, J_{HH} = 8 Hz), 7.16 (s under solvent signal, 2 H) 6.85 (t, 2 H, J_{HH} = 8 Hz)

^{11}B{^{1}H} NMR (160 MHz, 298 K, C_6D_6): δ(ppm) = 63.2 (br)

^{13}C{^{1}H} NMR (126 MHz, 298 K, C_6D_6): δ(ppm) = 152.7 (2C), 140.5 (2C), 136.8 (q, $^{2}J_{CF}$ = 33 Hz, 2C), 136.0 (q, $^{2}J_{CF}$ = 33 Hz, 2C), 132.7 (q, $^{2}J_{CF}$ = 33 Hz, 2C), 130.4, 129.1 (m, 2C), 125.6 (q, $^{1}J_{CF}$ = 274 Hz, 2C), 123.9 (m, 2C), 123.2 (q, $^{1}J_{CF}$ = 273 Hz, 2C), 122.8 (q, $^{1}J_{CF}$ = 274 Hz, 2C), 120.5 (m, 2C)

^{19}F{^{1}H} NMR (471 MHz, 298 K, C_6D_6): δ(ppm) = –58.2 (sept, 6 F, J_{FF} = 3 Hz) –59.6 (sept, 6 F, J_{FF} = 4 Hz), –63.4 (s, 6 F)

HRMS (APCI^{-}): *m/z* calculated for $[C_{24}H_7B_1F_{18}]^-$ 648.0348, found 648.0336 $[M]^-$ ($|\Delta|$ = 1.8 ppm)

Synthesis of 9-(4-(dimethylamino)-2,6-bis(trifluoromethyl)phenyl)-2,4,6,8-tetrakis(trifluoromethyl)-dibenzoborole (*p*-NMe$_2$-^{F}Xyl^{F}Bf)

Compound **2** (1.00 g, 1.71 mmol) was dissolved in 30 mL of diethylether and a 2.5 M *n*BuLi solution in hexane (0.22 g, 1.40 mL, 3.51 mmol) was added dropwise at –78 °C and the reaction mixture was stirred for 45 min. Meanwhile, FXyl-BF$_3$K (0.61 g, 1.71 mmol) and LiBr (0.15 g, 1.71 mmol) were suspended in 10 mL of diethylether and stirred. Then, the suspension was added dropwise to the lithiated compound **(6)** and the reaction was slowly warmed to room temperature overnight. After addition of a few drops *iso*-propanol, the solvent was removed under reduced pressure. The resulting solid was purified via sub-limation (200 °C, 5 x 10^{-2} mbar) and washed with hexane to obtain ***p*-NMe$_2$-^{F}Xyl^{F}Bf** (25 mg, 36.2 mmol) as a red solid in 2% yield. Crystals of compound ***p*-NMe$_2$-^{F}Xyl^{F}Bf** suitable for X-ray diffraction were ob-tained by crystallization from hexane at –30 °C**.**

^{1}H NMR (500 MHz, 298 K, C_6D_6): δ(ppm) = 7.57 (s, 2 H), 7.22 (s, 2 H), 6.95 (s, 2 H), 2.10 (s, 6 H)

^{11}B{^{1}H} NMR (160 MHz, 298 K, C_6D_6): δ(ppm) = 64.7 (br)

^{13}C{^{19}F} NMR (126 MHz, 298 K, C_6D_6): δ(ppm) = 152.7 (d, $^2J_{CH}$ = 3 Hz, 2C) 150.9 (br, 2C), 141.3 (br, 2C), 136.4 (2C), 136.1 (2C), 133.9 (2C), 129.0 (2C), 126.12 (m, 2C), 123.8 (dd, $^1J_{CH}$ = 167 Hz, $^2J_{CH}$ = 6 Hz, 2C), 123.3 (t, $^2J_{CH}$ = 4 Hz), 123.1 (d, $^2J_{CH}$ = 4 Hz, 2C), 120.4 (dd, $^1J_{CH}$ = 164 Hz, $^2J_{CH}$ = 6 Hz, 2C), 117.93 (br, 2C), 111.2 (dd, $^1J_{CH}$ = 160 Hz, $^2J_{CH}$ = 5 Hz, 2C), 38.87 (m, 2C)

^{19}F{^{1}H} NMR (471 MHz, 298 K, C_6D_6): δ(ppm) = –58.1 (sept, 6 F, J_{FF} = 3 Hz), –59.5 (sept, 6 F, J_{FF} = 4 Hz), –63.4 (s, 6 F)

HRMS (APCI$^-$): *m/z* calculated for $[C_{26}H_{12}B_1F_{18}N]^-$ 691.0781, found 691.0792 $[M]^-$ ($|\Delta|$ = 1.6 ppm)

Synthesis of potassium (2,4,6-tris(trifluoromethyl)phenyl)trifluoroborate

2,4,6-tris(trifluoromethyl)benzene (4.50 g, 2.97 mL, 16.0 mmol) was dissolved in 70 mL of diethylether and then a 2.5 M *n*BuLi solution in hexane (1.12 g, 7.02 mL, 17.6 mmol) was added dropwise at −78 °C and the mixture was allowed to warm slowly to room temperature over 8 h. Afterwards, the reaction was cooled to −78 °C, $B(OMe)_3$ (4.97 g, 5.39 mL, 47.9 mmol) was added dropwise and the reaction was slowly warmed to room temperature overnight. Then, the suspension was filtered, and the solvent was removed under reduced pressure. The resulting solid was dissolved in 200 mL of methanol, while KHF_2 (3.31 g, 42.4 mmol) was dissolved in 25 mL of H_2O. The aqueous solution was added dropwise, and the reaction mixture was stirred overnight. The solvents were removed under reduced pressure and the remaining solid was suspended in hexane and filtered. The product was extracted with acetone and filtered. The solvent was removed from the filtrate under reduced pressure to give potassium (2,4,6-tris(trifluoromethyl)phenyl)trifluoroborate (4.9 g, 12.6 mmol) as a white solid in 89% yield.

^{1}H NMR (500 MHz, 298 K, acetone-d_6): δ(ppm) = 8.05 (s, 2 H)

^{11}B{^{1}H} NMR (160 MHz, 298 K, acetone-d_6): δ(ppm) = 1.9 (q, J_{BF} = 44 Hz)

^{13}C{^{1}H} NMR (126 MHz, 298 K, acetone-d_6): δ(ppm) =155.1 (br), 137.7 (q, $^{2}J_{CF}$ = 32 Hz, 2C), 127.7 (q, J_{CF} = 33 Hz), 125.0 (br, 2C), 124.5 (q, J_{CF} = 274 Hz, 2C) 123.7 (q, J_{CF} = 271 Hz).

^{19}F{^{1}H} NMR (471 MHz, 298 K, acetone-d_6): δ(ppm) = −57.9 (q, 6 F J_{FF} = 14 Hz), −63.5 (s, 3 F), −136.4 (m, 3 F)

HRMS (ESI$^-$): *m/z* calculated for $[C_9H_2B_1F_{12}]^-$: 349.0063, found 349.0062 $[M]^-$ ($|\Delta|$ = 0.3 ppm)

Synthesis of 2-iodo-1,3-bis(trifluoromethyl)benzene

The herein reported synthesis is an optimization of the conditions reported by Schlosser and co-workers.[17] The synthetic conditions were optimized in terms of batch size, yield as well as cost efficiency.

A solution of MeLi in Et_2O (1.6 M solution in Et_2O, 80 mmol, 1.0 eq.) was added dropwise to a solution of KOtBu (17.95 g, 160 mmol, 2.0 eq.) in THF (250 mL) at –78 °C. After addition, the solution was stirred at –78°C for 30 min. Then 1,3-bis(trifluoromethyl)benzene (12.3 mL, 80.0 mmol, 1.0 eq.) was added dropwise and the dark purple reaction was stirred for 3 h at –78°C. Then iodine (20.3 g, 80 mmol, 1.0 eq.) was rapidly added as a solid. The reaction was stirred overnight and slowly warmed to ambient temperature. Afterwards the reaction was diluted with 200 mL pentane and filter over a silica plug (pentane/silica, 5 cm). All volatiles were removed under reduced pressure to give a brown oil. The raw product was sublimed (2 x 10-2 mbar, 30 to 80 °C) to give 2-iodo-1,3-bis(trifluoromethyl)benzene as a colorless solid (18.7 g, 63.6 mmol, 80%).

^{1}H NMR (200 MHz, 298 K, $CDCl_3$): δ(ppm) = 7.82 (d, ^3J = 8 Hz, 2H), 7.58 (t, ^3J = 8 Hz, 1H) ppm

^{19}F NMR (188.1 MHz, 298 K, $CDCl_3$): δ(ppm) = –61.9 ppm

Elem. Anal. Calc. (%) for $C_8H_3F_6I$: C 28.26, H 0.89; found: C 28.57, H 0.87

HRMS (APCI$^+$): *m/z* calculated for $[C_8H_3F_6I]^+$: 339.9178, found 339.9170 $[M]^+$ ($|\Delta|$ = 2.4 ppm)

The spectroscopic data match those previously reported.[17]

Synthesis of potassium (2,6-bis(trifluoromethyl)phenyl)trifluoroborate

The compound 2-iodo-1,3-bis(trifluoromethyl)benzene (3.05 g, 8.97 mmol) was dissolved in 50 mL of $MeO^{t}Bu$ (MTBE). A 2.5 M *n*BuLi solution in hexane (3.77 mL, 9.42 mmol) was added dropwise at –78 °C and the reaction mixture was stirred for 45 min. Then, $B(OMe)_3$ (2.02 mL, 17.94 mmol) was added dropwise to the lithiated species and the reaction was slowly warmed to room temperature overnight. Afterwards, the suspension was filtered and the solvent was removed from the filtrate under reduced pressure to give dimethyl(2,6-bis(trifluoromethyl)phenyl)boronate. The remaining solid was dissolved in 80 mL of MeOH, while KHF_2 (2.3 g, 30 mmol) was dissolved in 10 mL of H_2O. The aqueous solution was added dropwise, and the methanol-water solution was stirred for 12 h. The solvents were removed from the mixture under reduced pressure and the resulting solid was suspended in hexane and filtered. The product was extracted with acetone and filtered. The solvent was removed from the filtrate under reduced pressure to give potassium (2,6-bis(trifluoromethyl)phenyl)trifluoroborate (2.8 g, 8.8 mmol) as a white solid in 89% yield.

^{1}H NMR (200 MHz, 298 K, DMSO-d_6): δ(ppm) = 7.75 (d, 2 H, J_{HH} = 8 Hz), 7.43 (t, 1 H, J_{HH} = 8 Hz)

^{11}B{^{1}H} NMR (64 MHz, 298 K, DMSO-d_6): δ(ppm) = 1.9 (q, J_{BF} = 45 Hz)

^{19}F{^{1}H} NMR (188 MHz, 298 K, DMSO-d_6): δ(ppm) = –55.6 (q, 6 F, J_{FF} = 14 Hz), −133.2 (m, 3 F)

^{13}C{^{1}H} NMR (126 MHz, 298 K, DMSO-d_6): δ(ppm) = 134.5 (q, 2 C, $^{2}J_{CF}$ = 31 Hz), 128.6 (q, 2 C, $^{3}J_{CF}$ = 6 Hz), 126.0 (1 C), 125.6 (q, 2 C, $^{1}J_{CF}$ = 275 Hz).

HRMS (ESI$^-$): *m/z* calculated for $[C_8H_3B_1F_9]^-$: 281.0186, found 281.0189 $[M]^-$ ($|\Delta|$ = 3.55 ppm)

Synthesis of N,N-dimethyl-3,5-bis(trifluoromethyl)aniline

In a 250 mL flask with a solvent reservoir (typically used for column chromatography) and a condenser attached to it, 3,5-bis(trifluoromethyl)aniline (10.0 g, 43.6 mmol) was stirred at 0 °C. Formic acid (16.5 mL, 390 mmol) was added slowly and the reaction mixture was warmed to room temperature. Then, formaldehyde (30.1 mL, 437 mmol) was added, and the reaction mixture was heated to reflux. During the reaction the crude product sublimes as a white solid in the reservoir. The sublimed product was dissolved in 100 mL of Et_2O and washed with 3 x 50 mL of water. The organic phase was dried over magnesium sulfate and filtered. After removal of all volatiles under reduced presseuer, N,N-dimethyl-3,5-bis(trifluoromethyl)aniline (9.1 g, 35.4 mmol) was obtained as a white solid in 81% yield.

The analytical data match those previously reported.[18]

Experimental setup:

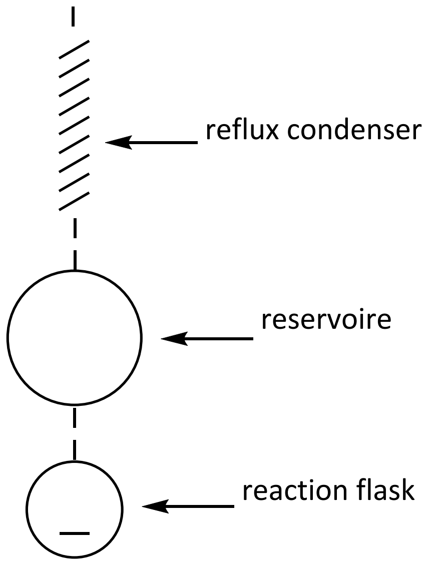

Synthesis of 4-bromo-N,N-dimethyl-3,5-bis(trifluoromethyl)aniline

N,N-dimethyl-3,5-bis(trifluoromethyl)aniline (3.0 g, 11.7 mmol) was dissolved in 150 mL of acetonitrile and N-bromosuccinimide (2.28 g, 12.8 mmol) was added over 10 min. The yellow solution was stirred for 20 min, then an excess of saturated sodium thiosulfate solution was added to obtain a colorless solution. Afterwards, 200 mL of Et_2O were added and the solution was washed with 3 x 50 mL of brine. The organic phase was dried over sodium sulfate and filtered. All volatiles were removed under reduced pressure, and the resulting solid was recrystallized from hexane and dried *in vacuo*. The product 4-bromo-*N,N*-dimethyl-3,5-bis(trifluoromethyl)aniline was obtained as white solid (3.1 g, 9.22 mmol) in 79% yield.

^{1}H NMR (500 MHz, 298 K, $CDCl_3$): δ(ppm) = 7.11 (s, 2 H), 3.06 (s, 6 H)

^{13}C{^{1}H} NMR (126 MHz, 298 K, $CDCl_3$): δ(ppm) = 148.7 (s), 132.9 (q, J_{CF} = 30 Hz), 123.1 (q, J_{CF} = 274 Hz), 113.6 (q, J_{CF} = 6 Hz), 101.7 (m), 40.3 (s)

^{19}F{^{1}H} NMR (471 MHz, 298 K, $CDCl_3$): δ(ppm) = –62.1 (s, 6 F)

HRMS (ASAP$^+$): *m/z* calculated for $[C_{10}H_9Br_1F_6]^+$: 335.9817, found 335.9810 $[M]^+$ ($|\Delta|$ = 3.42 ppm)

Synthesis of potassium (4-(dimethylamino)-2,6-bis(trifluoromethyl)phenyl)trifluoroborate)

The compound 4-bromo-*N,N*-dimethyl-3,5-bis(trifluoromethyl)aniline (2.5 g, 7.4 mmol) was dissolved in 50 mL of diethylether, a 2.5 M *n*BuLi solution in hexane (3.12 mL, 7.81 mmol) was added dropwise at –78 °C, and the mixture was stirred for 45 min. Then, $B(OMe)_3$ (1.7 mL, 15 mmol) was added dropwise and the reaction was slowly warmed to room temperatures overnight. Afterwards, the suspension was filtered, and the solvent was removed from the filtrate under reduced pressure to give dimethyl(4-(dimethylamino)-2,6-bis(trifluoromethyl)phenyl)boronate. This was dissolved in 80 mL of methanol, while KHF_2 (1.84 mg, 23.6 mmol) was dissolved in 10 mL of H_2O. The aqueous solution was added dropwise, and the methanol-water solution was stirred 12 h. The solvents were removed under reduced pressure and the resulting solid was washed with hexane and filtered and then extracted with acetone and filtered. The solvent was removed under reduced pressure to give potassium (4-(dimethylamino)-2,6-bis(trifluoromethyl)phenyl)trifluoroborate) (1.3 g, 3.6 mmol) as a white solid in 45% yield.

^{1}H NMR (500 MHz, 298 K, acetone-d_6): δ(ppm) = 7.11 (s, 2 H), 2.97 (s, 6 H)

^{11}B{^{1}H} NMR (160 MHz, 298 K, acetone-d_6): δ(ppm) = 2.5 (q, J_{BF} = 49 Hz)

^{13}C{^{1}H} NMR (126 MHz, 298 K, acetone-d_6): δ(ppm) = 149.0 (s), 136.9 (m), 126.6 (q, J_{CF} = 274 Hz), 113.2 (q, J_{CF} = 7 Hz), 40.4 (s)

^{19}F{^{1}H} NMR (471 MHz, 298 K, acetone-d_6): δ(ppm) = –57.3 (q, 6 F, J_{FF} = 14 Hz), –135.1 (m, 3 F)

HRMS (ESI$^-$): *m/z* calculated for $[C_{10}H_8B_1F_9N_1]^-$ 324.0612, found 324.0611 $[M]^-$ ($|\Delta|$ = 0.3 ppm)

NMR spectra

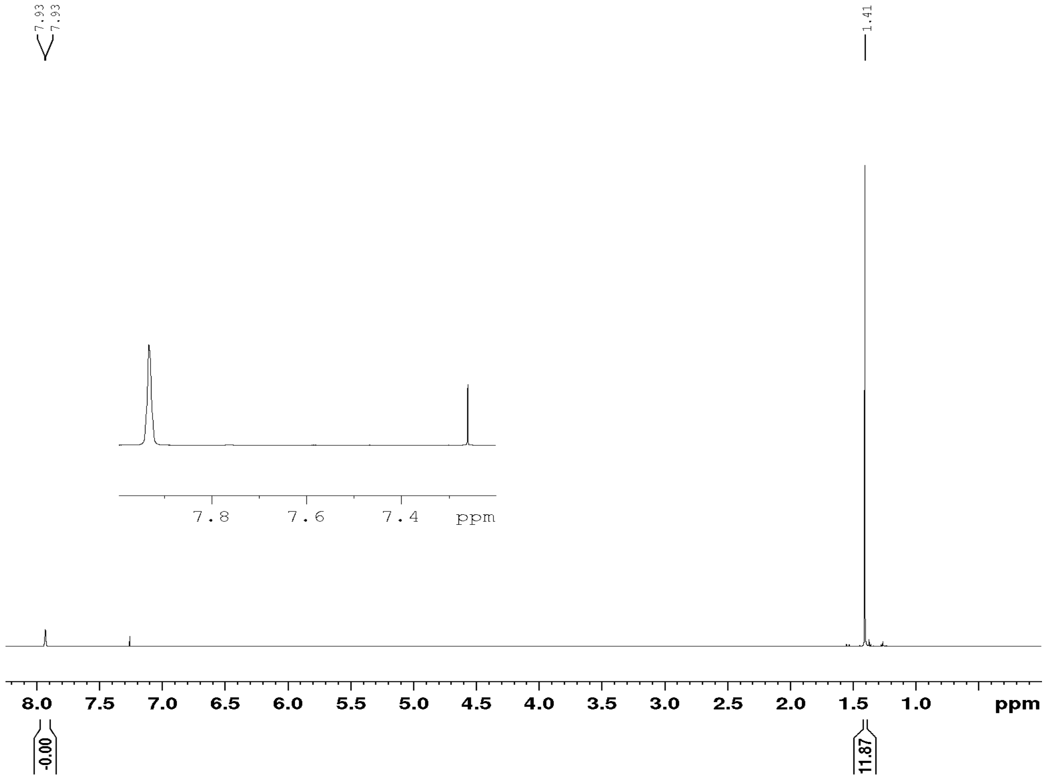

Figure S1: ^{1}H NMR spectrum (500 MHz, 298 K) of **2-(2-bromo-3,5-bis(trifluoromethyl)phenyl)-4,4,5,5-tetramethyl-1,3,2-dioxaborolane** recorded in $CDCl_3$.

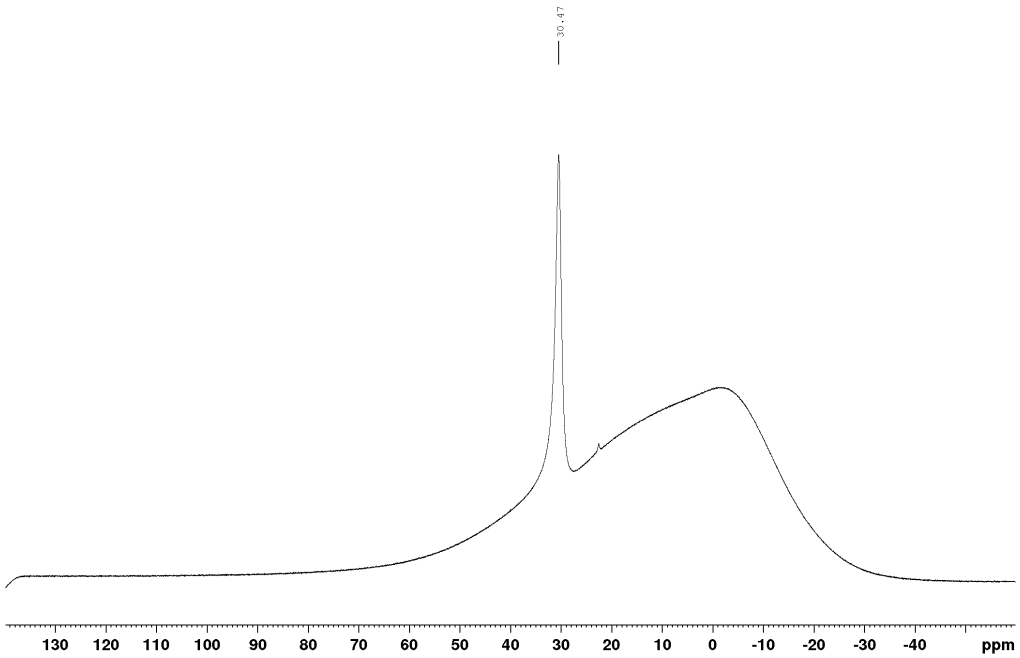

Figure S2: $^{11}B\{^{1}H\}$ NMR spectrum (160 MHz, 298 K) of **2-(2-bromo-3,5-bis(trifluoromethyl)phenyl)-4,4,5,5-tetramethyl-1,3,2-dioxaborolane** recorded in $CDCl_3$.

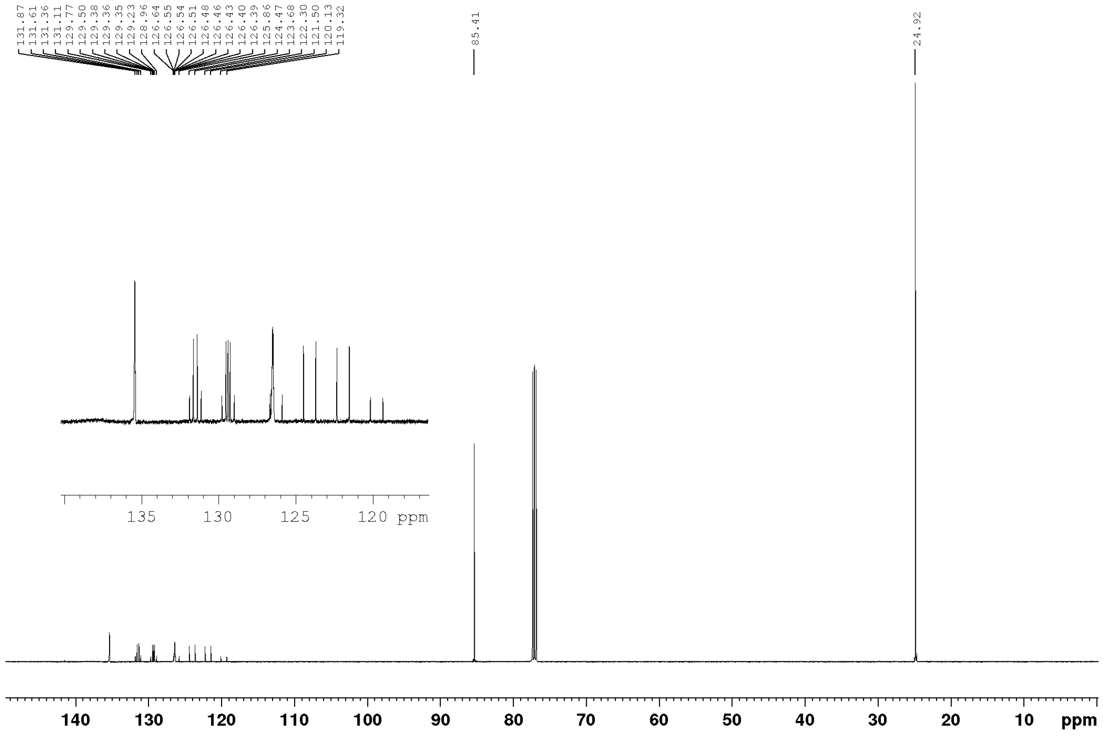

Figure S3: $^{13}C\{^{1}H\}$ NMR spectrum (126 MHz, 298 K) of **2-(2-bromo-3,5-bis(trifluoromethyl)phenyl)-4,4,5,5-tetramethyl-1,3,2-dioxaborolane** recorded in $CDCl_3$.

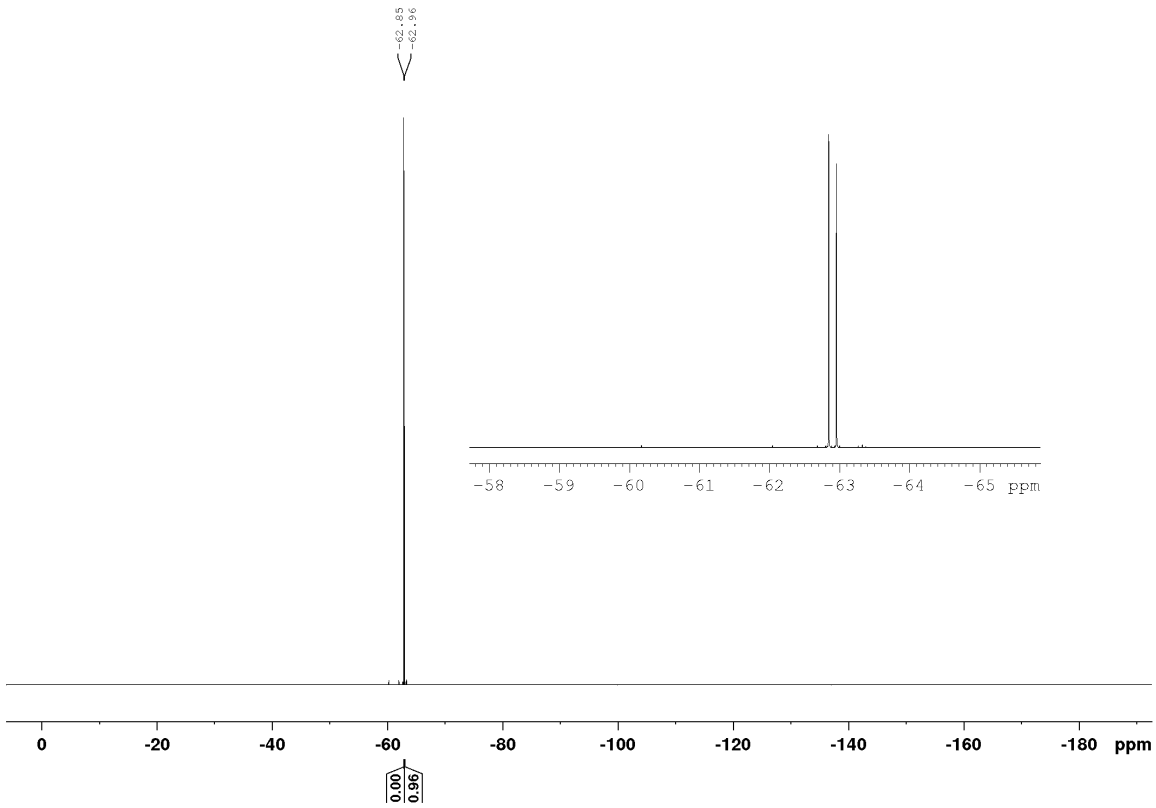

Figure S4: $^{19}F\{^{1}H\}$ NMR spectrum (471 MHz, 298 K) of **2-(2-bromo-3,5-bis(trifluoromethyl)phenyl)-4,4,5,5-tetramethyl-1,3,2-dioxaborolane** recorded in $CDCl_3$.

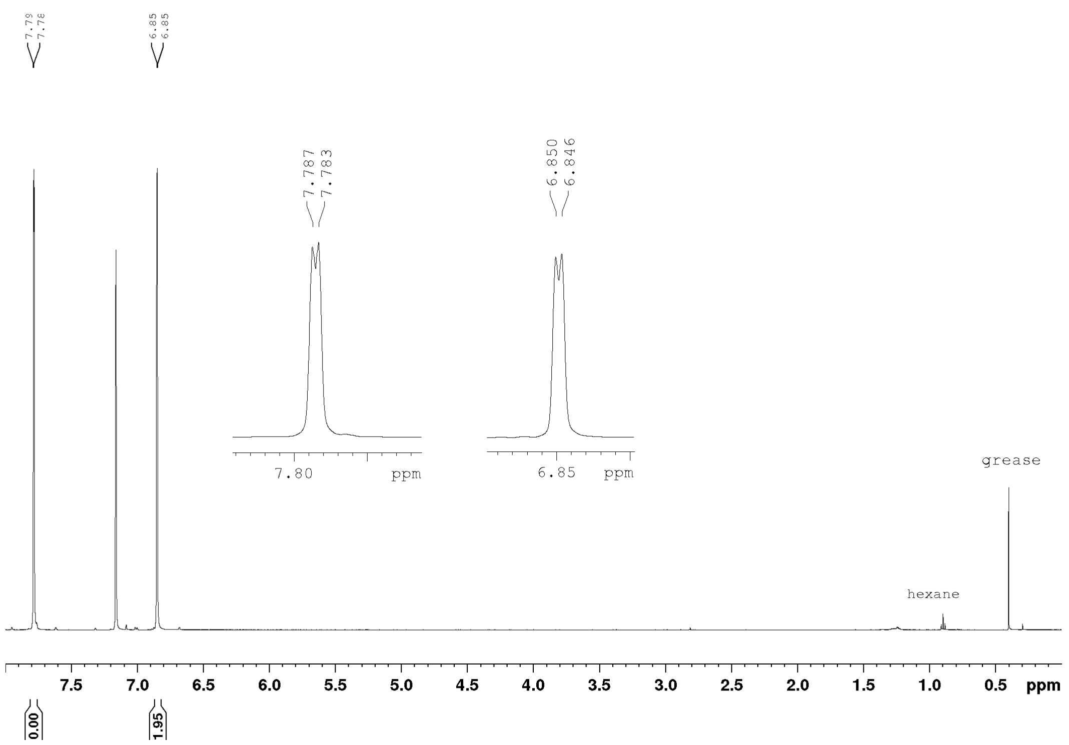

Figure S5: ^{1}H NMR spectrum (500 MHz, 298 K) of **2,2'-dibromo-3,3',5,5'-tetrakis(trifluoromethyl)-1,1'-biphenyl** (**2**) recorded in C_6D_6.

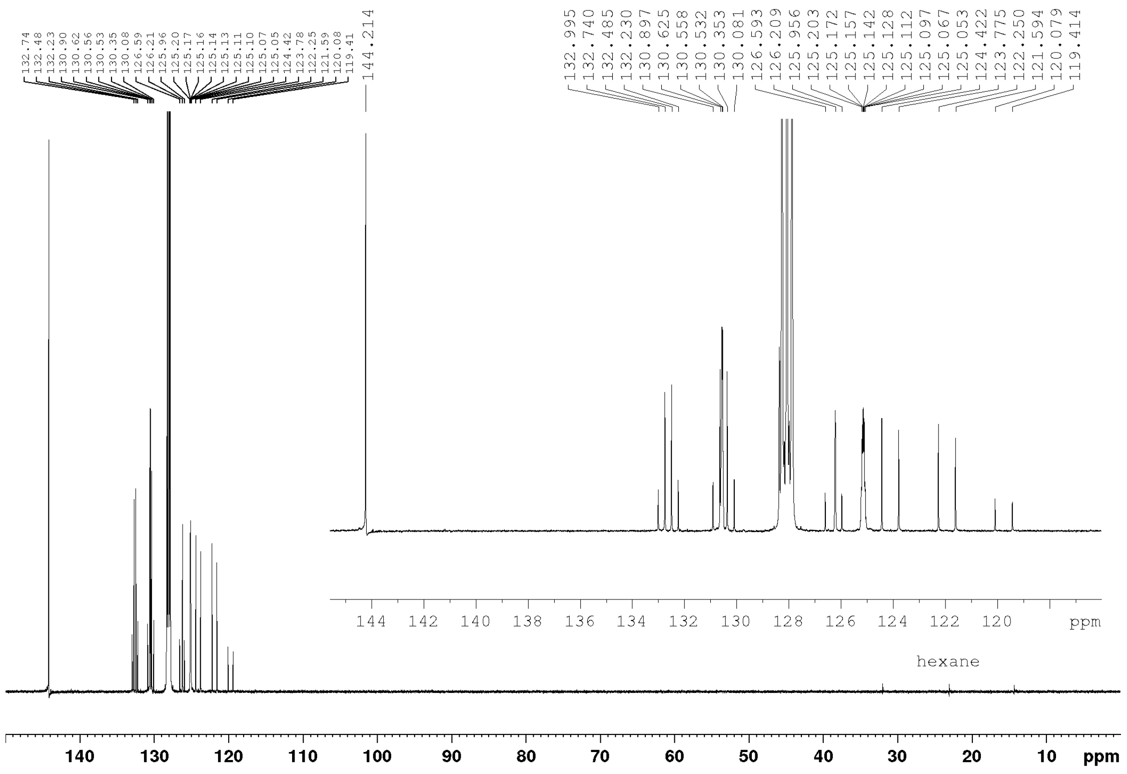

Figure S6: $^{13}C\{^{1}H\}$ NMR spectrum (126 MHz, 298 K) **of 2,2'-dibromo-3,3',5,5'-tetrakis(trifluoromethyl)-1,1'-biphenyl** (**2**) recorded in C_6D_6.

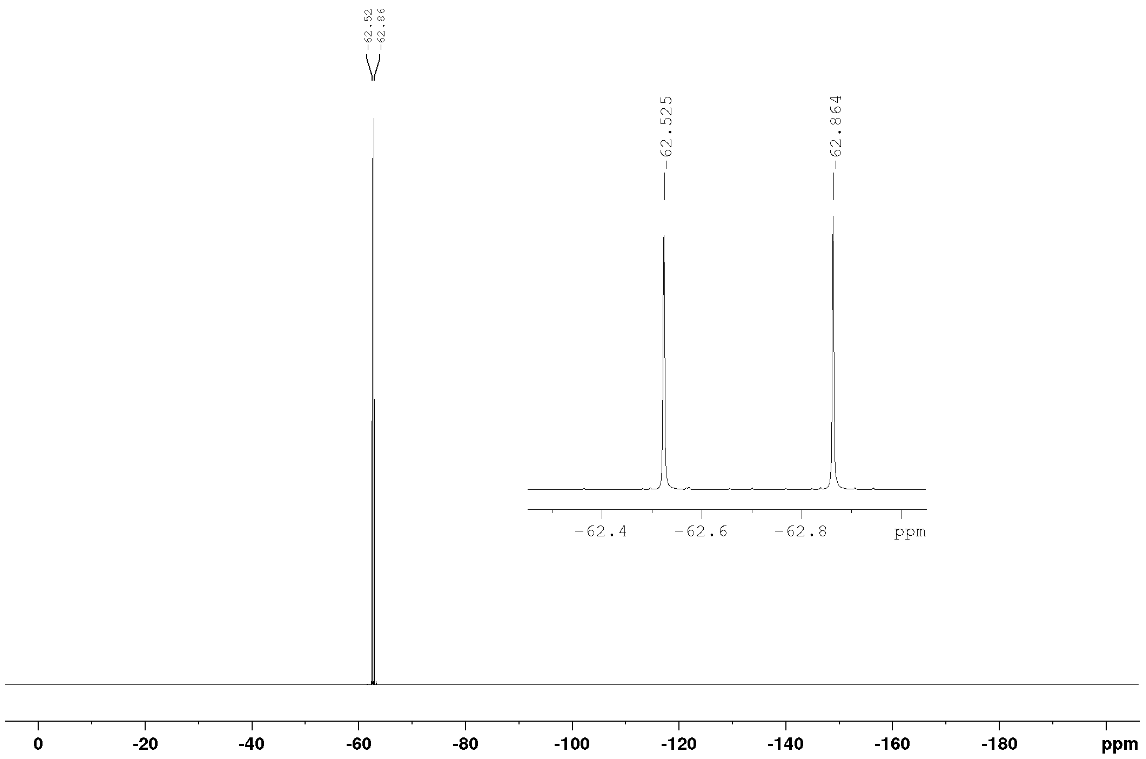

Figure S7: $^{19}F\{^{1}H\}$ NMR spectrum (471 MHz, 298 K) of **2,2'-dibromo-3,3',5,5'-tetrakis(trifluoromethyl)-1,1'-biphenyl** (**2**) recorded in C_6D_6.

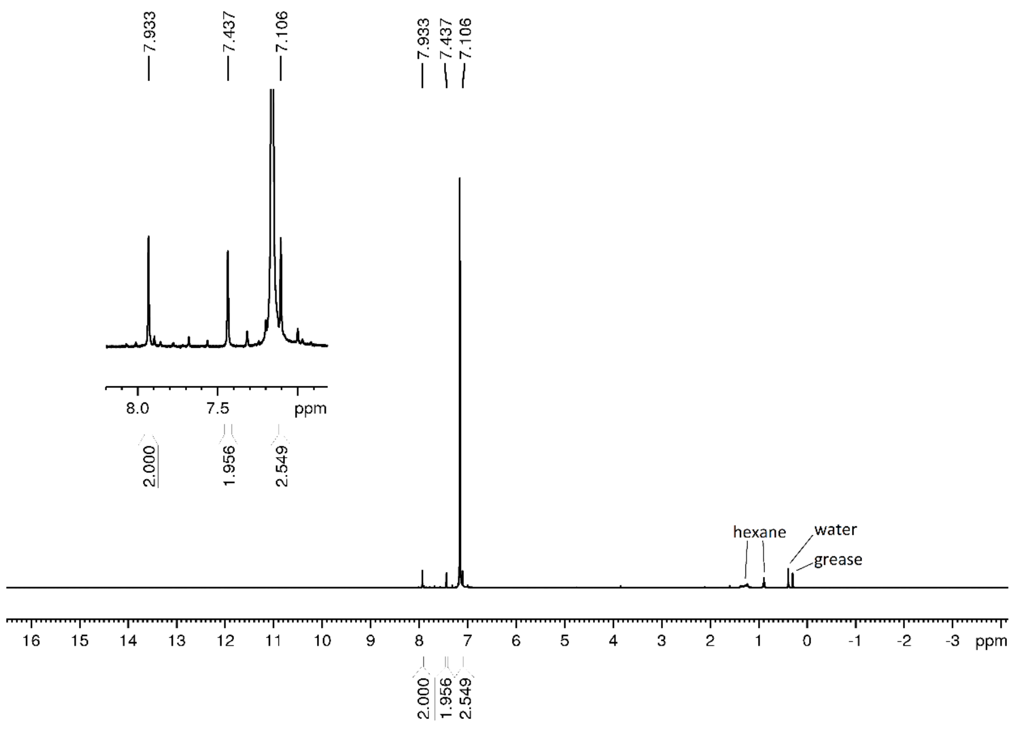

Figure S8: ^{1}H NMR spectrum (500 MHz, 298 K) of **^{F}Mes^{F}Bf** recorded in C_6D_6.

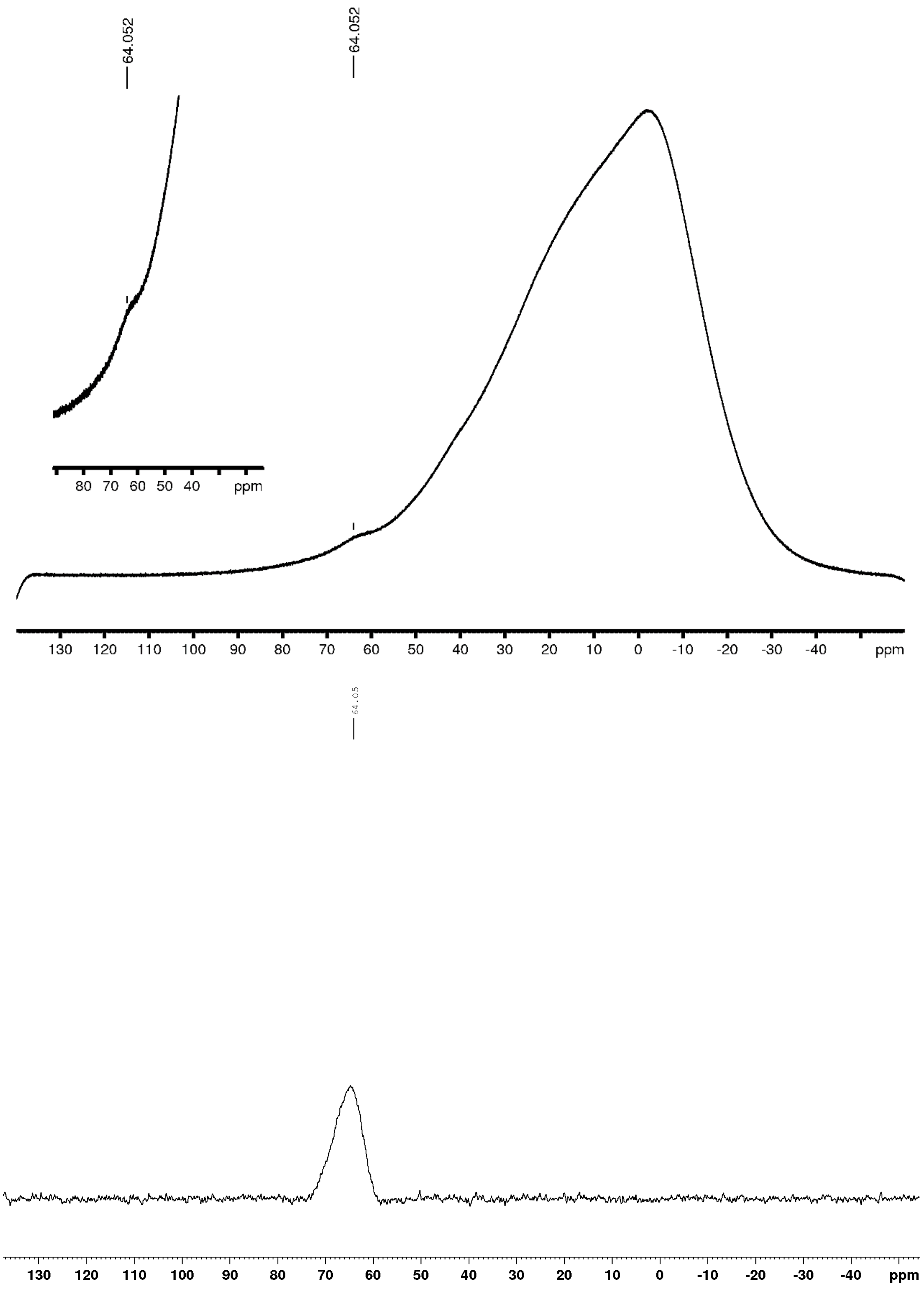

Figure S9: $^{11}B\{^{1}H\}$ NMR spectrum (160 MHz, 298 K) of **^{F}Mes^{F}Bf** recorded in C_6D_6 (top) and baseline corrected spectrum (bottom).

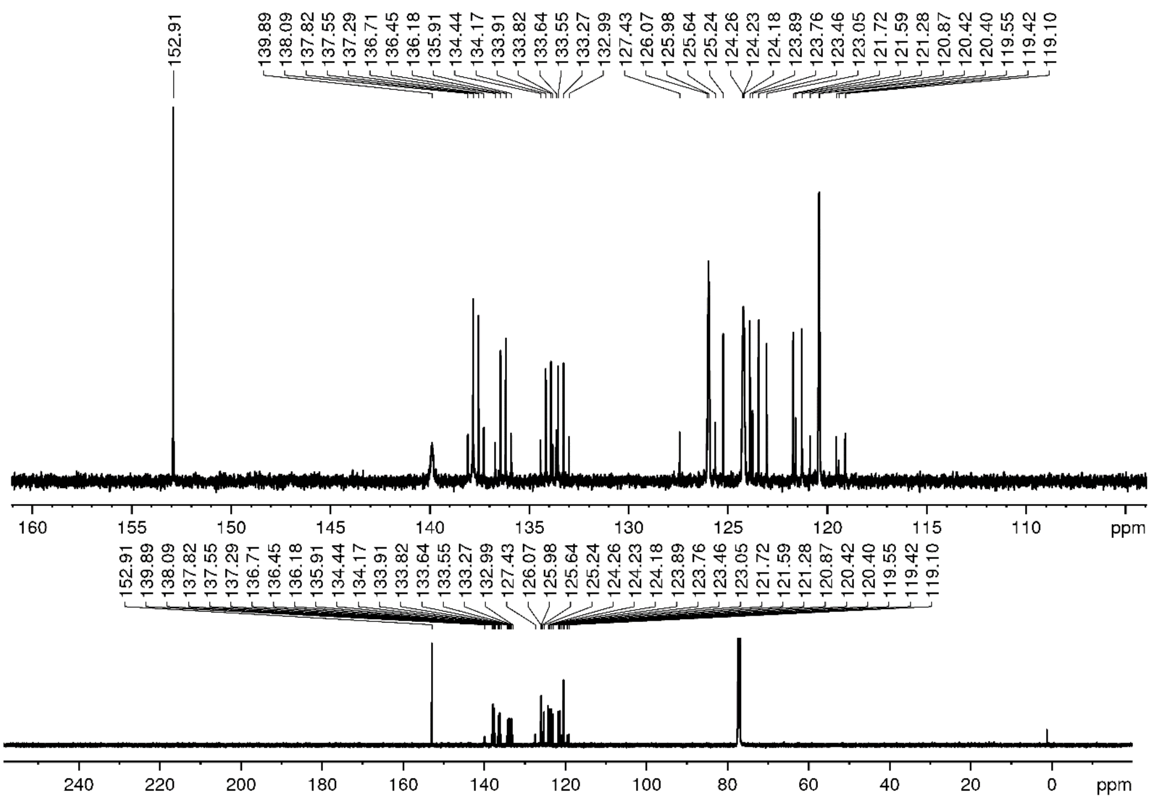

Figure S10: $^{13}C\{^{1}H\}$ NMR spectrum (126 MHz, 298 K) of **^{F}Mes^{F}Bf** recorded in $CDCl_3$.

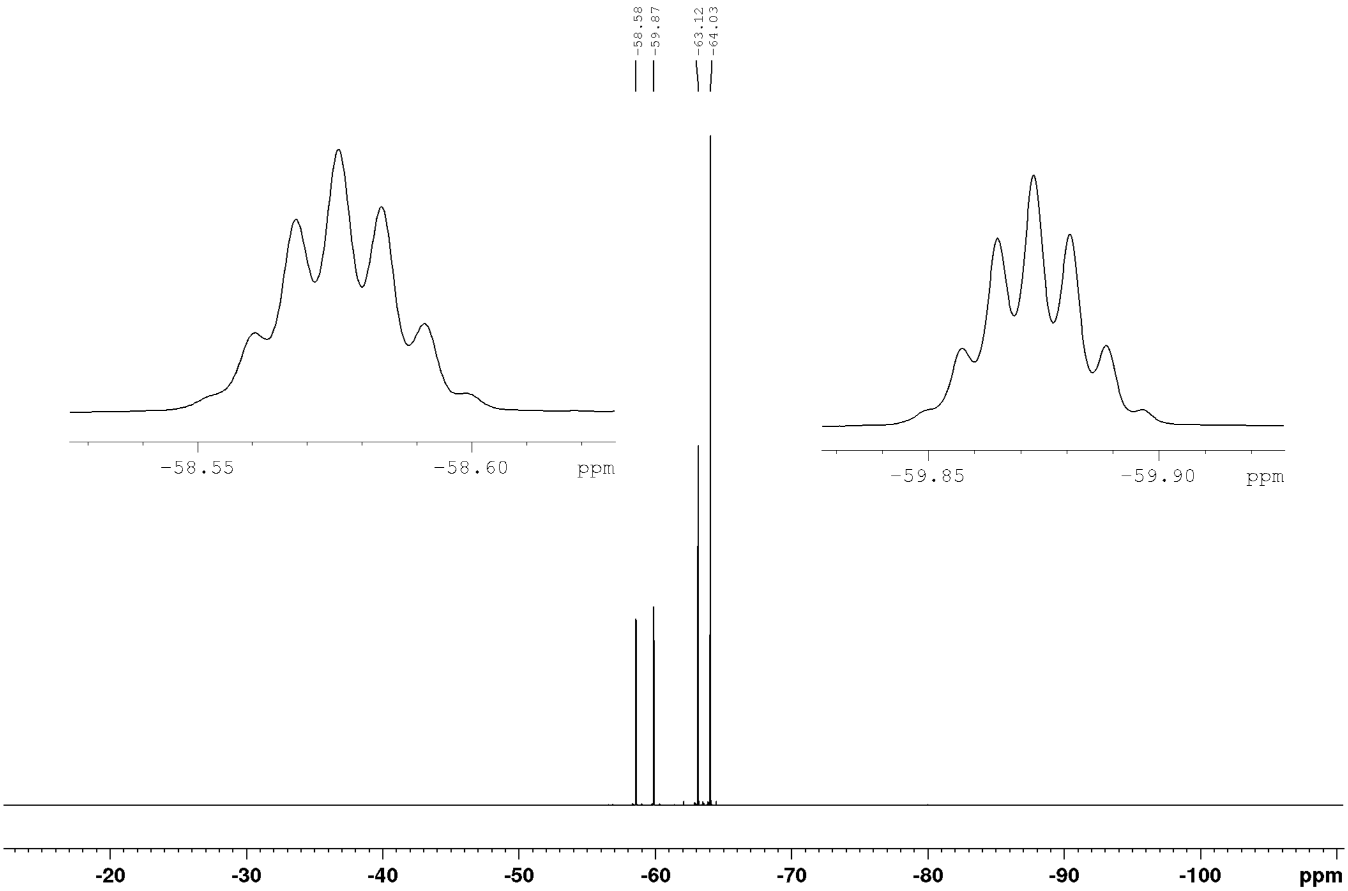

Figure S11: $^{19}F\{^{1}H\}$ NMR spectrum (471 MHz, 298 K) of **^{F}Mes^{F}Bf** recorded in $CDCl_3$.

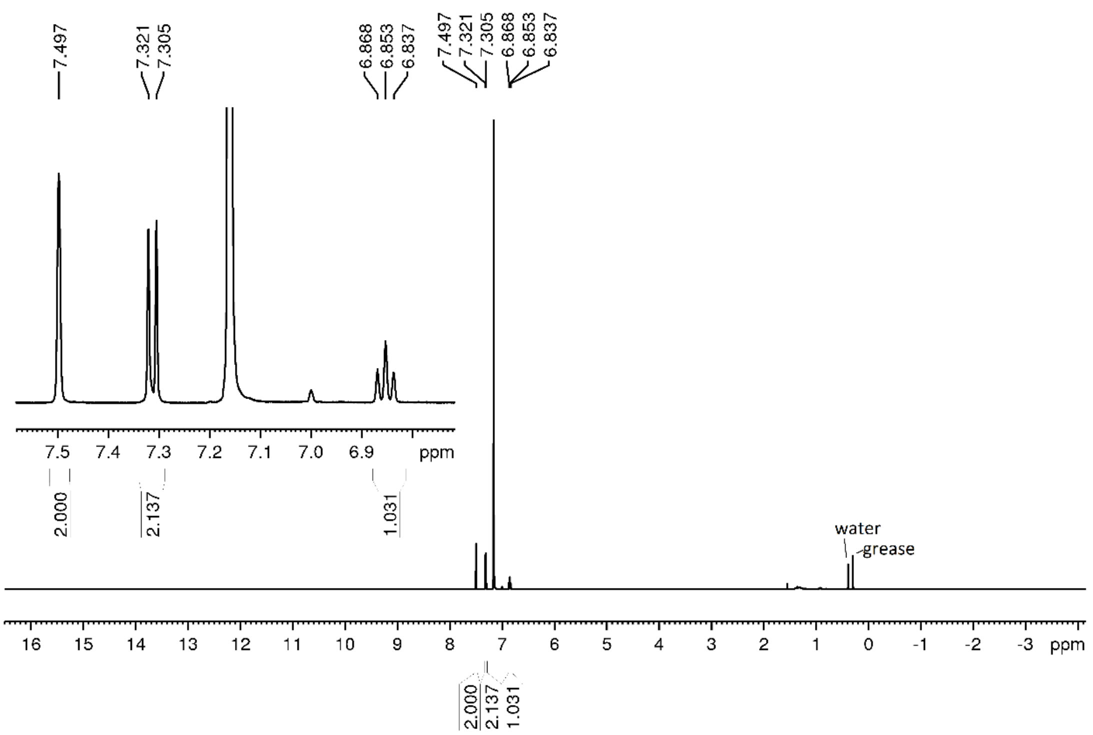

Figure S12: ^{1}H NMR spectrum (500 MHz, 298 K) of **^{F}Xyl^{F}Bf** recorded in C_6D_6.

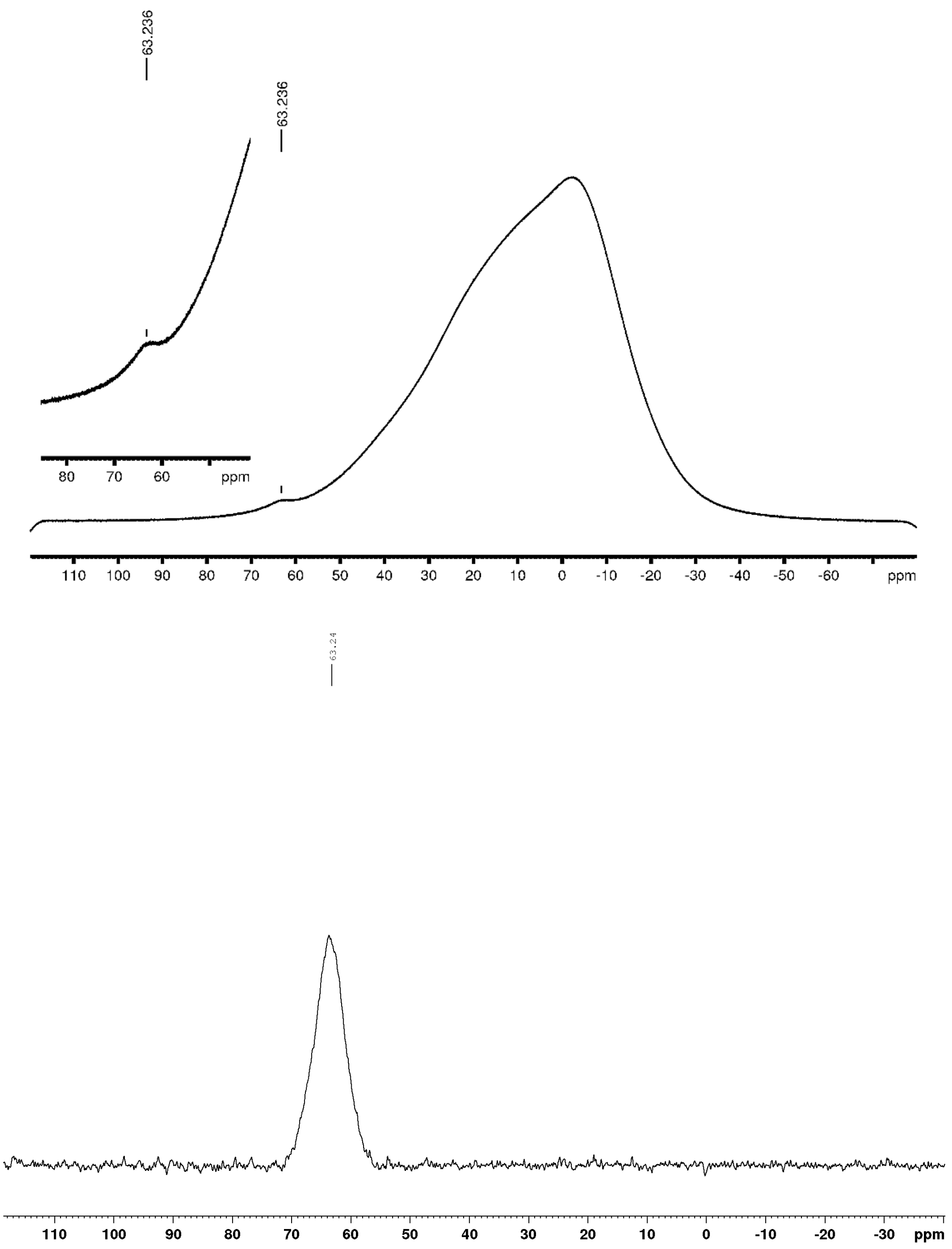

Figure S13: $^{11}B\{^{1}H\}$ NMR spectrum (160 MHz, 298 K) of **^{F}Xyl^{F}Bf** recorded in C_6D_6 (top) and baseline corrected spectrum (bottom).

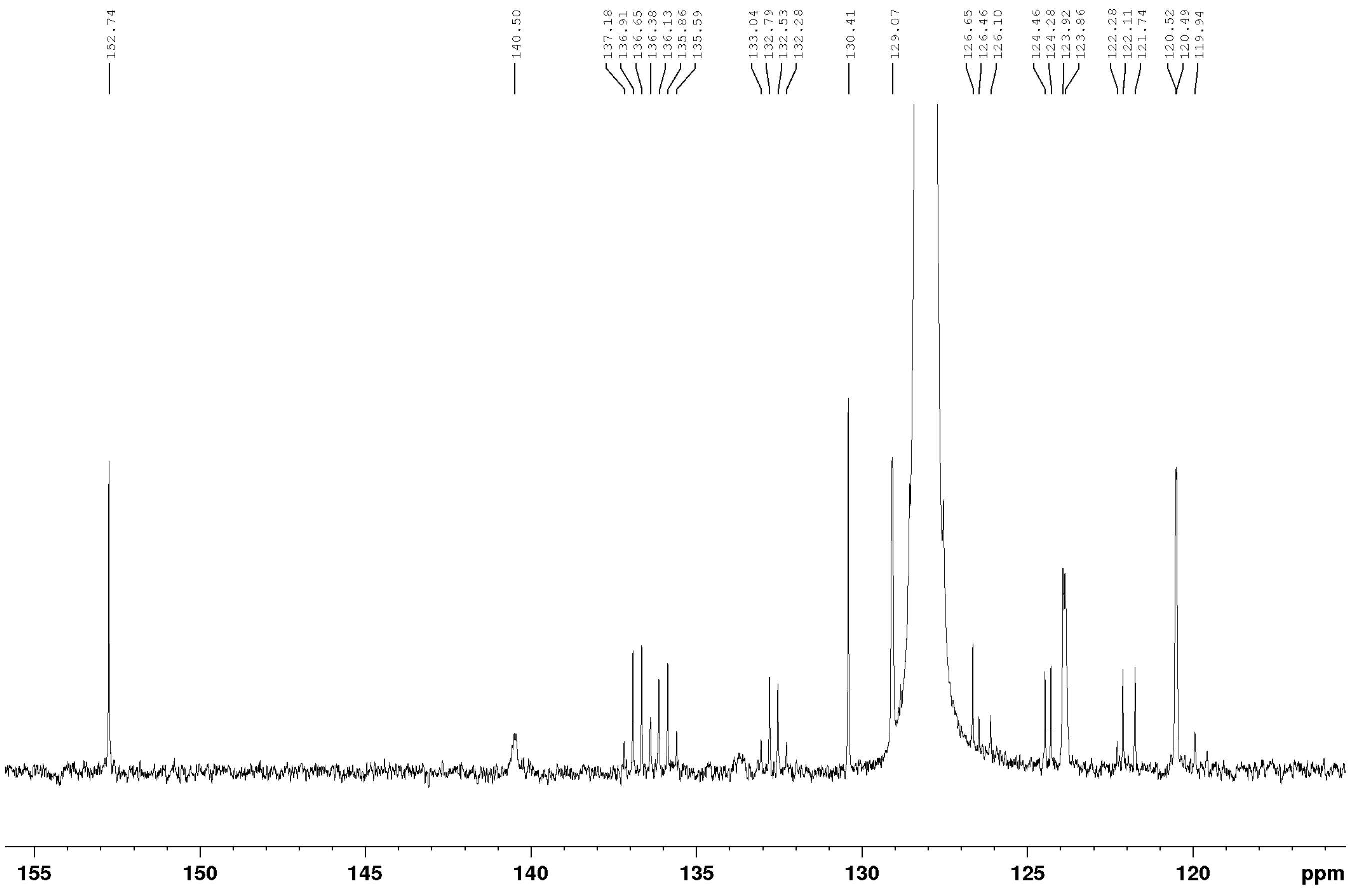

Figure S14: $^{13}C\{^{1}H\}$ NMR spectrum (126 MHz, 298 K) of **^{F}Xyl^{F}Bf** recorded in C_6D_6.

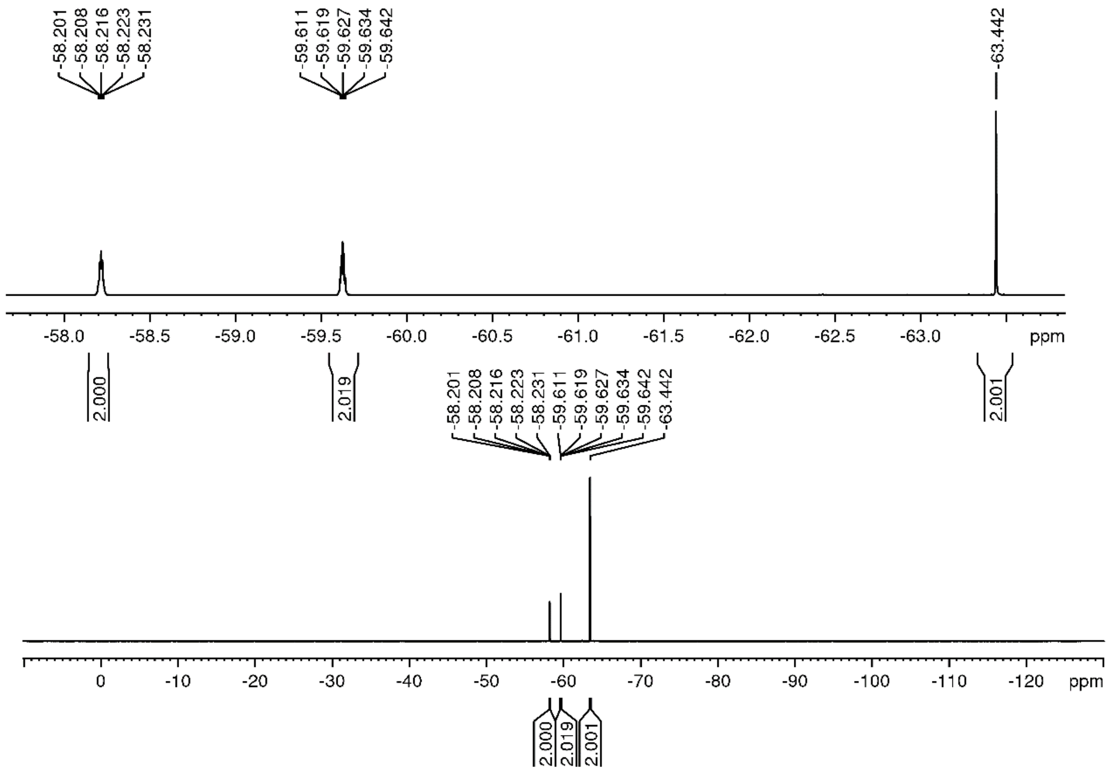

Figure S15: $^{19}F\{^{1}H\}$ NMR spectrum (471 MHz, 298 K) of **^{F}Xyl^{F}Bf** recorded in C_6D_6.

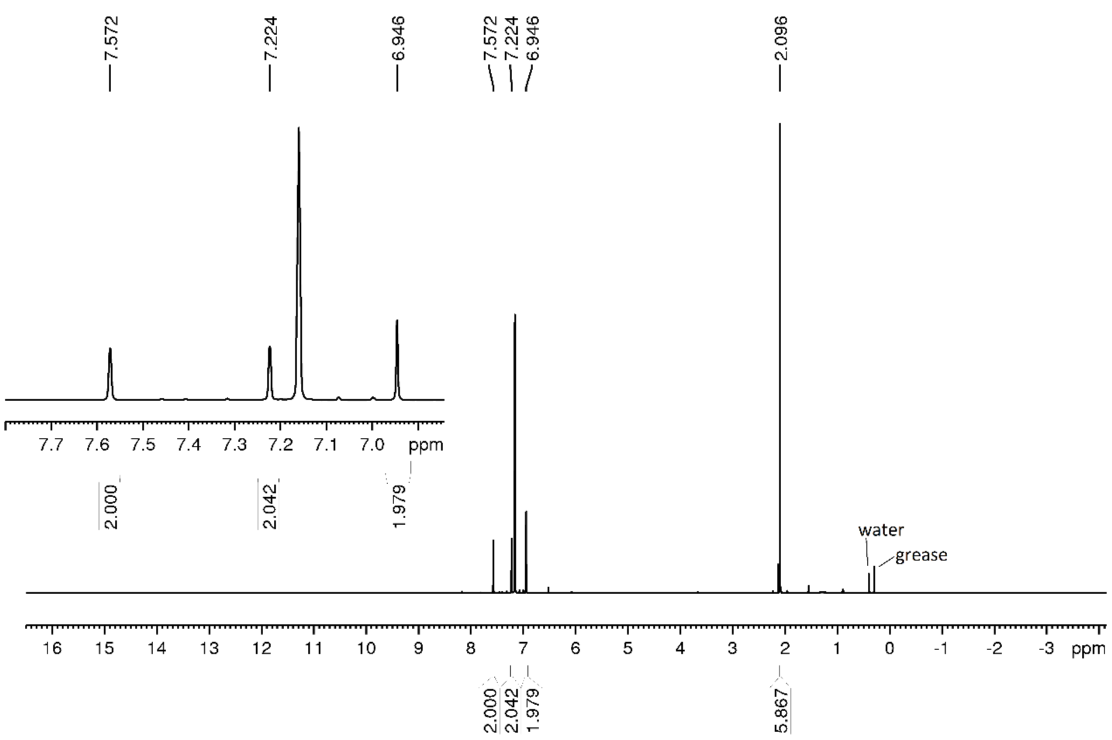

Figure S16: ^{1}H NMR spectrum (500 MHz, 298 K) of ***p*-NMe$_2$-^{F}Xyl^{F}Bf** recorded in C_6D_6.

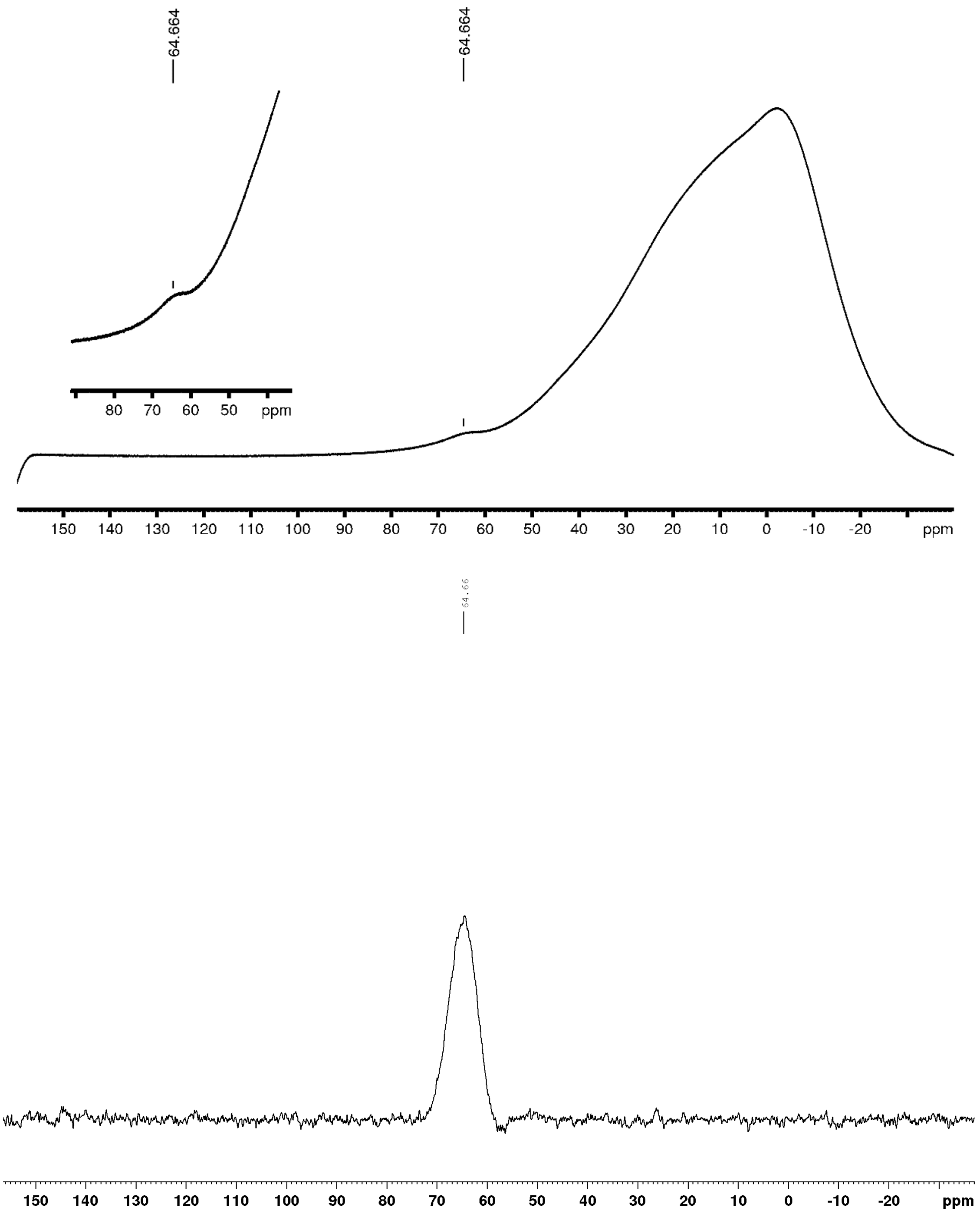

Figure S17: $^{11}B\{^{1}H\}$ NMR spectrum (160 MHz, 298 K) of ***p*-NMe$_2$-^{F}Xyl^{F}Bf** recorded in C_6D_6 (top) and baseline corrected spectrum (bottom).

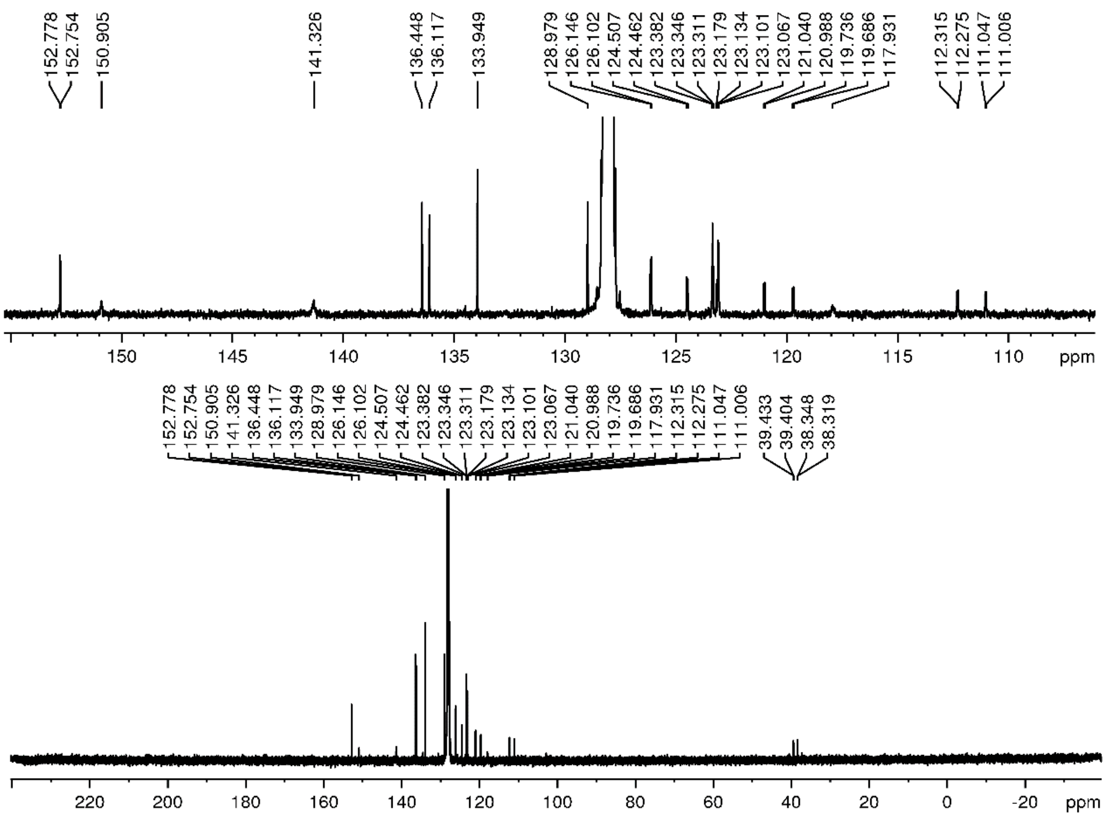

Figure S18: $^{13}C\{^{19}F\}$ NMR spectrum (126 MHz, 298 K) of ***p*-NMe$_2$-^{F}Xyl^{F}Bf** recorded in C_6D_6.

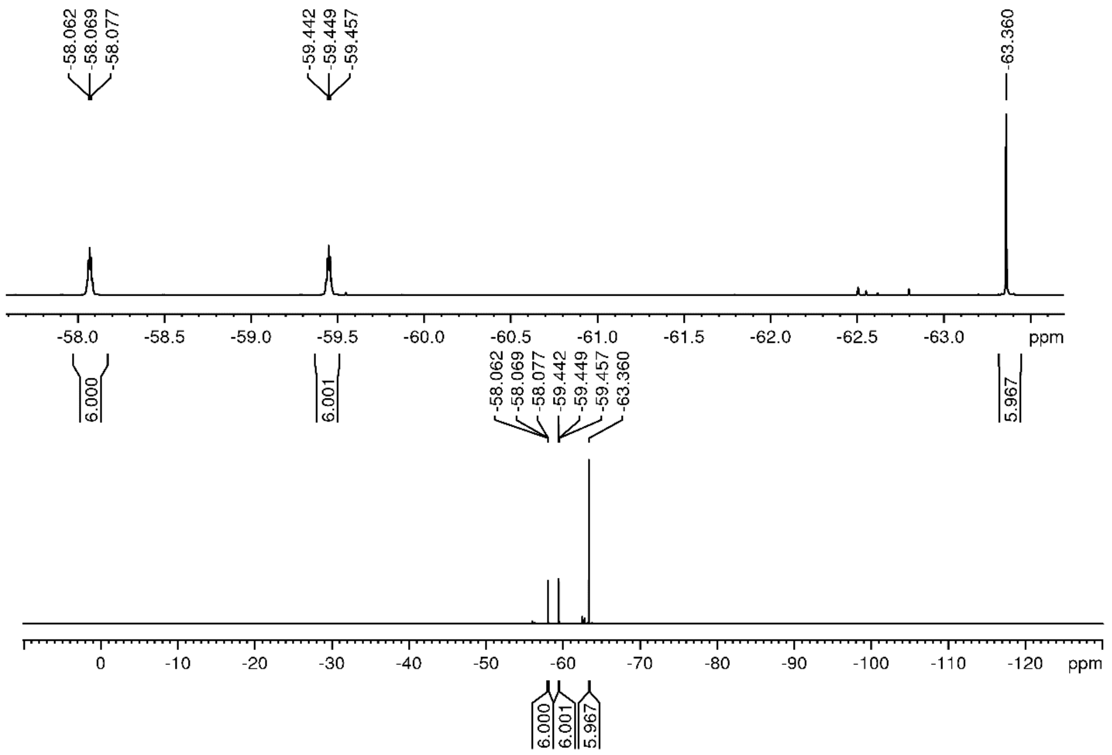

Figure S19: $^{19}F\{^{1}H\}$ NMR spectrum (471 MHz, 298 K) of ***p*-NMe$_2$-^{F}Xyl^{F}Bf** recorded in C_6D_6.

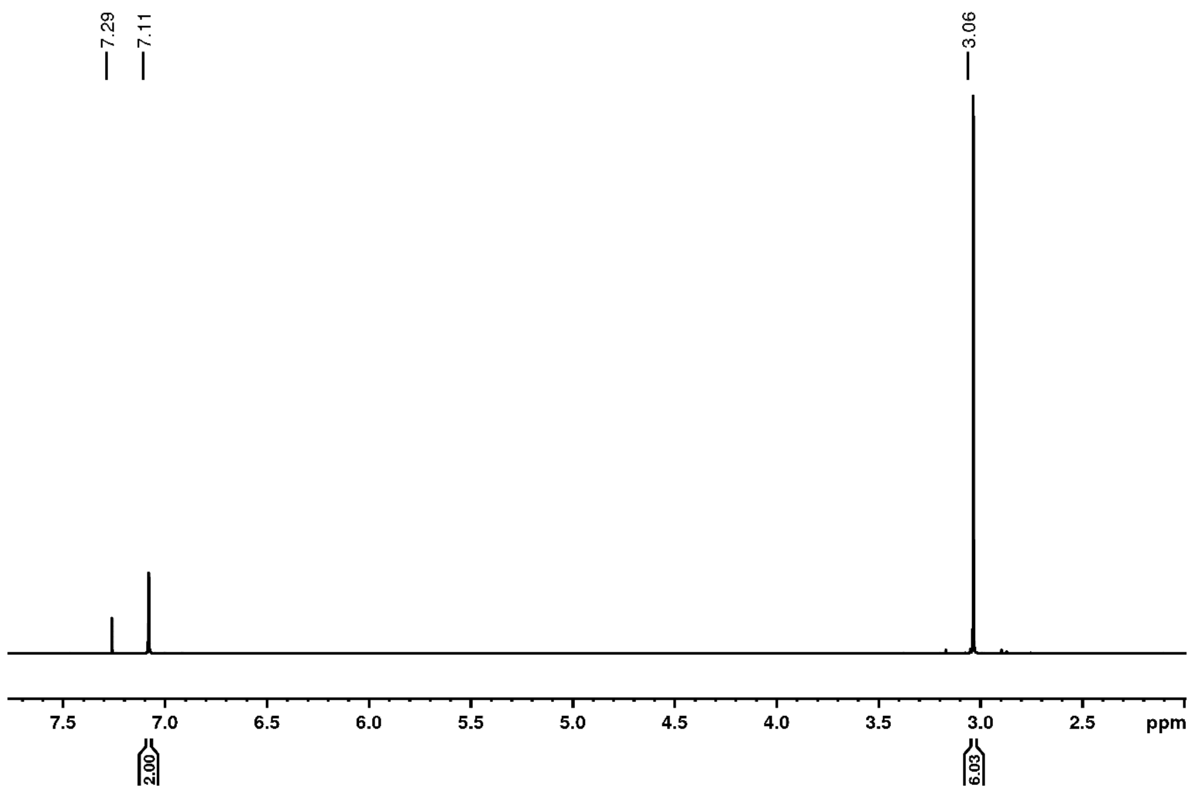

Figure S20: ^{1}H NMR spectrum (500 MHz, 298 K) of **4-(dimethylamino)-2,6-bis(trifluoromethyl)phenylbromide** recorded in $CDCl_3$.

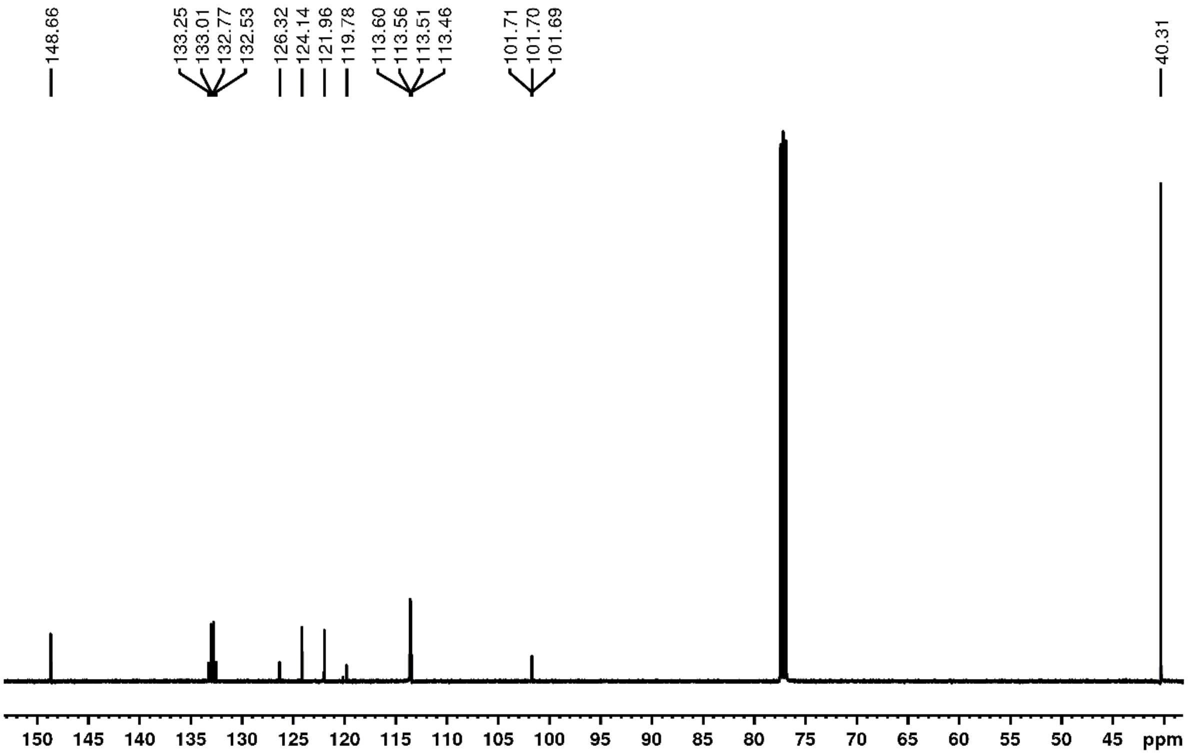

Figure S21: $^{13}C\{^{1}H\}$ NMR spectrum (126 MHz, 298 K) of **4-(dimethylamino)-2,6-bis(trifluoromethyl)phenylbromide** recorded in $CDCl_3$.

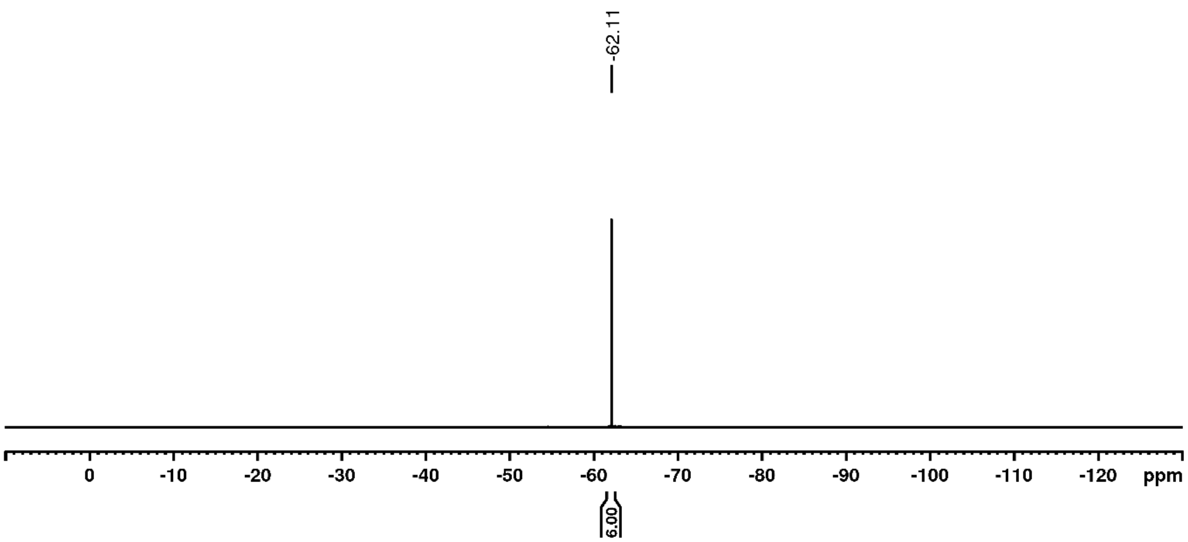

Figure S22: $^{19}F\{^{1}H\}$ NMR spectrum (471 MHz, 298 K) of **4-(dimethylamino)-2,6-bis(trifluoromethyl)phenylbromide** recorded in $CDCl_3$.

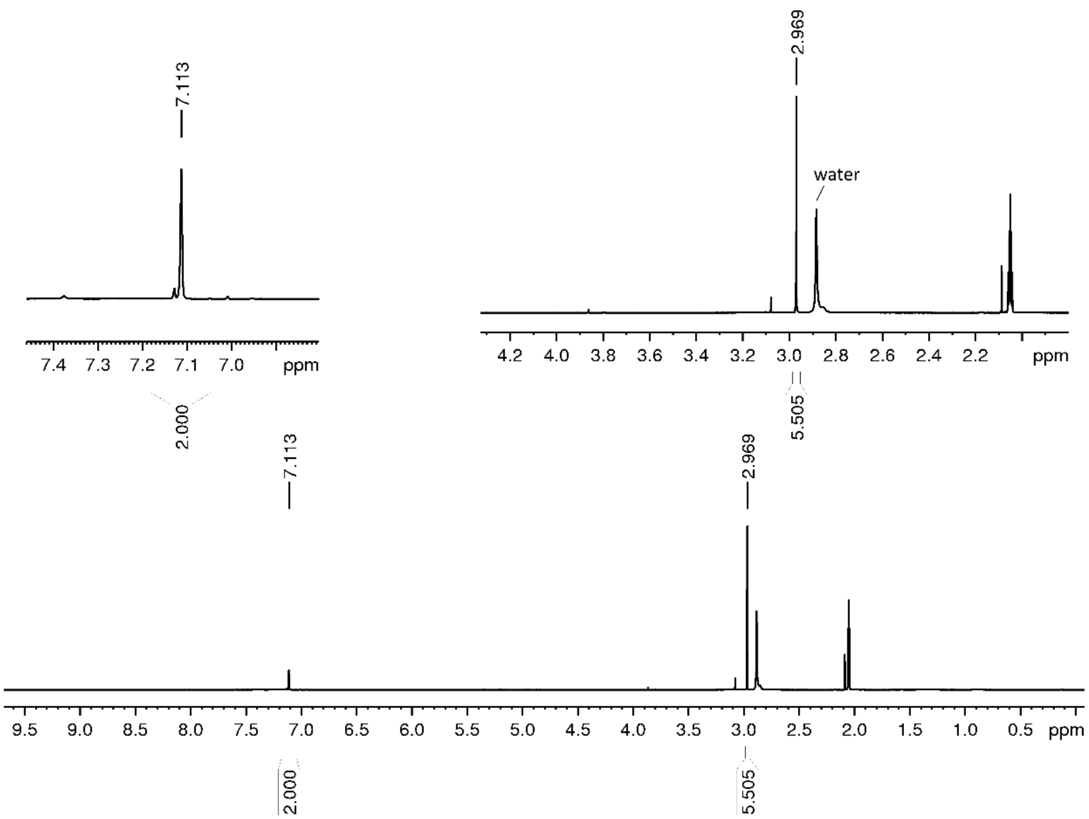

Figure S23: ^{1}H NMR spectrum (500 MHz, 298 K) of potassium **(4-(dimethylamino)-2,6-bis(trifluoromethyl)phenyl)trifluoroborate** recorded in acetone-d_6.

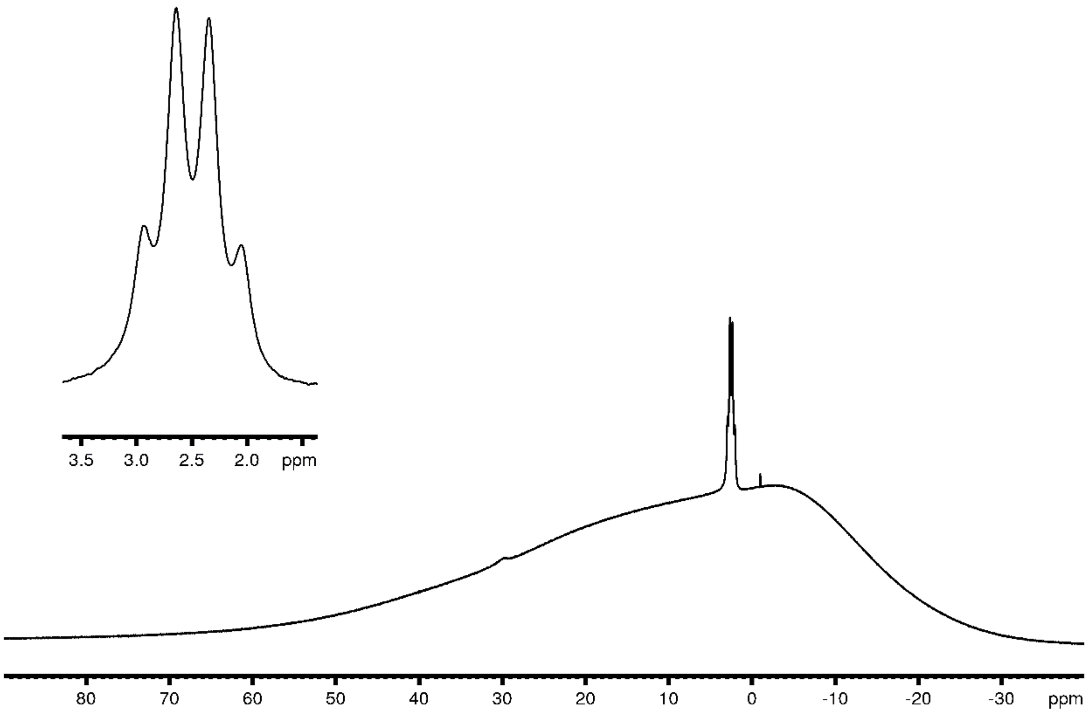

Figure S24: $^{11}B\{^1H\}$ NMR spectrum (160 MHz, 298 K) of **potassium (4-(dimethylamino)-2,6-bis(trifluoromethyl)phenyl)trifluoroborate** recorded in acetone-d_6.

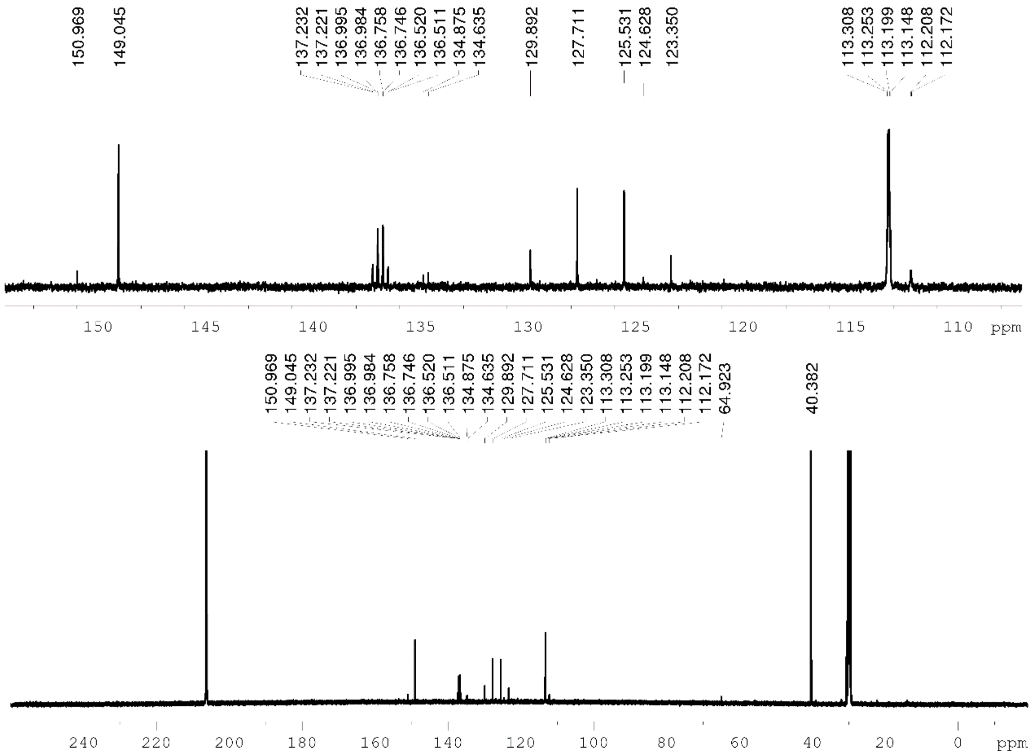

Figure S25: $^{13}C\{^1H\}$ NMR spectrum (126 MHz, 298 K) of **potassium (4-(dimethylamino)-2,6-bis(trifluoromethyl)phenyl)trifluoroborate** recorded in acetone-d_6.

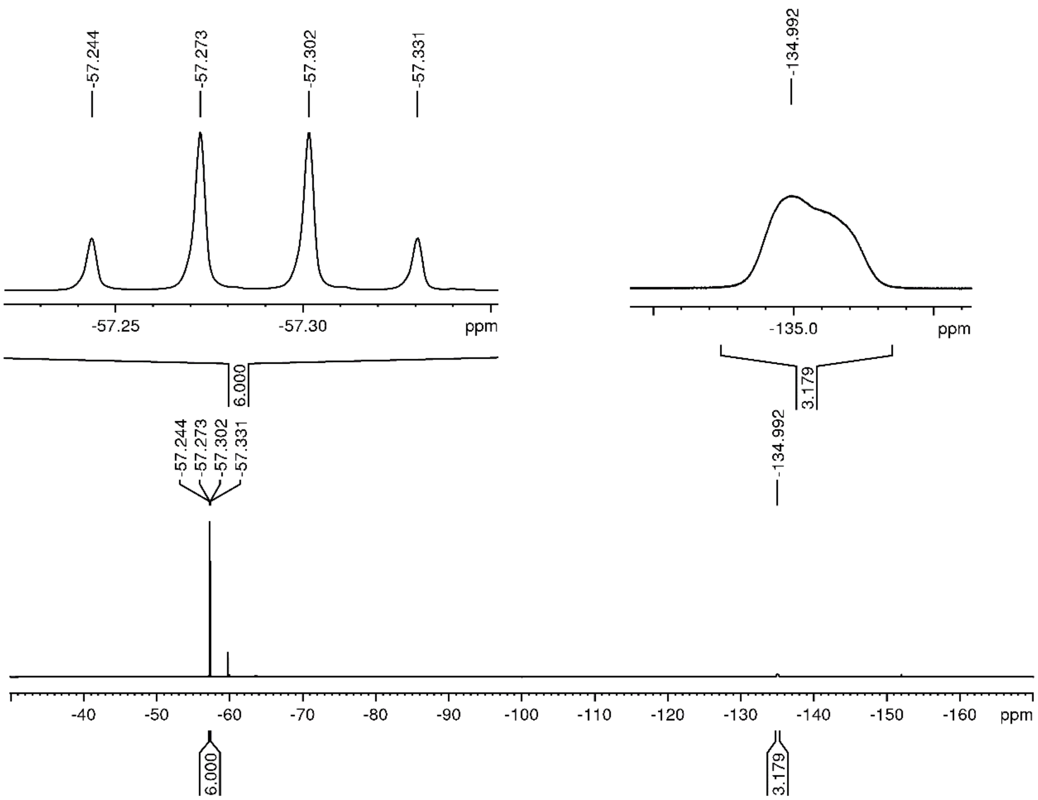

Figure S26: $^{19}F\{^{1}H\}$ NMR spectrum (471 MHz, 298 K) of **potassium (4-(dimethylamino)-2,6-bis(trifluoromethyl)phenyl)trifluoroborate** recorded in acetone-d_6.

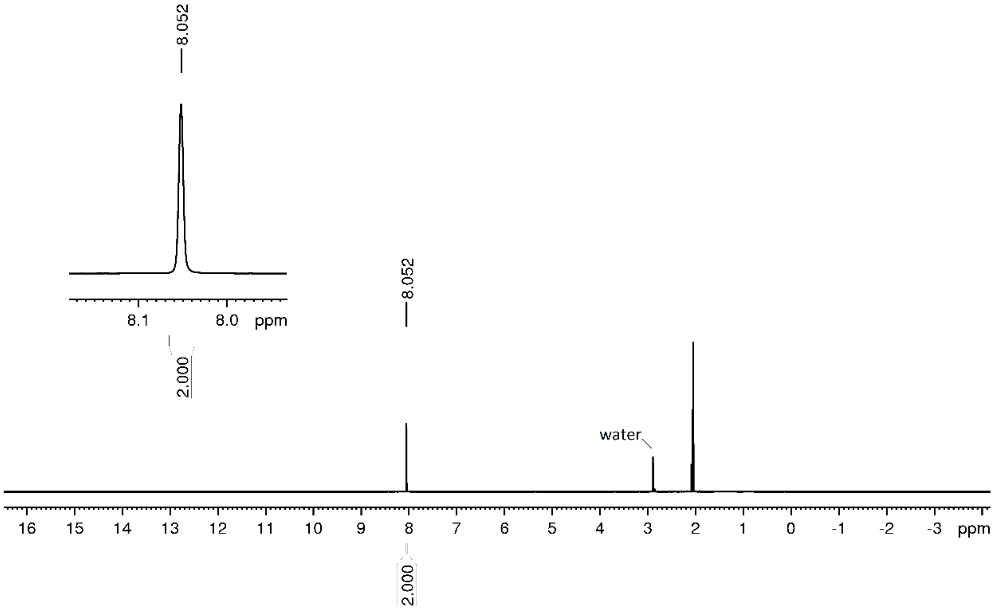

Figure S27: ^{1}H NMR spectrum (500 MHz, 298 K) of **potassium (2,4,6-tris(trifluoromethyl)phenyl)trifluoroborate** recorded in acetone-d_6.

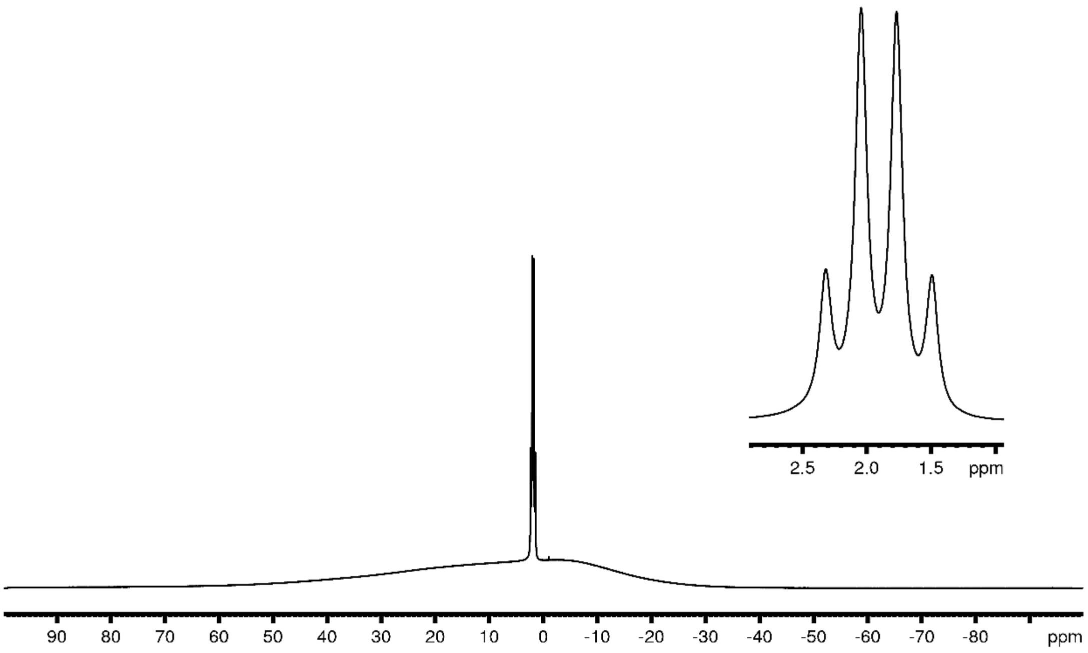

Figure S28: $^{11}B\{^{1}H\}$ NMR spectrum (160 MHz, 298 K) of **potassium (2,4,6-tris(trifluoromethyl)phenyl)trifluoroborate** recorded in acetone-d_6.

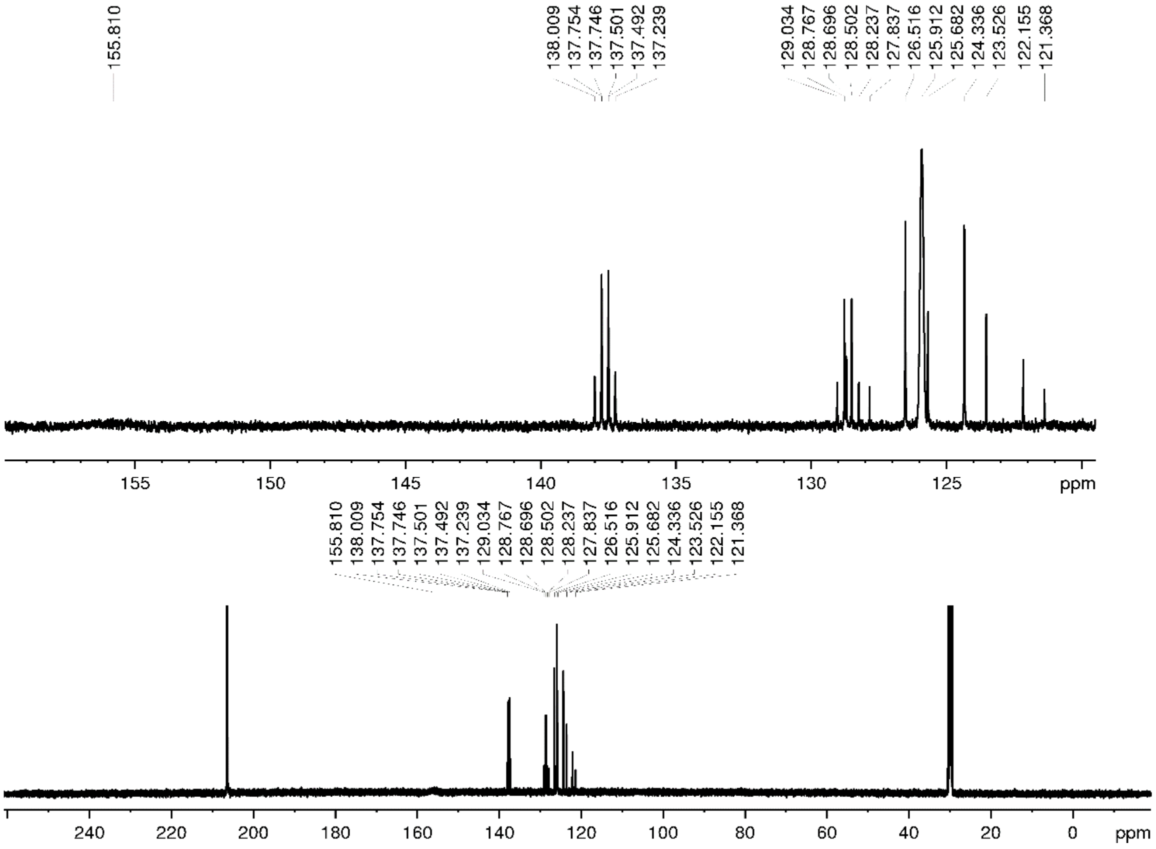

Figure S29: $^{13}C\{^{1}H\}$ NMR spectrum (126 MHz, 298 K) of **potassium (2,4,6-tris(trifluoromethyl)phenyl)trifluoroborate** recorded in acetone-d_6.

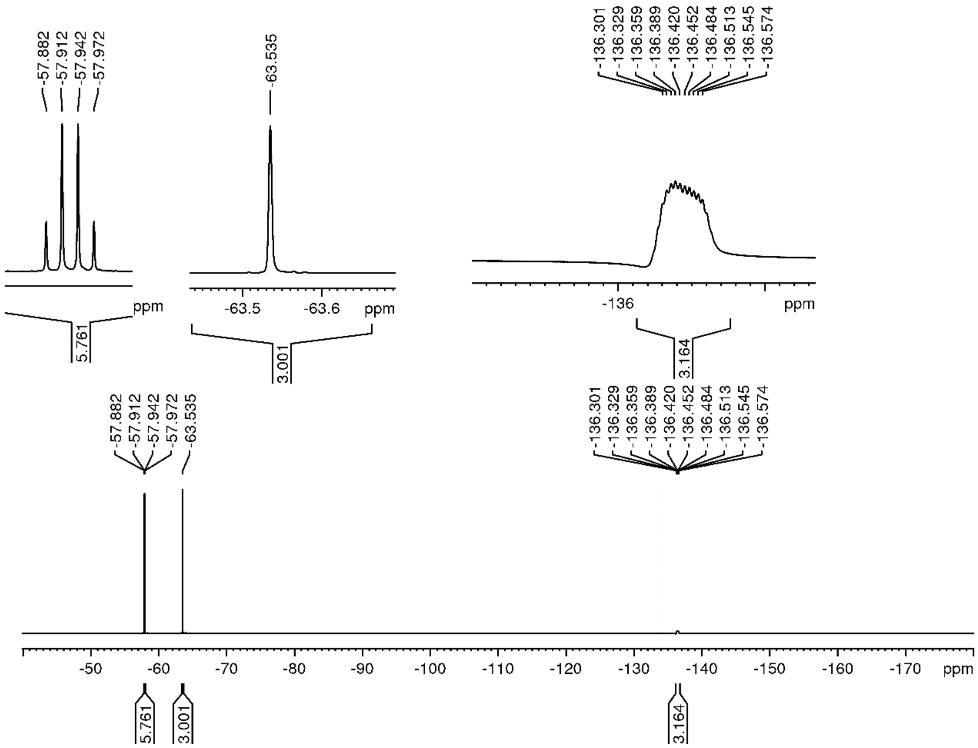

Figure S30: $^{19}F\{^1H\}$ NMR spectrum (471 MHz, 298 K) of **potassium (2,4,6-tris(trifluoromethyl)phenyl)trifluoroborate** recorded in acetone-d_6.

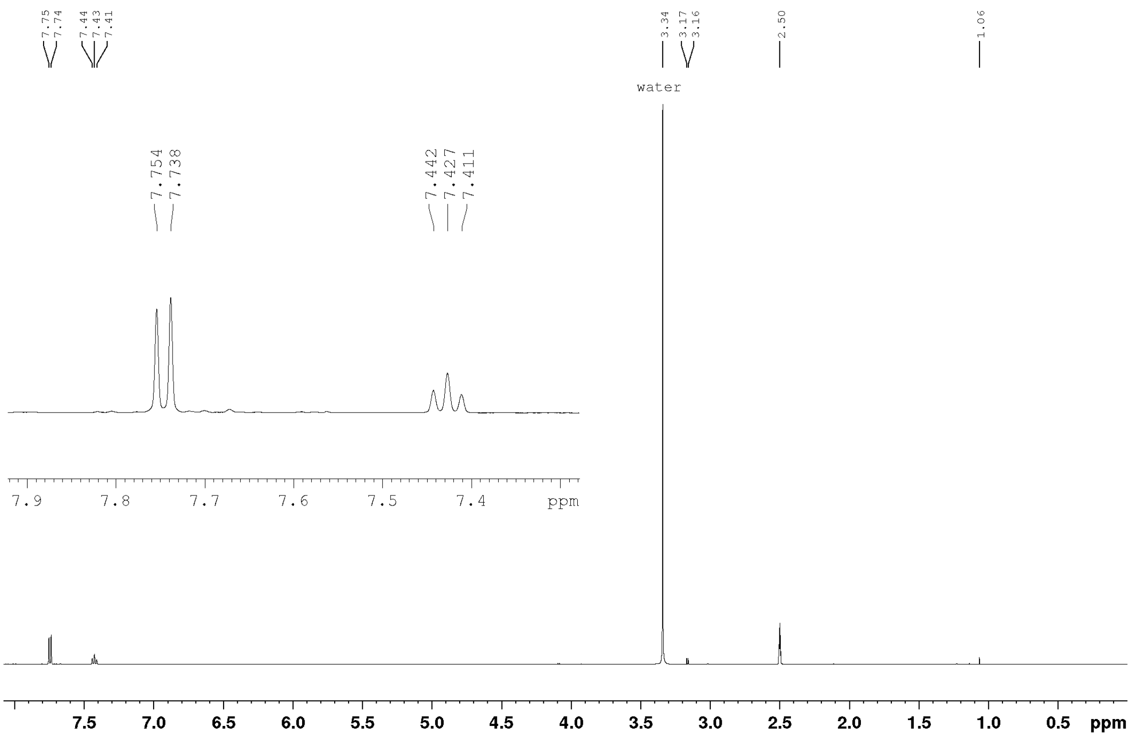

Figure S31: ^1H NMR spectrum (500 MHz, 298 K) of **potassium (2,6-bis(trifluoromethyl)phenyl)trifluoroborate** recorded in DMSO-d_6.

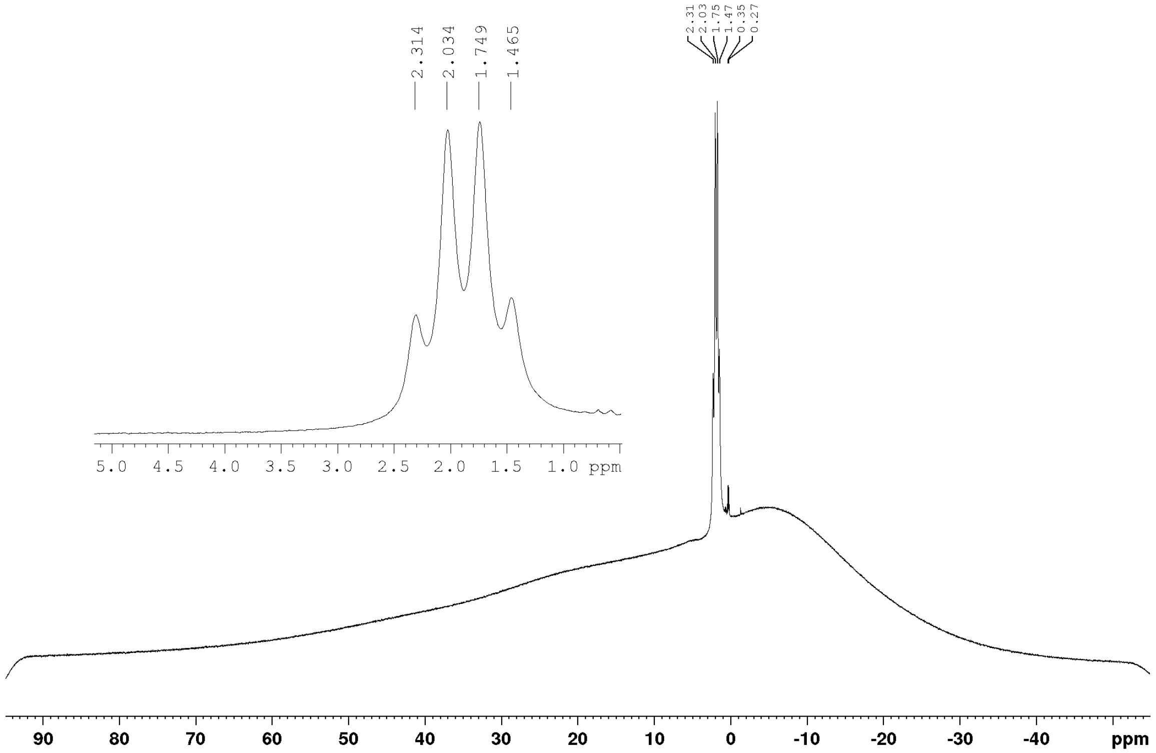

Figure S32: $^{11}B\{^{1}H\}$ NMR spectrum (160 MHz, 298 K) of **potassium (2,6-bis(trifluoromethyl)phenyl)trifluoroborate** recorded in DMSO-d_6.

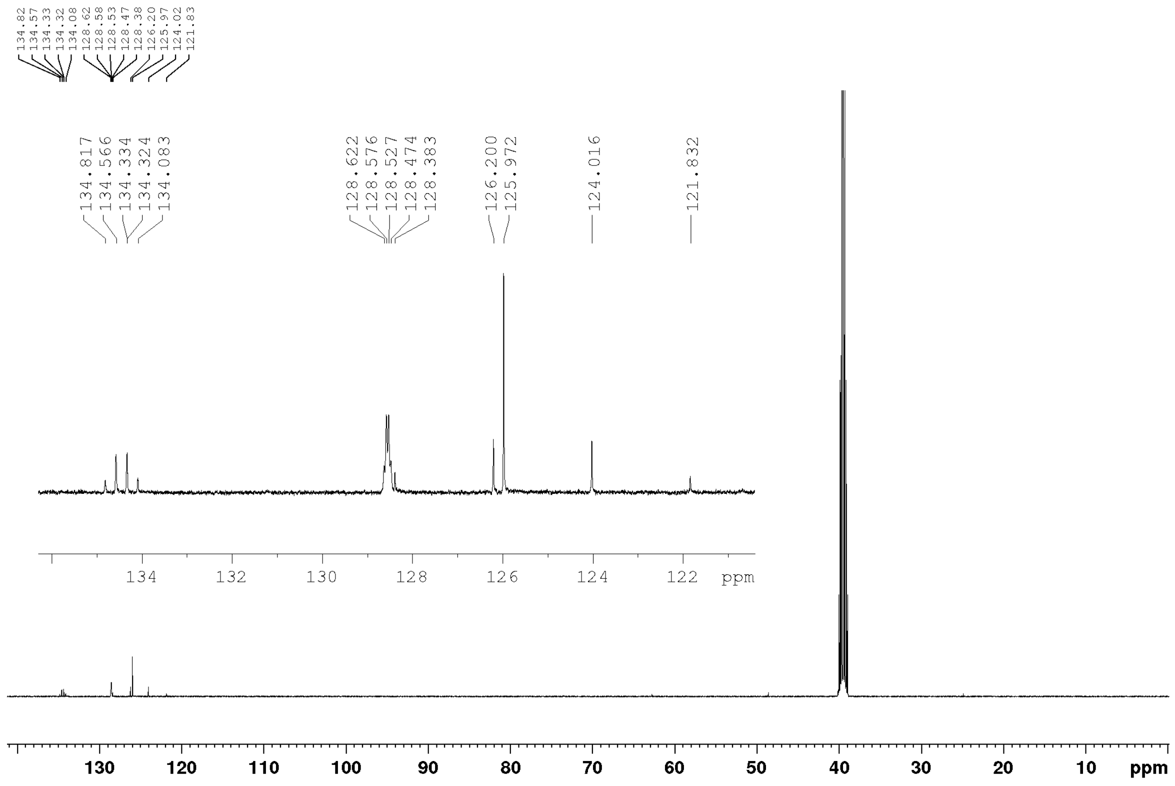

Figure S33: $^{13}C\{^{1}H\}$ NMR spectrum (126 MHz, 298 K) of **potassium (2,6-bis(trifluoromethyl)phenyl)trifluoroborate** recorded in DMSO-d_6.

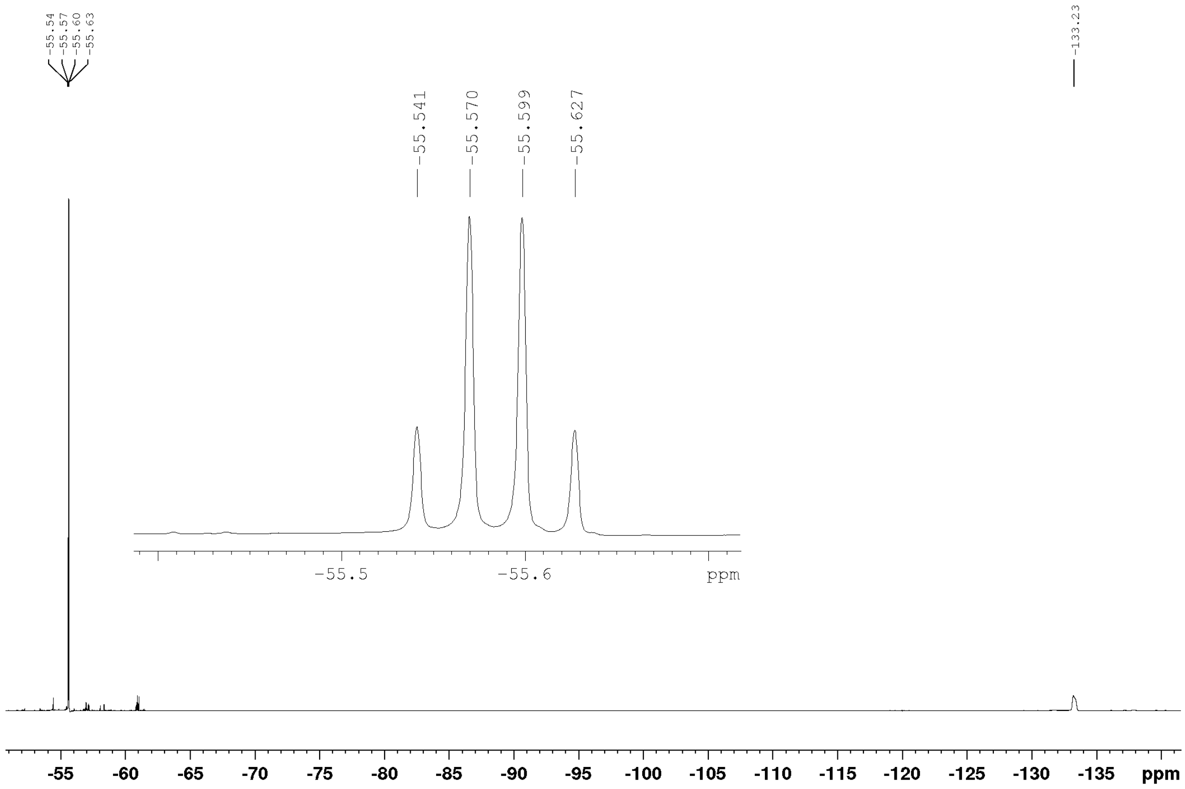

Figure S34: $^{19}F\{^1H\}$ NMR spectrum (471 MHz, 298 K) of **potassium (2,6-bis(trifluoromethyl)phenyl)trifluoroborate** recorded in DMSO-d_6.

Single-crystal X-ray diffraction

Table S1: Single-crystal X-ray diffraction data collection and structure refinement parameters for **^{F}Mes^{F}Bf**, **^{F}Xyl^{F}Bf**, ***p*-NMe$_2$-^{F}Xyl^{F}Bf**, **^{F}Mes^{F}Bf•MeCN**, **[^{F}Mes^{F}Bf]$^{\cdot-}$**, ***p*-NMe$_2$-^{F}Xyl^{F}Bf•HF**, compound **D**, **^{F}XylBF$_3$K**, compounds **1** and **2**.

Data	**^{F}Mes^{F}Bf**	**^{F}Xyl^{F}Bf**	***p*-NMe$_2$-^{F}Xyl^{F}Bf**
CCDC number	1940986	1940987	1940988
Empirical formula	$C_{25}H_6BF_{21}$, 0.5(C_6H_6)	$C_{24}H_7BF_{18}$	$C_{26}H_{12}BF_{18}N$
Formula weight / g·mol^{-1}	755.16	648.11	691.18
T / K	100(2)	100(2)	100(2)
λ / Å, radiation	MoKα 0.71073	MoKα 0.71073	MoKα 0.71073
Crystal size / mm³	0.14×0.23×0.26	0.15×0.19×0.36	0.07×0.24×0.33
Crystal color, habit	colorless block	colorless block	orange plate
μ / mm^{-1}	0.212	0.207	0.198
Crystal system	Monoclinic	Triclinic	Monoclinic
Space group	$C2/c$	$P\bar{1}$	$P2_1/c$
a / Å	21.958(12)	8.803(5)	14.222(6)
b / Å	10.228(6)	11.967(6)	21.197(11)
c / Å	25.424(14)	12.163(5)	8.482(4)
α / °	90	66.411(16)	90
β / °	110.60(3)	88.132(9)	94.355(13)
γ / °	90	85.47(2)	90
Volume / Å^3	5345(5)	1170.5(10)	2550(2)
Z	8	2	4
ρ_{calc} / g·cm^{-3}	1.877	1.839	1.801
F(000)	2968	636	1368
θ range / °	1.711 – 26.022	1.827 – 29.999	1.728 – 26.828
Reflections collected	22615	60818	30438
Unique reflections	5257	6785	5446
Parameters / restraints	451 / 0	388 / 0	417 / 0
GooF on F^2	1.051	1.050	1.034
R_1 [I>2σ(I)]	0.0350	0.0497	0.0386
wR^2 (all data)	0.0902	0.1123	0.0892
Max. / min. residual electron density / e·Å^{-3}	0.347 / –0.227	0.469 / –0.306	0.407 / –0.340

Table S1: Continued.

Data	**^{F}Mes^{F}Bf•MeCN**	**[^{F}Mes^{F}Bf]$^{•-}$**	***p*-NMe$_2$-^{F}Xyl^{F}Bf•HF**
CCDC number	1940989	1940990	1940991
Empirical formula	$C_{27}H_{9}BF_{21}N$	$C_{25}H_{6}BF_{21}$, $C_{10}H_{10}Co$, $C_{4}H_{8}O$	$C_{26}H_{13}BF_{19}N$, 2($C_{6}H_{6}$)
Formula weight / g·mol^{-1}	757.16	977.32	867.40
T / K	100(2)	100(2)	100(2)
λ / Å, radiation	MoKα 0.71073	MoKα 0.71073	MoKα 0.71073
Crystal size / mm³	0.20×0.35×0.41	0.31×0.36×0.37	0.64×0.09×0.08
Crystal color, habit	colorless block	black block	colorless needle
μ / mm^{-1}	0.216	0.595	0.161
Crystal system	Monoclinic	Triclinic	Monoclinic
Space group	$P2_1/n$	$P\bar{1}$	$P2_1/n$
a / Å	9.496(5)	11.832(2)	14.243(10)
b / Å	12.874(3)	18.769(8)	15.061(12)
c / Å	21.574(6)	20.035(4)	17.978(16)
α / °	90	108.553(15)	90
β / °	93.218(18)	103.243(16)	110.26(3)
γ / °	90	107.35(2)	90
Volume / Å^3	2633.4(16)	3759(2)	3618(5)
Z	4	4	4
ρ_{calc} / g·cm^{-3}	1.910	1.727	1.592
F(000)	1488	1945	1744
θ range / °	1.466 – 26.888	1.830 – 28.426	1.583 – 26.449
Reflections collected	23439	94726	33520
Unique reflections	5756	18824	7422
Parameters / restraints	453 / 0	1237 / 126	565 / 186
GooF on F^2	1.059	1.019	0.978
R_1 [I>2σ(I)]	0.0424	0.0547	0.0557
wR^2 (all data)	0.1089	0.1487	0.1349
Max. / min. residual electron density / e·Å^{-3}	0.454 / –0.354	1.786 / –0.594	0.311 / –0.367

Table S1: Continued.

Data	**D**	**$^{F}XylBF_3K$**	**1**	**2**
CCDC number	1940992	1949706	1949707	1949708
Empirical formula	$C_{26}H_{14}BF_{18}NO$, $0.5(C_6H_{14})$	$C_8H_3BF_9K$	$C_{14}H_{14}BBrF_6O_2$	$C_{16}H_4Br_2F_{12}$
Formula weight / $g \cdot mol^{-1}$	752.28	320.01	418.97	584.01
T / K	100(2)	100(2)	100(2)	100(2)
λ / Å, radiation	MoKα 0.71073	MoKα 0.71073	MoKα 0.71073	MoKα 0.71073
Crystal size / mm³	0.52×0.39×0.28	0.40×0.40×0.08	0.36×0.33×0.18	0.44×0.12×0.08
Crystal color, habit	colorless block	colorless plate	colorless block	colorless needle
μ / mm^{-1}	0.179	0.590	2.596	4.872
Crystal system	Monoclinic	Orthorhombic	Monoclinic	Monoclinic
Space group	$P2_1/n$	*Pbca*	$P2_1/c$	$C2/c$
a / Å	17.913(7)	8.813(14)	10.281(4)	22.316(8)
b / Å	9.135(3)	9.512(13)	21.199(10)	13.744(5)
c / Å	18.163(6)	26.11(4)	15.659(8)	5.760(3)
α / °	90	90	90	90
β / °	92.026(16)	90	107.681(14)	104.795(11)
γ / °	90	90	90	90
Volume / $Å^3$	2970.2(18)	2189(6)	3252(3)	1708.1(12)
Z	4	8	8	4
ρ_{calc} / $g \cdot cm^{-3}$	1.682	1.942	1.712	2.271
F(000)	1508	1248	1664	1112
θ range / °	2.244 – 27.575	1.560 – 26.717	1.921 – 26.022	1.757 – 30.507
Reflections collected	54695	16096	34308	15357
Unique reflections	6859	2299	6406	2618
Parameters / restraints	457 / 1	172 / 0	613 / 438	136 / 0
GooF on F^2	1.027	1.041	1.023	1.010
R_1 [I>2σ(I)]	0.0384	0.0310	0.0569	0.0318
wR^2 (all data)	0.0984	0.0716	0.1416	0.0817
Max. / min. residual electron density / $e \cdot Å^{-3}$	0.655 / –0.497	0.390 / -0.265	1.364 / –0.959	1.192 / –0.420

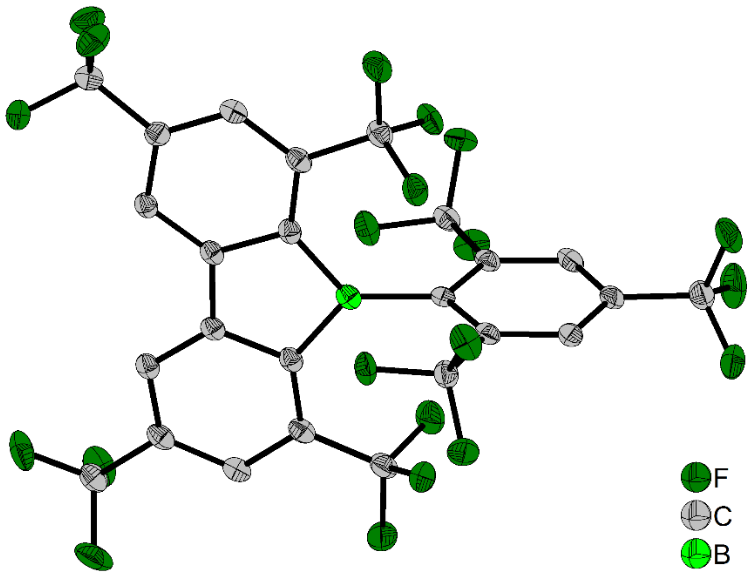

Figure S35: The solid-state molecular structure of ^{F}Mes^{F}**Bf** determined by single-crystal X-ray diffraction at 100 K. All ellipsoids are drawn at the 50% probability level. H atoms and the solvent molecule (benzene) are omitted for clarity.

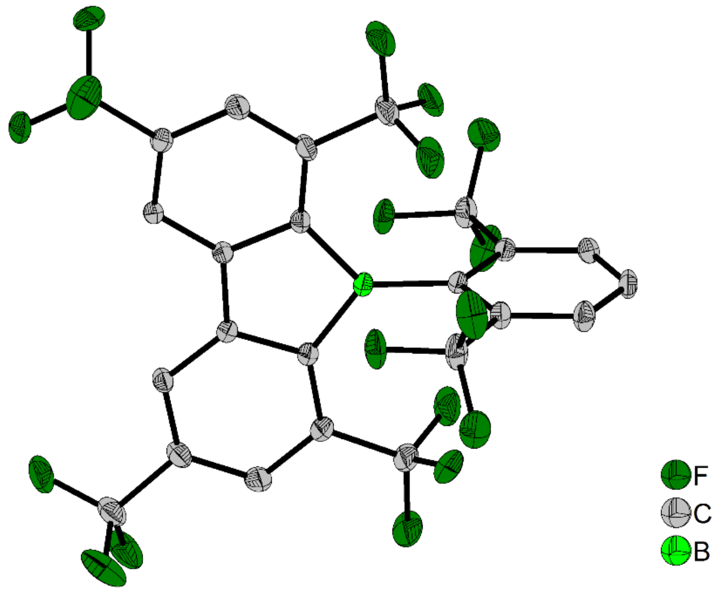

Figure S36: The solid-state molecular structure of ^{F}**Xyl**^{F}**Bf** determined by single-crystal X-ray diffraction at 100 K. All ellipsoids are drawn at the 50% probability level, and H atoms are omitted for clarity.

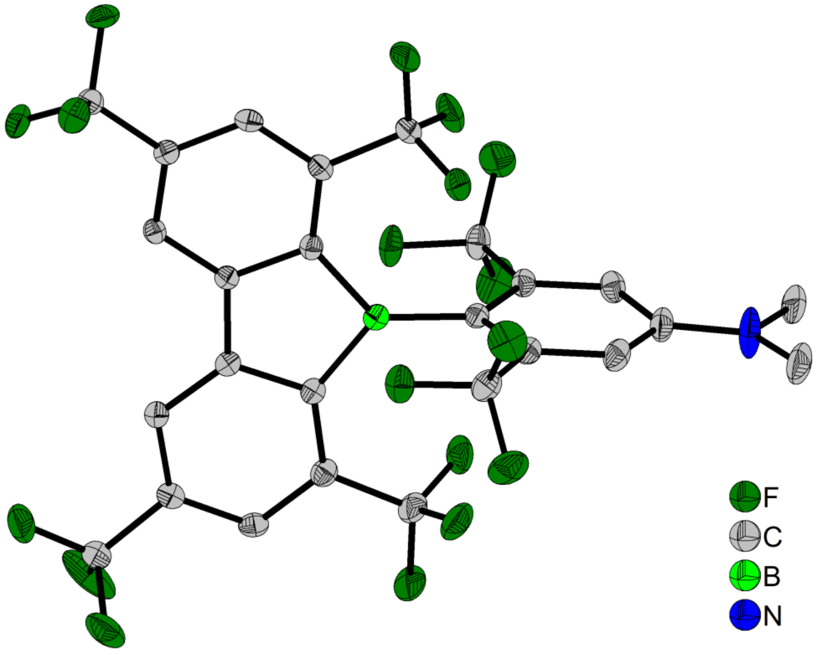

Figure S37: The solid-state molecular structure of ***p*-NMe$_2$-^{F}Xyl^{F}Bf** determined by single-crystal X-ray diffraction at 100 K. All ellipsoids are drawn at the 50% probability level, and H atoms are omitted for clarity.

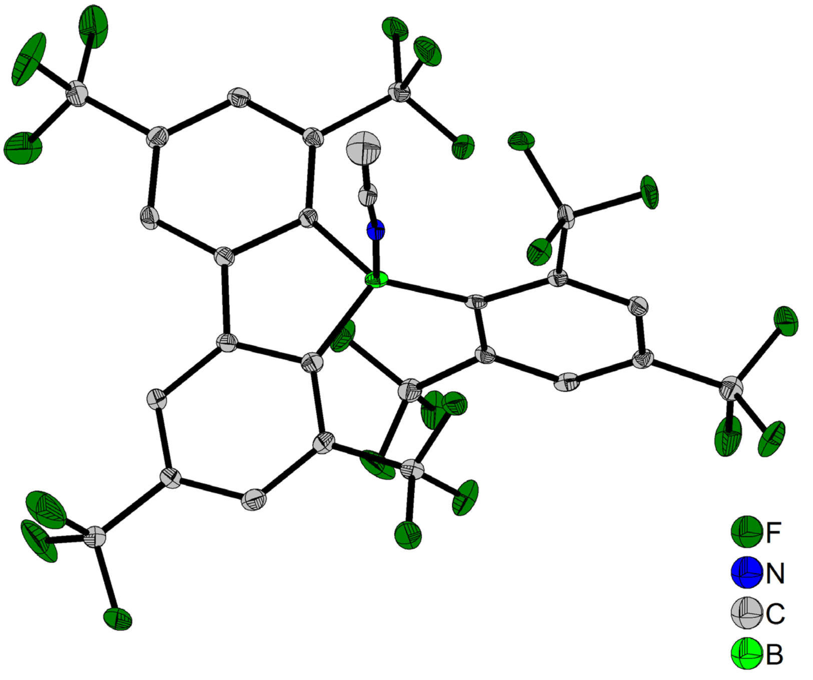

Figure S38: The solid-state molecular structure of **^{F}Mes^{F}Bf•MeCN** determined by single-crystal X-ray diffraction at 100 K. All ellipsoids are drawn at the 50% probability level, and H atoms are omitted for clarity.

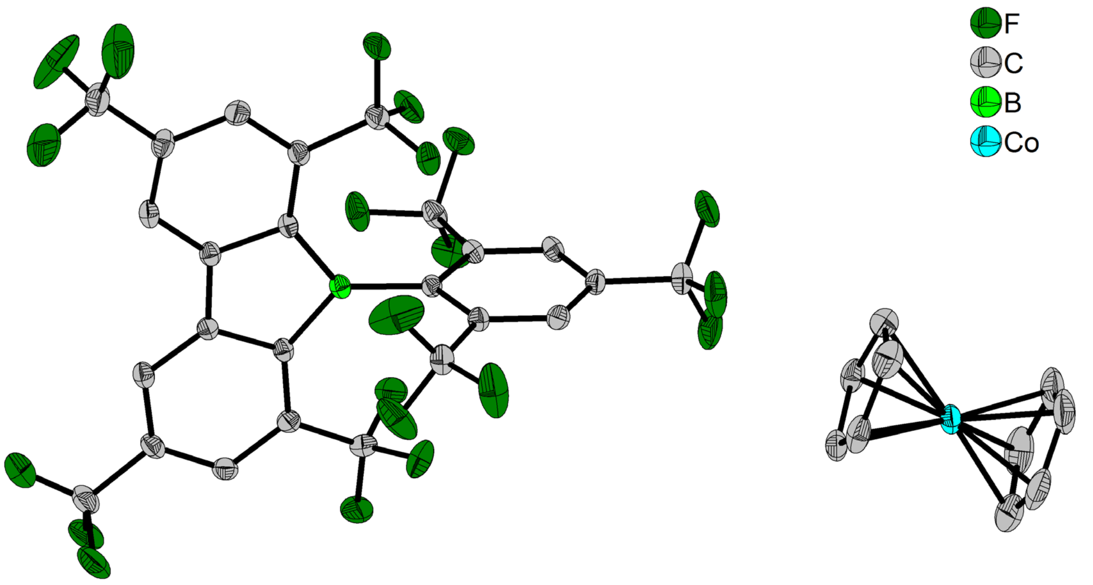

Figure S39: The solid-state molecular structure of **[^{F}Mes^{F}Bf]$^{\bullet-}$** determined by single-crystal X-ray diffraction at 100 K. All ellipsoids are drawn at the 50% probability level. H atoms and THF solvent molecules are omitted for clarity. Only half of the symmetrically non-equivalent molecules are shown. One of the CF_3 groups is rotationally disordered and only the part with the higher occupancy (64%) is shown here.

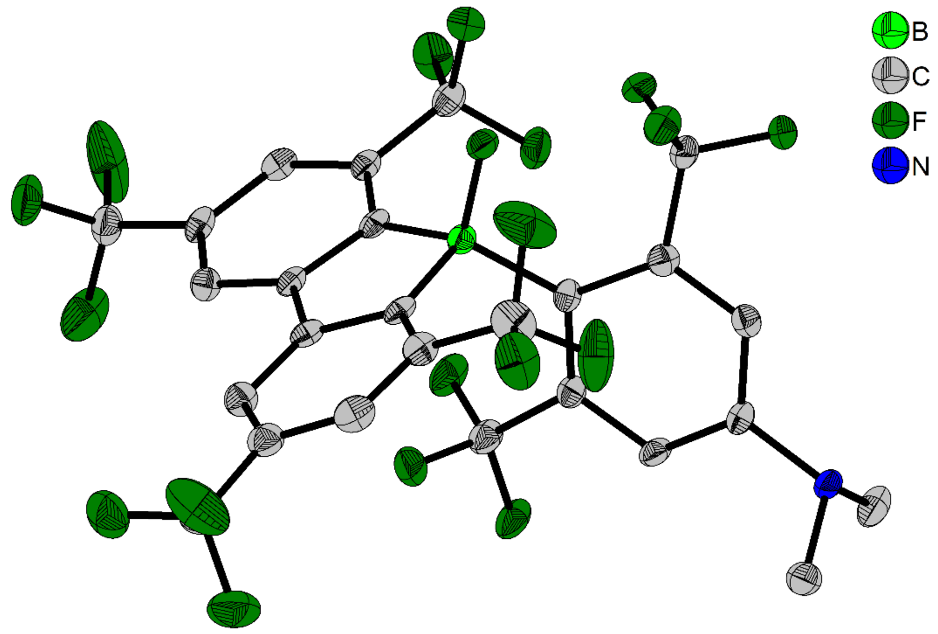

Figure S40: The solid-state molecular structure of ***p*-NMe$_2$-^{F}Xyl^{F}Bf•HF** determined by single-crystal X-ray diffraction at 100 K. All ellipsoids are drawn at the 50% probability level. H atoms and benzene solvent molecules are omitted for clarity.

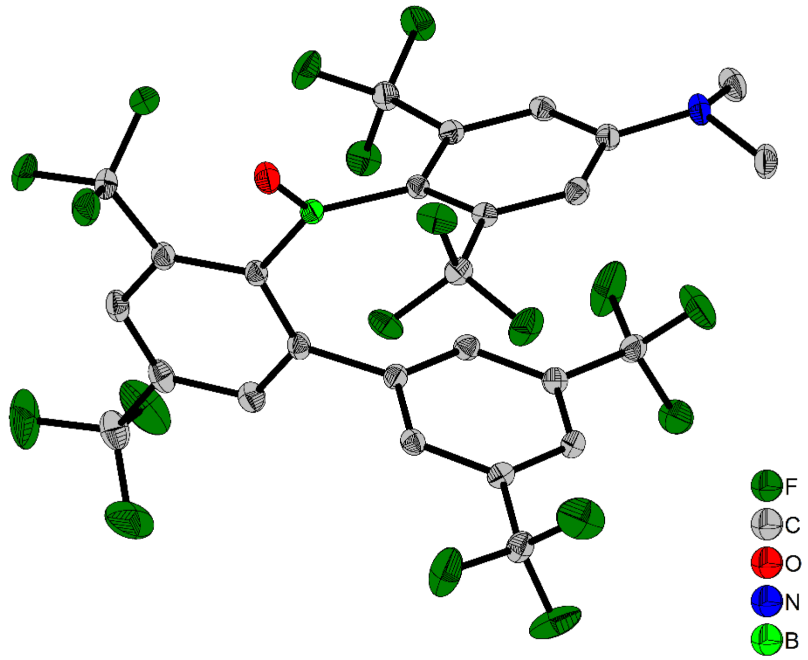

Figure S41: The solid-state molecular structure of compound **D** determined by single-crystal X-ray diffraction at 100 K. All ellipsoids are drawn at the 50% probability level. H atoms and the solvent molecule (hexane) are omitted for clarity.

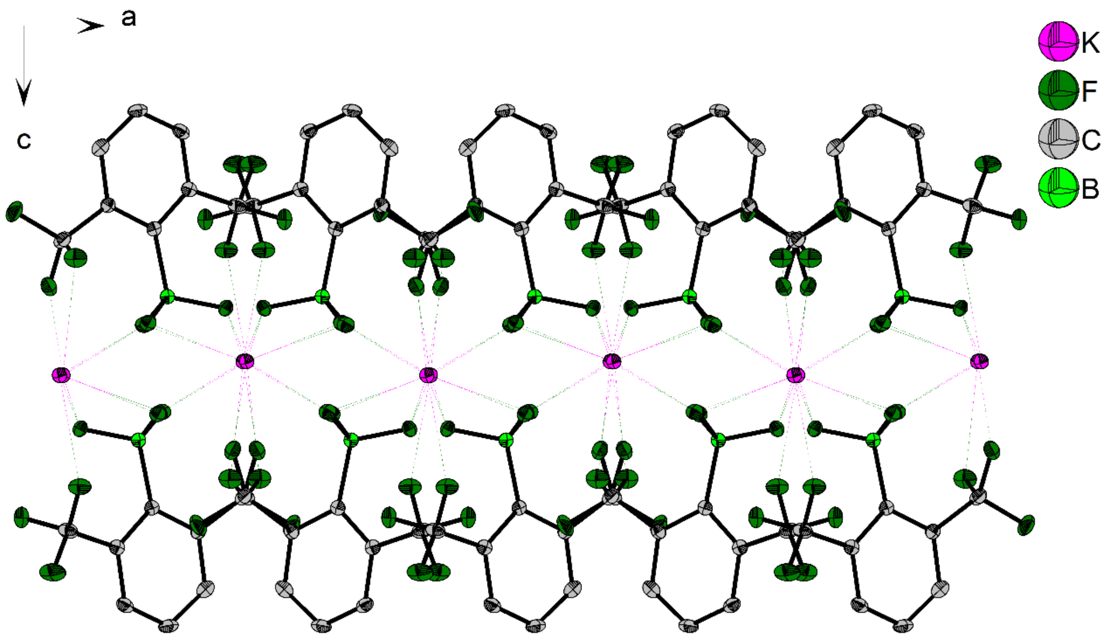

Figure S402: Projection of part of the crystal structure of **^{F}XylBF$_3$K** along the *b* axis shows the connectivity in a layer-like unit which further extends in *a* and *b* directions. Layers are arranged along the *c* axis. The structure was determined by single-crystal X-ray diffraction at 100 K. All ellipsoids are drawn at the 50% probability level. H atoms are omitted for clarity.

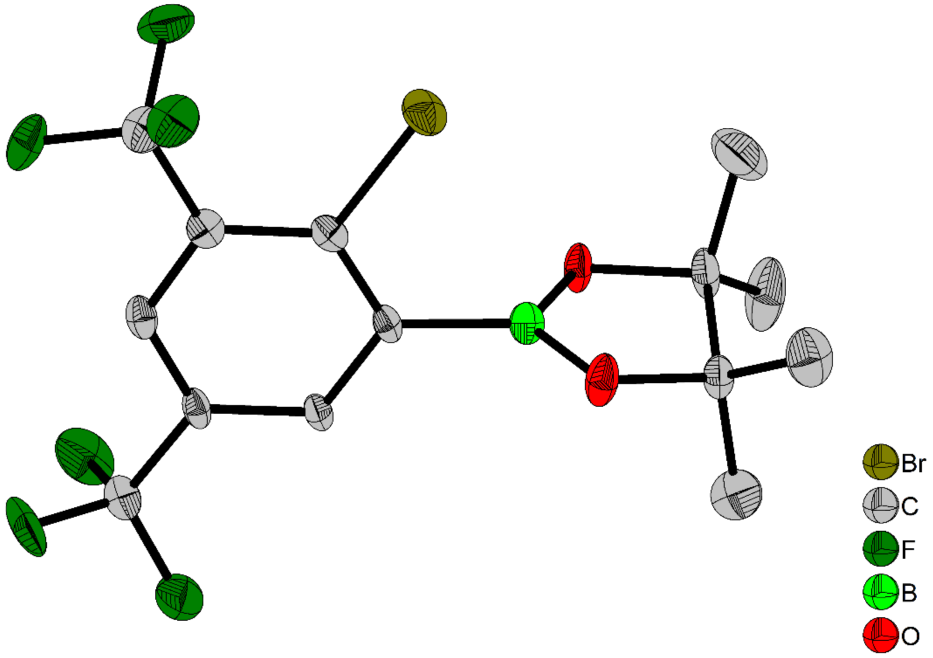

Figure S41: The solid-state molecular structure of compound **1** determined by single-crystal X-ray diffraction at 100 K. All ellipsoids are drawn at the 50% probability level. H atoms are omitted for clarity. Only one of two symmetrically non-equivalent molecules is shown. Part of this molecule is disordered and only the part with the higher occupancy (87%) is shown.

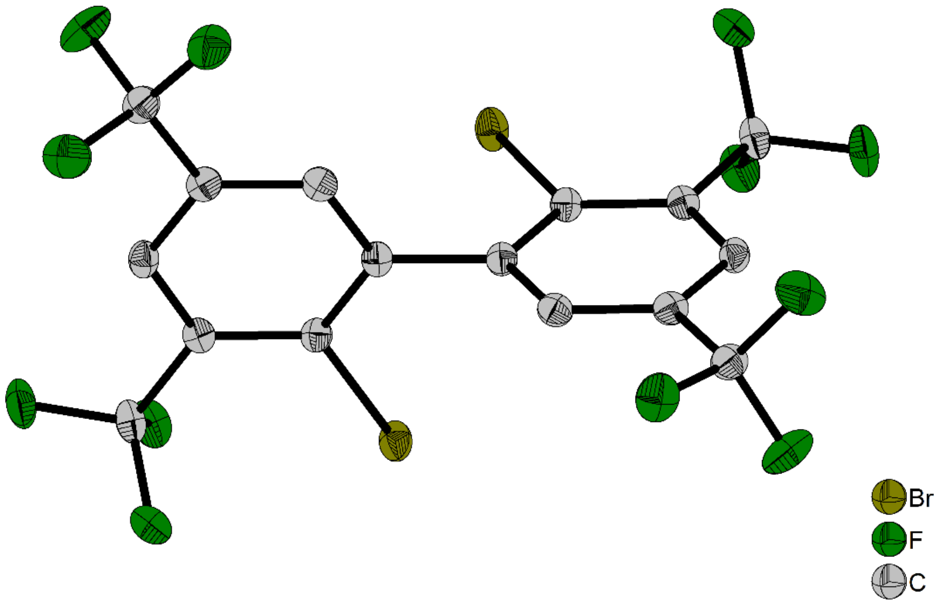

Figure S42: The solid-state molecular structure of compound **2** determined by single-crystal X-ray diffraction at 100 K. All ellipsoids are drawn at the 50% probability level. H atoms are omitted for clarity.

Table S2: Selected bond lengths (Å) and angles (°) of **^{F}Mes^{F}Bf**, **^{F}Xyl^{F}Bf**, ***p*-NMe$_2$-^{F}Xyl^{F}Bf**, **^{F}Mes^{F}Bf•MeCN**, and **[^{F}Mes^{F}Bf]$^{\bullet-}$**.

	^{F}Mes^{F}Bf	^{F}Xyl^{F}Bf	*p*-NMe$_2$-^{F}Xyl^{F}Bf	^{F}Mes^{F}Bf•MeCN	[^{F}Mes^{F}Bf]$^{\bullet-a}$ Molecule 1	[^{F}Mes^{F}Bf]$^{\bullet-a}$ Molecule 2
B–C1	1.579(3)	1.570(2)	1.570(3)	1.652(3)	1.587(4)	1.594(4)
B–C2	1.581(3)	1.591(3)	1.595(3)	1.645(3)	1.547(4)	1.556(4)
B–C3	1.591(3)	1.591(3)	1.591(3)	1.645(3)	1.548(4)	1.551(4)
B–N				1.591(3)		
C3–C19/C18/C20	1.408(3)	1.410(2)	1.409(2)	1.401(3)	1.438(3)	1.432(3)
C2–C18/C17/19	1.410(2)	1.409(2)	1.408(2)	1.410(3)	1.429(3)	1.432(3)
C18/C17/C19–C19/C18/C20	1.481(3)	1.474(2)	1.482(2)	1.475(3)	1.457(3)	1.457(3)
Exo-aryl:						
C1–C4	1.405(3)	1.400(2)	1.400(2)	1.419(3)	1.404(3)	1.405(3)
C1–C8	1.402(3)	1.401(2)	1.398(3)	1.428(3)	1.414(3)	1.417(3)
C4–C5	1.385(3)	1.390(2)	1.381(3)	1.399(3)	1.392(3)	1.389(4)
C8–C7	1.387(3)	1.387(2)	1.385(3)	1.383(3)	1.382(3)	1.382(3)
C5–C6	1.384(3)	1.380(3)	1.400(3)	1.372(3)	1.380(4)	1.380(4)
C7–C6	1.384(3)	1.380(3)	1.400(3)	1.373(3)	1.384(4)	1.384(4)
C6–N			1.373(3)			
C11–N			1.441(3)			
C12–N			1.431(3)			
∠ BC$_3$-Aryl$_{exo}$	89.32(7)	89.84(8)	89.27(6)		88.38(10)	89.87(10)
∠ BC$_{12}$- Aryl$_{exo}$	89.43(6)	89.54(6)	88.69(5)	82.12(6)	88.02(7)	88.71(7)
Torsion of Ar$_{exo}$ out of plane ∠ C1–B–C2–C18/C17/C19	171.28(16)	176.45(16)	178.83(17)	137.6(2)	173.2(2)	179.6(2)
5-ring:						
∠ C2BC3	103.95(15)	103.07(13)	102.94(14)	99.74(18)	104.3(2)	104.1(2)
∠ BC2C	107.01(16)	107.41(15)	107.46(15)	107.83(19)	107.8(2)	107.6(2)
∠ C2CC	110.98(17)	111.01(15)	111.01(15)	111.82(19)	110.3(2)	110.3(2)
∠ C3CC	111.41(15)	111.31(14)	111.05(15)	111.60(19)	110.0(2)	110.2(2)
∠ BC3C	106.57(16)	107.20(14)	107.52(15)	108.46(19)	107.5(2)	107.7(2)
Sum ∠ CBC	359.74(16)	359.90(15)	359.99(16)	338.43(18)	359.8(2)	360.0(2)
Sum ∠ CNC			359.84(19)			
Shortest B–F contact(s)	2.392(3)<br>2.440(3)	2.379(2)<br>2.390(3)	2.366(2)<br>2.434(2)	2.853(3)	2.637(4)	2.615(4)<br>2.886(3)

[a]Two independent molecules are present in the unit cell. Hence, values are given for both molecules.

Table S3: Aryl···aryl (π···π) distances (Å) in crystals of **^{F}Mes^{F}Bf**, **^{F}Xyl^{F}Bf**, and ***p*-NMe$_2$-^{F}Xyl^{F}Bf** at 100 K: centroid-centroid distance, interplanar separation, and offset shift.

**Aryl···Aryl**	**Centroid-centroid distance**	**Interplanar separation**	**Offset shift[a]**
**Compound ^{F}Mes^{F}B**f			
**dibenzoborole···dibenzoborole**	3.8028(18)	3.469(2)	1.557(3)
**Compound ^{F}Xyl^{F}B**f			
**dibenzoborole···dibenzoborole**	4.011(2)	3.565(2)	1.838(2)
**Compound *p*-NMe$_2$-^{F}Xyl^{F}B**f			
**dibenzoborole···dibenzoborole**	4.0116(15)	3.5655(18)	1.839(3)

[a] The offset shift, also called inter-centroid shift, is the distance within a plane of an aryl ring between the centroid of the respective aryl ring and the intersection point with the normal to the plane through the centroid of the other aryl ring.

Electrochemistry

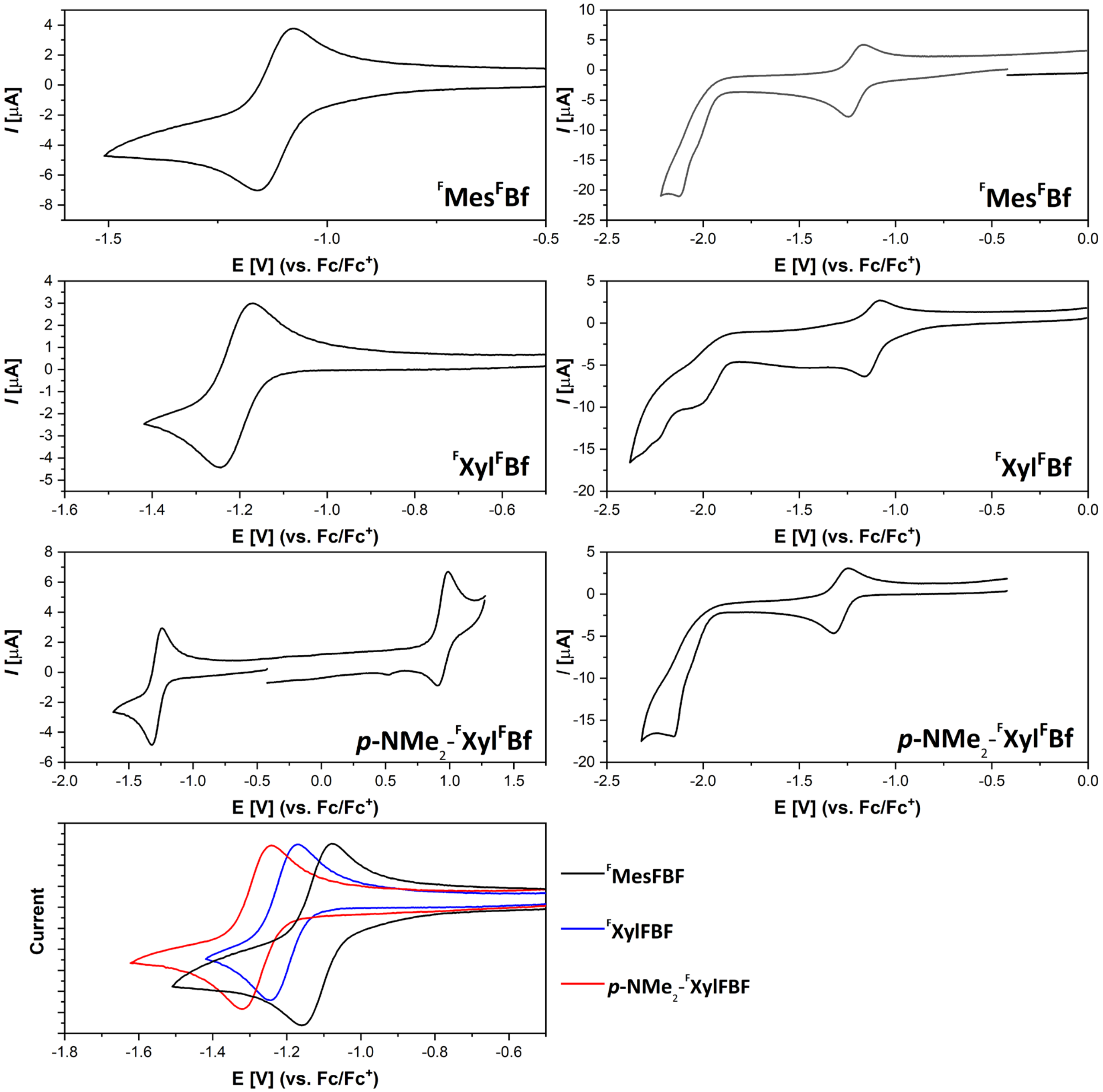

Figure S43: Cyclovoltammogramms of the reversible and irreversible redox events of **^{F}Mes^{F}Bf** (top), **^{F}Xyl^{F}Bf** (2nd row) and ***p*-NMe$_2$-^{F}Xyl^{F}Bf** (3rd row). For comparison, the reduction waves are plotted together (bottom; **^{F}Mes^{F}Bf** (black), **^{F}Xyl^{F}Bf** (blue), ***p*-NMe$_2$-^{F}Xyl^{F}Bf** (red)). The cyclovoltammogramms were measured in dichloromethane with [*n*Bu$_4$N][PF$_6$] as the electrolyte with a scan rate of 250 mVs^{-1}. All measurements are referenced to the Fc/Fc$^+$ ion couple.

Table S4: Reversible and irreversible reduction and oxidation potentials of **^{F}Mes^{F}Bf**, **^{F}Xyl^{F}Bf**, and ***p*-NMe$_2$-^{F}Xyl^{F}Bf**.

compound	1st reduction potential $E_{1/2}$ in [V] vs. Fc/Fc$^+$	2nd reduction potential E_{pc} in [V] vs. Fc/Fc$^+$	1st oxidation potential $E_{1/2}$ in [V] vs. Fc/Fc$^+$
**^{F}Mes^{F}Bf**[a]	–1.13	–2.04	-
**^{F}Xyl^{F}Bf**[a]	–1.21	–2.12	-
***p*-NMe$_2$-^{F}Xyl^{F}Bf**[a]	–1.28	–2.15	0.95 (partially reversible)

Photophysical properties

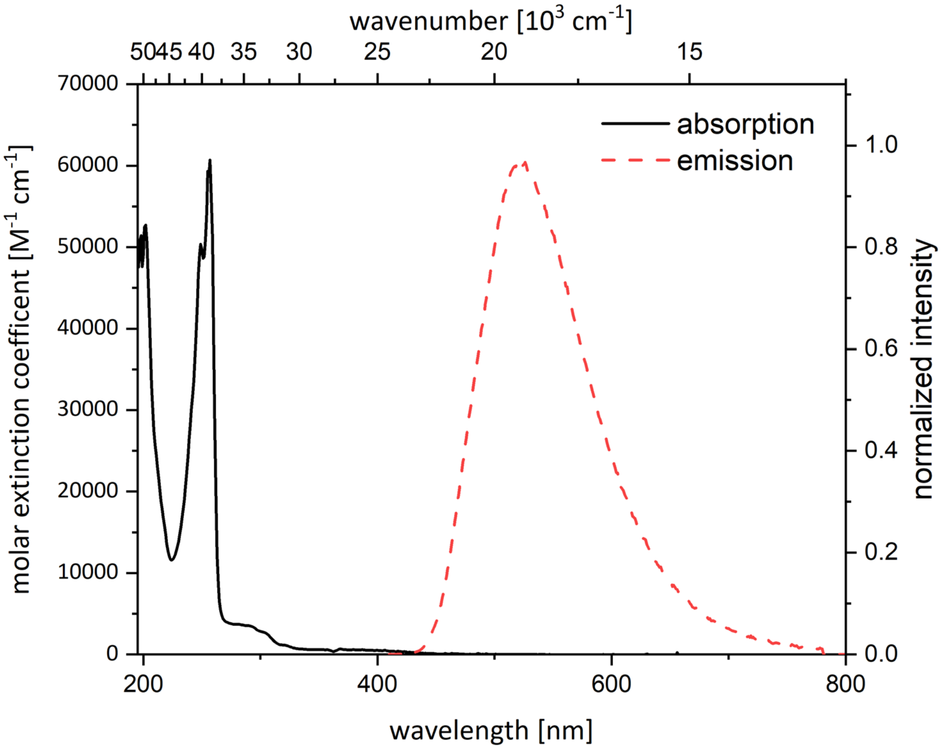

Figure S44: Absorption and emission spectra of **^{F}Mes^{F}Bf** in hexane.

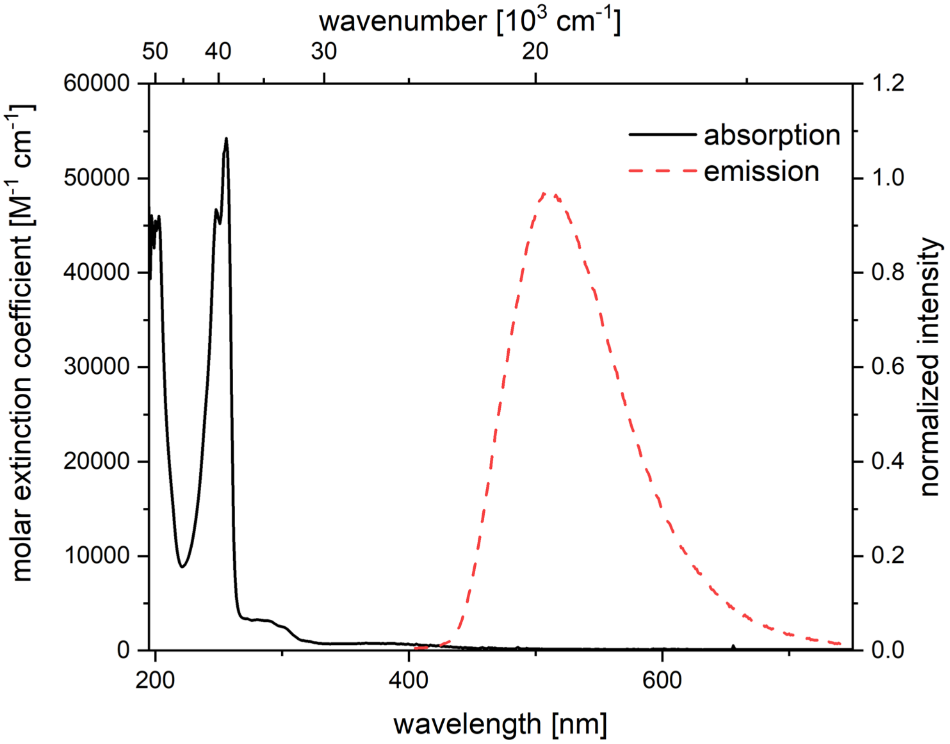

Figure S45: Absorption and emission spectra of **^{F}Xyl^{F}Bf** in hexane.

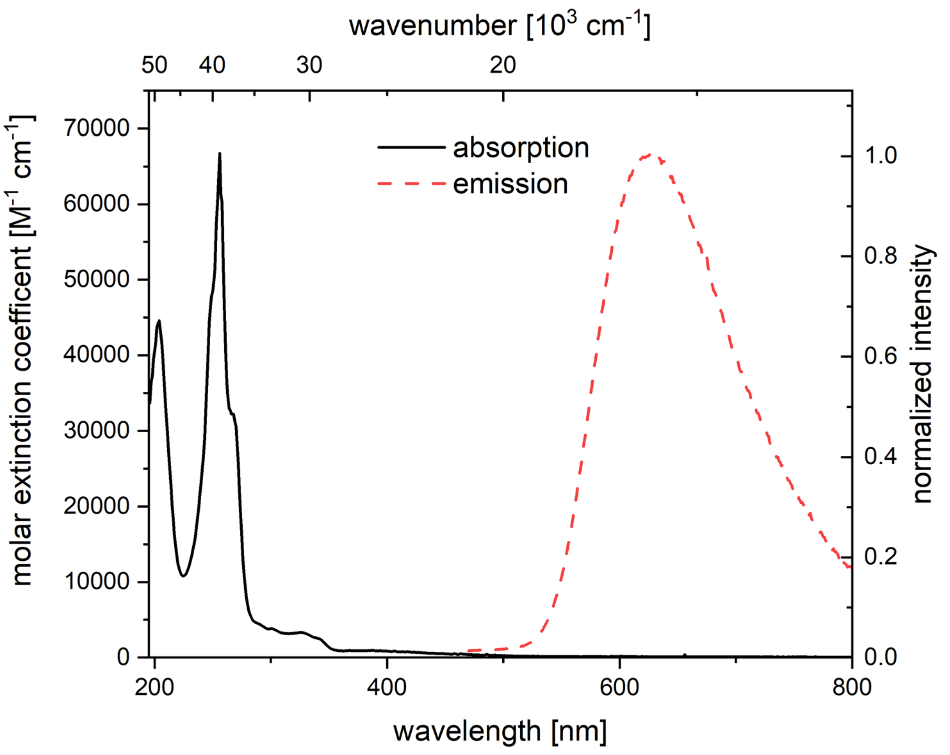

Figure S46: Absorption and emission spectra of ***p*-NMe₂-^{F}Xyl^{F}Bf** in hexane.

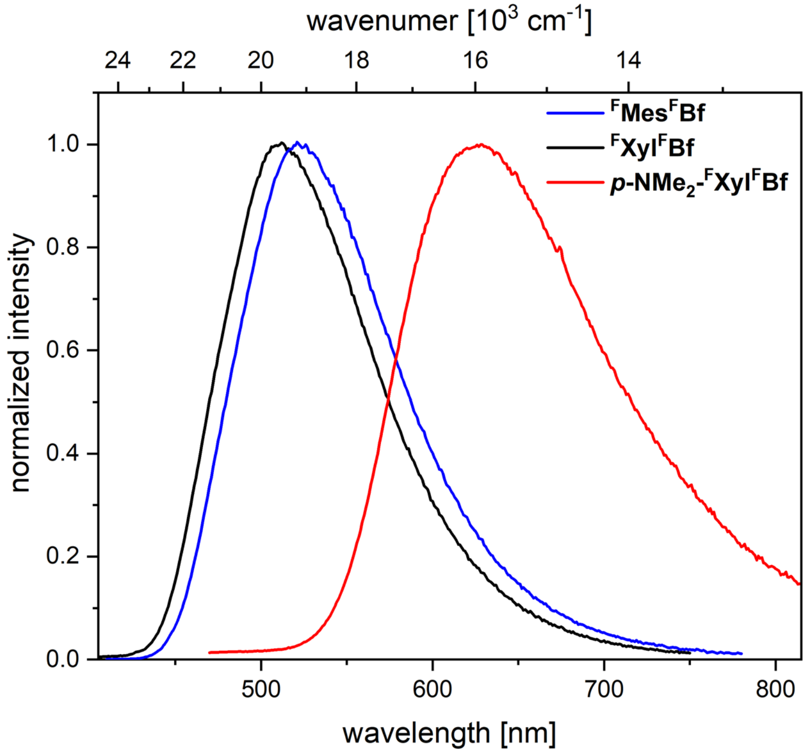

Figure S47: Emission spectra of **^{F}Mes^{F}Bf**, **^{F}Xyl^{F}Bf** and ***p*-NMe$_2$-^{F}Xyl^{F}Bf**.

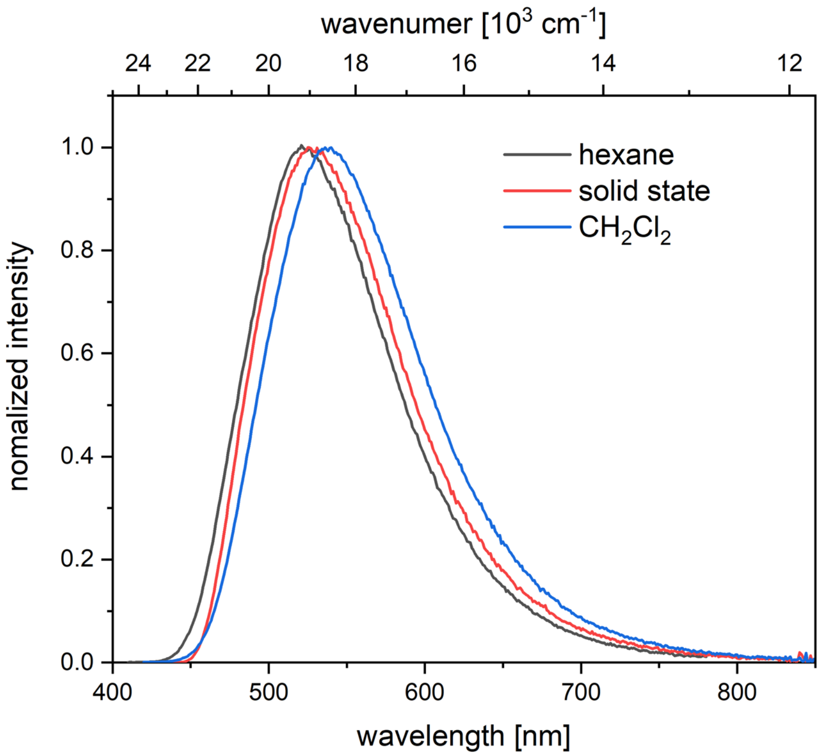

Figure S48: Environment dependent emission spectra of **^{F}Mes^{F}Bf**.

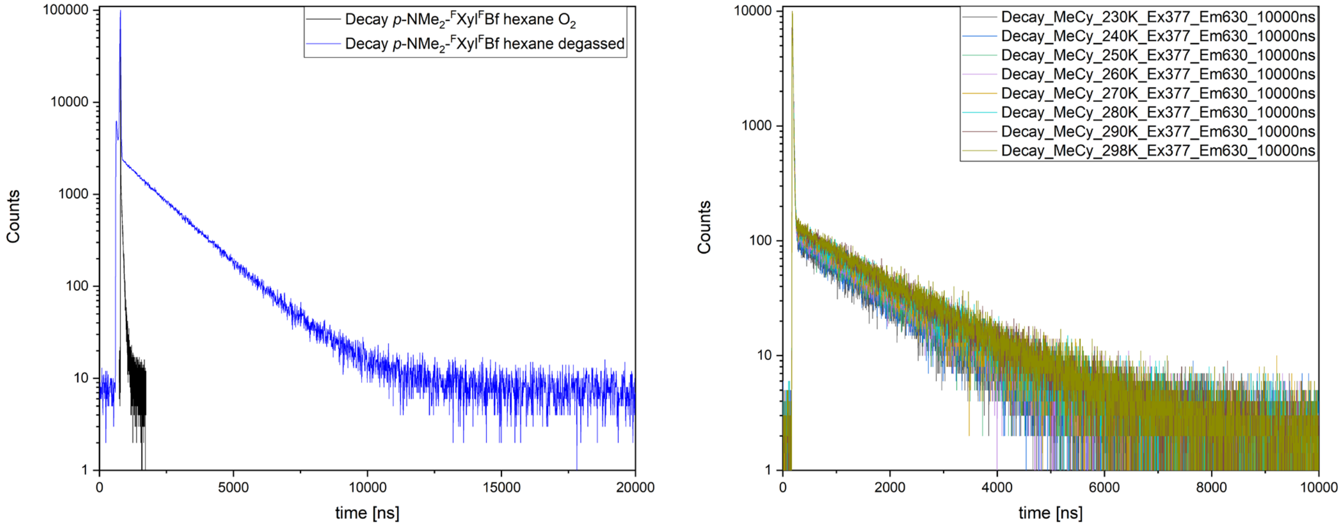

Figure S49: Lifetime measurements of ***p*-NMe$_2$-^{F}Xyl^{F}Bf** in hexane (left) degassed (blue) and after bubbling oxygen through the sample (black). Temperature-dependent lifetime measurements of ***p*-NMe$_2$-^{F}Xyl^{F}Bf** in methylcyclohexane (right) between 298 K and 230 K.

^{F}Mes^{F}Bf•MeCN

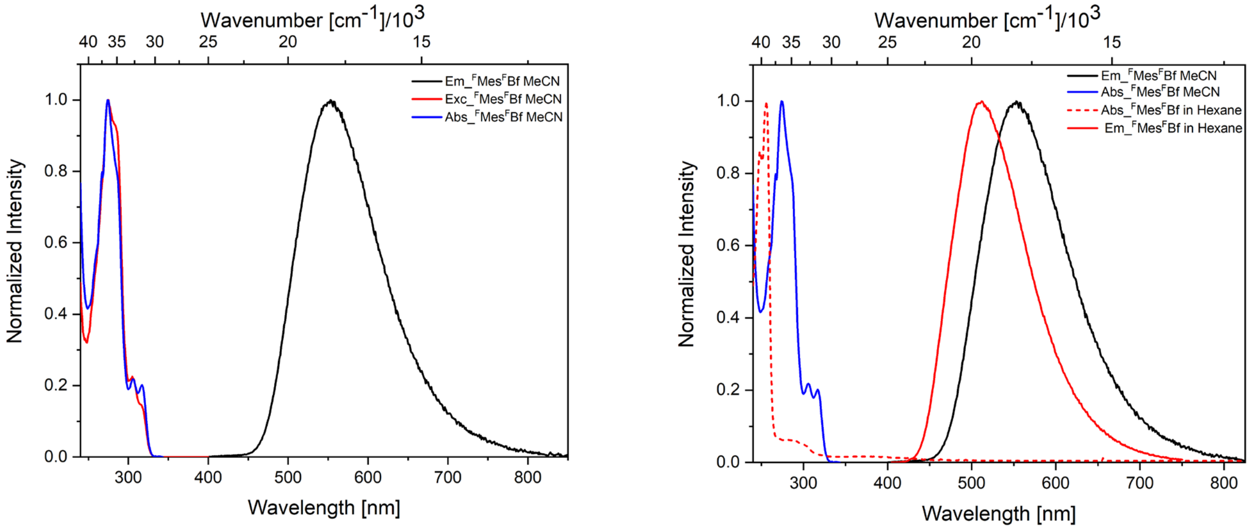

Figure S50: Absorption (blue), excitation (red) and emission (black) spectra of **^{F}Mes^{F}Bf•MeCN** (left). Overlaid absorption spectra of **^{F}Mes^{F}Bf•MeCN** in MeCN (Absorption: blue, Emission: black) and **^{F}Mes^{F}Bf** (Absorption: red dashed, Emission: red) (right)

Table S5: Photophysical data of **^{F}Mes^{F}Bf•MeCN** in MeCN.

Compound	solvent	λ_{abs} [nm]	λ_{em} [nm][a]	apparent Stokes shift (10^3 cm^{-1})	Φ_{fl}	τ [ns]	τ_0 [ns]	k_{nr} [10^7 s^{-1}][b]	k_r [10^7 s^{-1}][c]
^{F}Mes^{F}Bf•MeCN	MeCN	274, 306, 317	553	13.5	0.08	110	1375	0.8	0.07

The acetonitrile adduct **^{F}Mes^{F}Bf•MeCN** shows two weak absorption maxima at λ_{abs} = 317 and 306 nm and a strong absorption maximum at λ_{abs} = 274 nm (Figure S50, Table S5). Compared to **^{F}Mes^{F}Bf** no lower energy absorption could be observed. This is consistent with a population of the empty p_z orbital on the boron center. The emission maximum of **^{F}Mes^{F}Bf•MeCN** at λ_{em} = 553 nm is slightly bathochromically shifted as compared to **^{F}Mes^{F}Bf** (λ_{em} = 521 nm in hexane; λ_{em} = 540 nm in CH_2Cl_2). This is reminiscent of borafluorenes reported by Rupar and co-workers.[19] In their systems the *ortho* methoxymethyl groups of the exo-aryl moieties coordinate to the boron center of the borafluorene, forming a four-coordinate species. However, upon photoexcitation, the boron oxygen bond is cleaved and thus the emission can be attributed to the three-coordinate borafluorene species resulting in a remarkable Stokes shift of up to 16600 cm^{-1}. This is likely similar to what we observe for **^{F}Mes^{F}Bf•MeCN**. Upon photoexcitation, the acetonitrile dissociates and, as such, the resulting emission can be attributed to the three-coordinate borafluorene **^{F}Mes^{F}Bf** leading a Stokes shift of 13500 cm^{-1}. The observed emission lifetime is comparable both to the Rupar's systems (116 – 132 ns) and the lifetimes of the free borafluorenes in the present work (151 – 224 ns). Interestingly, we only observe the excitation spectrum corresponding to **^{F}Mes^{F}Bf•MeCN**, which indicates that adduct formation, after the return to the ground state, must be very fast.

TD-DFT calculations

^{F}Mes^{F}Bf

Calculated absorption spectrum

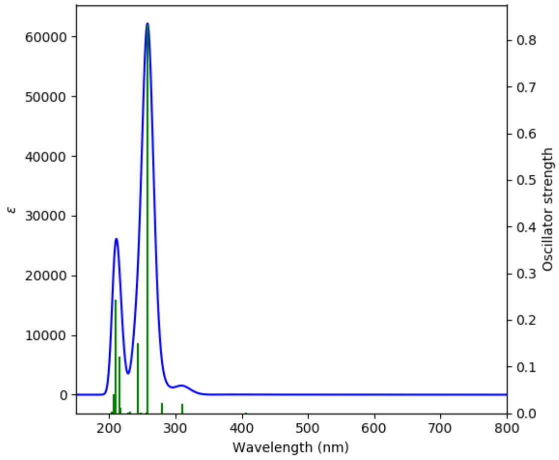

TD-DFT B3LYP/6-31+G(d), gas phase

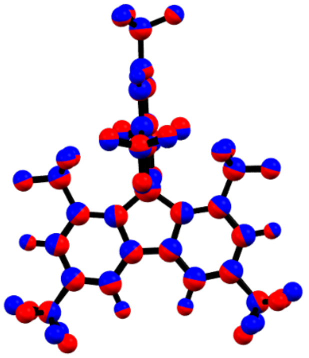

Overlap S_0(blue)-S_1(red)

Orbitals relevant to the $S_1 \leftarrow S_0$ transition

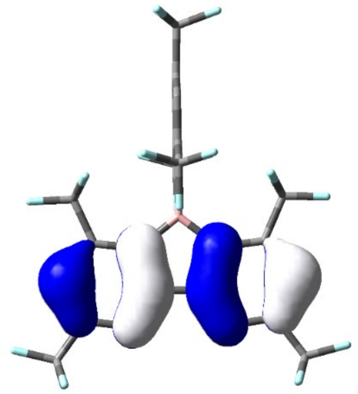

HOMO: –7.65 eV

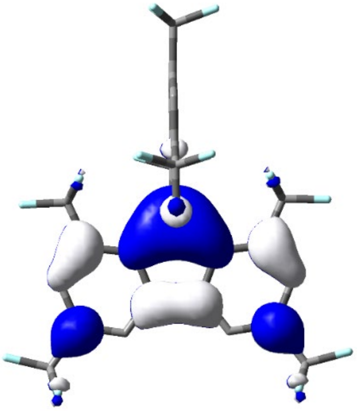

LUMO: –3.80 eV

Table S6: Lowest energy singlet electronic transition of **^{F}Mes**^{F}Bf (TD-DFT B3LYP/6-31+G(d), gas phase).

State	E [eV]	λ [nm]	*f*	Symmetry	Major contributions	Λ
1	3.05	406.32	0.0005	A'	HOMO->LUMO (99%)	0.65
2	3.90	317.96	0	A"	H-1->LUMO (99%)	0.25
3	4.00	309.72	0.0206	A'	H-2->LUMO (73%), HOMO->L+1 (25%)	0.70
4	4.18	296.91	0	A'	H-3->LUMO (97%)	0.26
5	4.44	279.17	0.0217	A'	H-4->LUMO (80%), HOMO->L+4 (12%)	0.77
6	4.80	258.23	0	A"	HOMO->L+3 (100%)	0.10
7	4.80	258.17	0.8333	A'	H-2->LUMO (24%), HOMO->L+1 (70%)	0.73
8	4.85	255.73	0.0005	A'	HOMO->L+2 (99%)	0.07

^{F}Xyl^{F}Bf

Calculated absorption spectrum

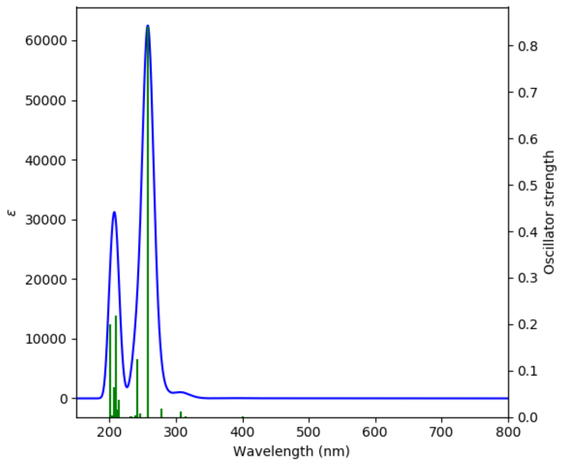

TD-DFT B3LYP/6-31+G(d), gas phase

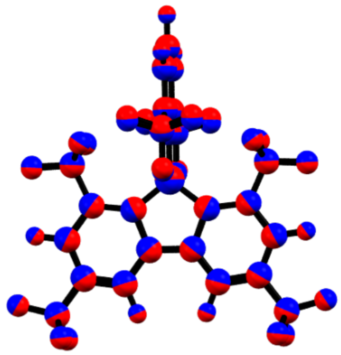

Overlap S_0(blue)-S_1(red)

Orbitals relevant to the $S_1 \leftarrow S_o$ transition

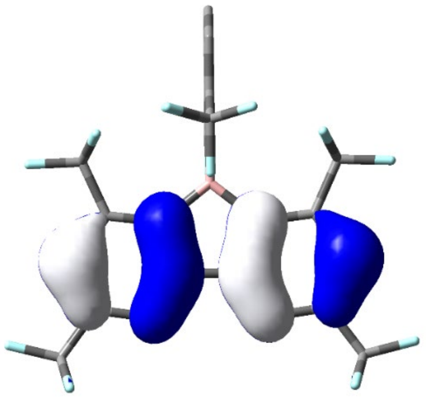

HOMO: –7.49 eV

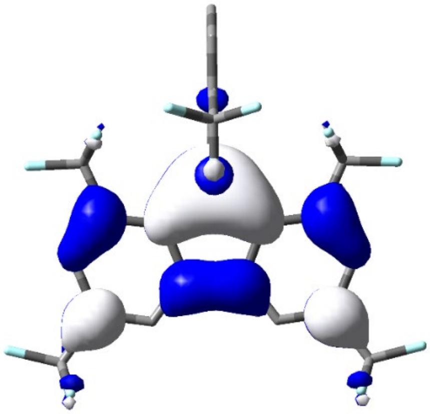

LUMO: –3.60 eV

Table S7: Lowest energy singlet electronic transition of **^{F}Xyl^{F}Bf** (TD-DFT B3LYP/6-31+G(d), gas phase).

State	E [eV]	λ [nm]	*f*	Symmetry	Major contributions	Λ
1	3.09	400.88	0.0006	B_2	HOMO->LUMO (99%)	0.66
2	3.64	340.31	0	A_2	H-1->LUMO (99%)	0.23
3	3.94	314.98	0.0021	B_2	H-2->LUMO (98%)	0.22
4	4.04	306.75	0.0122	B_2	H-3->LUMO (70%), HOMO->L+1 (26%)	0.71
5	4.47	277.65	0.0182	A_1	H-4->LUMO (79%), HOMO->L+3 (13%)	0.77
6	4.81	257.99	0.8413	B_2	H-3->LUMO (28%), HOMO->L+1 (68%)	0.74
7	4.94	250.76	0	A_2	H-1->L+1 (99%)	0.11
8	5.03	246.48	0.0083	A_1	H-5->LUMO (31%), HOMO->L+2 (23%), HOMO->L+3 (36%)	0.60

p-NMe₂-^{F}Xyl^{F}Bf

Calculated absorption spectrum

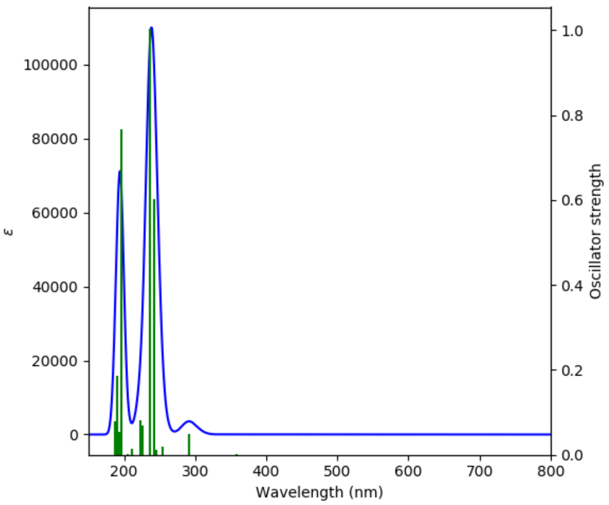

TD-DFT CAM-B3LYP/6-31+G(d), gas phase

Orbitals relevant to the $S_1 \leftarrow S_0$ transition

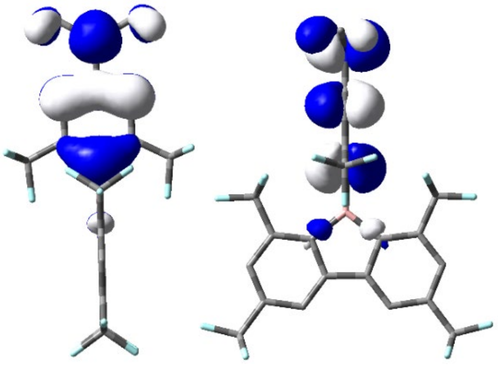

HOMO: –6.17 eV (B3LYP); –7.53 eV (CAM-B3LYP)

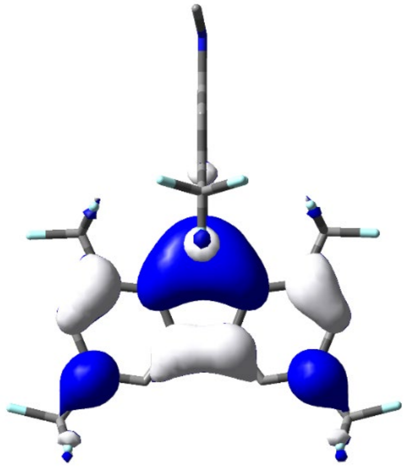

LUMO: –3.41 eV (B3LYP); –2.25 eV (CAM-B3LYP)

Table S8: Lowest energy singlet electronic transition of ***p*-NMe$_2$-^{F}Xyl**^{F}Bf (C_1) (TD-DFT CAM-B3LYP/6-31+G(d), gas phase).

State	E [eV]	λ [nm]	*f*	Symmetry	Major contributions	Λ
1	3.25	382.07	0	A	HOMO->LUMO (92%)	0.15
2	3.46	358.41	0.0006	A	H-1->LUMO (97%)	0.67
3	4.25	291.56	0.0491	A	HOMO->L+2 (70%), HOMO->L+3 (24%)	0.49
4	4.46	277.94	0	A	H-3->LUMO (49%), H-1->L+1 (41%)	0.71
5	4.84	256.21	0	A	HOMO->L+1 (97%)	0.06
6	4.88	254.09	0.0188	A	H-4->LUMO (53%), H-1->L+3 (18%)	0.71
7	5.04	245.93	0.0127	A	H-2->LUMO (95%)	0.20
8	5.11	242.61	0.6022	A	HOMO->L+4 (86%)	0.71

Table S9: Lowest energy singlet electronic transition of ***p*-NMe₂-^FXyl^FBf** (C_2v) (TD-DFT CAM-B3LYP/6-31+G(d), gas phase).

State	E [eV]	λ [nm]	*f*	Symmetry	Major contributions	Λ
1	3.22	385.34	0.0	A_2	HOMO->LUMO (92%)	0.15
2	3.46	358.29	0.0006	B_2	H-1->LUMO (97%)	0.67
3	4.23	292.76	0.0507	B_1	HOMO->L+2 (65%), HOMO->L+3 (29%)	0.48
4	4.46	277.90	0.0	B_2	H-3->LUMO (49%), H-1->L+1 (41%)	0.71
5	4.81	257.74	0.0	A_2	HOMO->L+1 (97%)	0.06
6	4.88	254.06	0.0182	A_1	H-4->LUMO (53%), H-1->L+3 (16%)	0.70
7	5.04	246.06	0.0124	B_2	H-2->LUMO (95%)	0.20
8	5.12	242.09	0.6312	A_1	HOMO->L+4 (86%)	0.72

Theoretical calculations: Cartesian coordinates

^{F}Mes^{F}Bf

DFT B3LYP/6-31G+g(d), gas phase, S_0

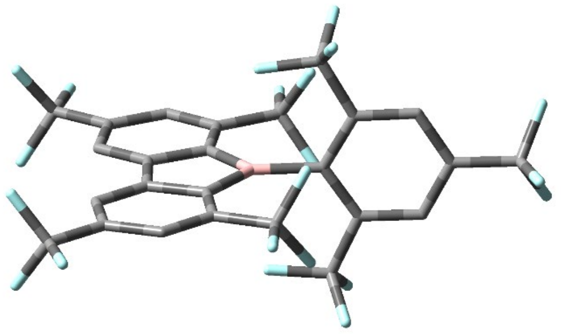

Point group: C_S

Total energy: –1,931,585.71 kcal mol^{-1}

Dipole moment: 0.97 D

Imaginary frequencies: 0

C	1.95932733	-3.48367691	0.00000000
C	3.24125601	-2.94041650	0.00000000
C	3.43542773	-1.55393774	0.00000000
C	2.32085175	-0.72748835	0.00000000
C	1.00315511	-1.24677972	0.00000000
C	0.84227238	-2.63626552	0.00000000
C	2.30925459	0.75377166	0.00000000
C	0.98358821	1.25229130	0.00000000
B	0.01666565	-0.00484050	0.00000000
C	3.41067163	1.59767629	0.00000000
C	3.19464035	2.98093856	0.00000000
C	1.90431100	3.50394216	0.00000000
C	0.80076593	2.63902953	0.00000000
C	-1.55619437	-0.01670955	0.00000000
C	-2.29787139	-0.02348364	-1.19397401
C	-3.69106341	-0.03646791	-1.20427300
C	-4.38994640	-0.04478603	0.00000000
C	-3.69106341	-0.03646791	1.20427300
C	-2.29787139	-0.02348364	1.19397401
C	-1.61001934	-0.01901545	2.53483798
F	-1.92143268	-1.10647906	3.27405300
F	-1.94041199	1.06167897	3.27595697
F	-0.25097833	-0.00690914	2.39855997
C	-1.61001934	-0.01901545	-2.53483798
F	-1.94041199	1.06167897	-3.27595697
F	-1.92143268	-1.10647906	-3.27405300
F	-0.25097833	-0.00690914	-2.39855997
C	-0.58298132	3.24718425	0.00000000
F	-0.55449464	4.59806621	0.00000000
F	-1.29673278	2.86934414	1.08982194
F	-1.29673278	2.86934414	-1.08982194
C	-0.53159540	-3.26635082	0.00000000
F	-0.48167007	-4.61664788	0.00000000
F	-1.25148996	-2.90000294	-1.08963899
F	-1.25148996	-2.90000294	1.08963899
C	4.45670190	-3.83344100	0.00000000
F	4.14009827	-5.14554461	0.00000000
F	5.23547619	-3.60407296	1.08774595
F	5.23547619	-3.60407296	-1.08774595
C	4.39586648	3.89300607	0.00000000
F	4.05864710	5.19995049	0.00000000
F	5.17967020	3.67420009	-1.08635914
F	5.17967020	3.67420009	1.08635914
C	-5.89654636	-0.01031816	0.00000000
F	-6.41511313	-0.61934253	-1.09132996
F	-6.36239378	1.26513242	0.00000000
F	-6.41511313	-0.61934253	1.09132996
H	1.82747000	-4.55756674	0.00000000
H	4.44243523	-1.14842099	0.00000000
H	4.42396015	1.20811204	0.00000000
H	1.75552433	4.57562135	0.00000000
H	-4.22684243	-0.04603226	-2.14749499
H	-4.22684243	-0.04603226	2.14749499

^{F}Mes^{F}Bf

DFT RB3LYP/6-31G+G(d), gas phase, S_1

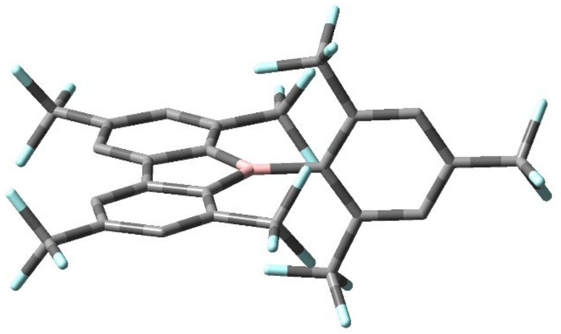

Point group: C_1

Total energy: –1,931,575.25 kcal mol^{-1}

Dipole moment: 2.07 D

C	1.98897900	-3.42531900	-0.00004700
C	3.30662700	-2.88946100	-0.00001300
C	3.50679400	-1.53623400	-0.00004800
C	2.37754800	-0.68840700	-0.00008800
C	1.00893900	-1.20420900	-0.00010300
C	0.87778900	-2.60937300	-0.00009700
C	2.36623700	0.71638900	-0.00008000
C	0.98947100	1.20999200	-0.00009500
B	0.04136900	-0.00484600	-0.00008500
C	3.48158300	1.58244200	-0.00002900
C	3.25942100	2.93221800	0.00002300
C	1.93324100	3.44665500	-0.00000200
C	0.83544600	2.61278600	-0.00007200
C	-1.54028700	-0.01687200	-0.00005700
C	-2.28849700	-0.02340500	-1.18862200
C	-3.67834800	-0.03568500	-1.19650500
C	-4.37600900	-0.04280500	-0.00002600
C	-3.67829400	-0.03572200	1.19646000
C	-2.28847800	-0.02343400	1.18855600
C	-1.66277400	-0.02071200	2.56135800
F	-2.03357500	-1.10776300	3.27007900
F	-2.05976900	1.05264000	3.27693500
F	-0.31634600	-0.00425400	2.55310900
C	-1.66284900	-0.02067200	-2.56144700
F	-2.05998600	1.05261300	-3.27704500
F	-2.03357100	-1.10779200	-3.27011200
F	-0.31642600	-0.00407700	-2.55325600
C	-0.53781600	3.23222100	-0.00011200
F	-0.48771800	4.57737200	-0.00010000
F	-1.24661500	2.86511600	1.08389300
F	-1.24653700	2.86513500	-1.08417800
C	-0.48513900	-3.25114100	-0.00015100
F	-0.41312200	-4.59534100	-0.00019300
F	-1.19986200	-2.89565800	-1.08413700
F	-1.19992200	-2.89573200	1.08381700
C	4.45741300	-3.85192900	0.00010000
F	4.42385200	-4.65596900	1.08187300
F	5.64513700	-3.22383600	-0.00004200
F	4.42378300	-4.65635400	-1.08136200
C	4.39441300	3.91328400	0.00008900
F	4.34774900	4.71690900	-1.08145100
F	5.59219800	3.30453600	0.00004600
F	4.34775100	4.71673000	1.08178500
C	-5.87713900	-0.00962100	0.00018300
F	-6.39330400	-0.61353100	-1.08661800
F	-6.34606000	1.25688100	0.00281700
F	-6.39299700	-0.61785000	1.08475100
H	1.86510800	-4.50239600	-0.00004600
H	4.51126000	-1.12883500	-0.00004500
H	4.49255500	1.19146400	-0.00002600
H	1.79188200	4.52158000	0.00002900
H	-4.21463100	-0.04570900	-2.13920400
H	-4.21456500	-0.04577800	2.13917500

[^{F}Mes^{F}Bf]$^{•-}$

DFT UB3LYP/6-31G+G(d), gas phase, D_1

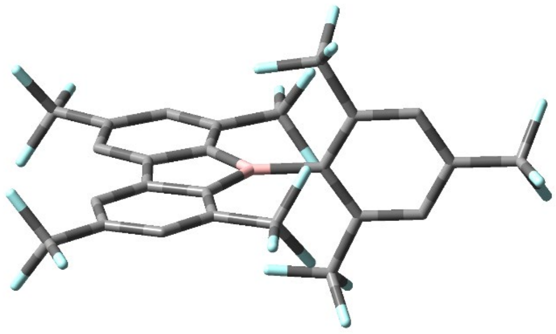

Point group: C_1

Total energy: -1,931,649.96 kcal mol^{-1}

Dipole moment: 2.85 D

C	1.98287600	-3.47945700	-0.00006400
C	3.27855200	-2.94493100	-0.00007700
C	3.46606300	-1.55329700	-0.00009900
C	2.36093000	-0.71920600	-0.00009200
C	1.01343300	-1.22461400	-0.00006300
C	0.87239500	-2.63550900	-0.00004000
C	2.35045900	0.74350900	-0.00009300
C	0.99593300	1.22948800	-0.00006500
B	0.06102900	-0.00430700	-0.00006800
C	3.44344900	1.59350100	-0.00014500
C	3.23579300	2.98225700	-0.00018900
C	1.93253300	3.49803400	-0.00009100
C	0.83440000	2.63809000	-0.00006700
C	-1.52696200	-0.01534100	-0.00003000
C	-2.28557000	-0.02185200	-1.19246400
C	-3.68047200	-0.03485300	-1.19989200
C	-4.38377100	-0.04435700	0.00004800
C	-3.68041300	-0.03467500	1.19994700
C	-2.28550700	-0.02169300	1.19243900
C	-1.67084500	-0.01805700	2.57549700
F	-2.07022500	-1.11037900	3.29134700
F	-2.09570400	1.06182400	3.29594200
F	-0.32922400	-0.00224100	2.60545800
C	-1.67098600	-0.01821500	-2.57555800
F	-2.09544500	1.06196200	-3.29577700
F	-2.07085600	-1.11025100	-3.29158700
F	-0.32936000	-0.00295200	-2.60559600
C	-0.53664900	3.24653000	-0.00002600
F	-0.51905600	4.60901400	-0.00007600
F	-1.27263000	2.88381000	1.08853700
F	-1.27274600	2.88373100	-1.08848300
C	-0.48954900	-3.26396500	-0.00004300
F	-0.45206500	-4.62610800	0.00005000
F	-1.23091500	-2.91218100	-1.08855200
F	-1.23100100	-2.91203400	1.08835400
C	4.47669400	-3.83184700	-0.00022100
F	4.16765500	-5.15476700	0.00068100
F	5.28602200	-3.62618200	1.08522200
F	5.28486800	-3.62743600	-1.08678100
C	4.42100500	3.88641700	0.00036000
F	4.09287900	5.20471400	-0.00354500
F	5.23510300	3.69037900	-1.08325300
F	5.23012800	3.69577100	1.08874200
C	-5.88285900	-0.00903200	0.00003900
F	-6.41577000	-0.61702300	-1.09121300
F	-6.36900300	1.26571700	-0.00067300
F	-6.41577800	-0.61582900	1.09194200
H	1.84701100	-4.55305700	-0.00005000
H	4.47353400	-1.14529700	-0.00011700
H	4.45672900	1.20014300	-0.00019900
H	1.78115000	4.56955800	-0.00011100
H	-4.21558800	-0.04375900	-2.14317200
H	-4.21547800	-0.04344100	2.14325500

^{F}Xyl^{F}Bf

DFT B3LYP/6-31G+g(d), gas phase, S_0

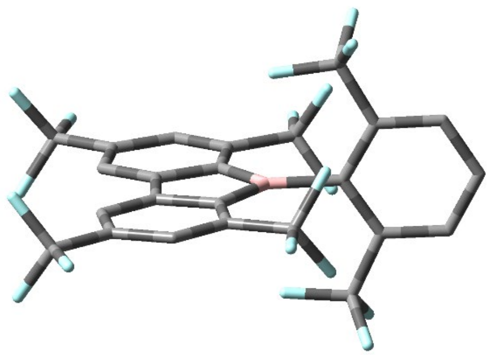

Point group: C_{2v}

Total energy: -1,720,076.00 kcal mol^{-1}

Dipole moment: 2.48 D

Imaginary frequencies: 0

C	0.00000000	2.63823700	-0.18434814
C	0.00000000	3.49330000	-1.29577314
C	0.00000000	2.95938500	-2.58154514
C	0.00000000	1.57439200	-2.78496614
C	0.00000000	0.74055600	-1.67576314
C	0.00000000	1.24974500	-0.35426814
C	0.00000000	-0.74055600	-1.67576314
C	0.00000000	-1.57439200	-2.78496614
C	0.00000000	-2.95938500	-2.58154514
C	0.00000000	-3.49330000	-1.29577314
C	0.00000000	-2.63823700	-0.18434814
C	0.00000000	-1.24974500	-0.35426814
B	0.00000000	-0.00000000	0.62624886
C	0.00000000	-0.00000000	2.19733786
C	1.19388500	-0.00000000	2.93948686
C	1.20145000	-0.00000000	4.33507786
C	0.00000000	-0.00000000	5.03842086
C	-1.20145000	-0.00000000	4.33507786
C	-1.19388500	-0.00000000	2.93948686
C	0.00000000	3.26107000	1.19312486
F	0.00000000	4.61330000	1.14806086
F	1.09010500	2.89416000	1.91070686
F	-1.09010500	2.89416000	1.91070686
C	0.00000000	3.86065100	-3.79025014
F	0.00000000	5.17103300	-3.46508014
F	-1.08822900	3.63824200	-4.57108414
F	1.08822900	3.63824200	-4.57108414
C	0.00000000	-3.26107000	1.19312486
F	0.00000000	-4.61330000	1.14806086
F	-1.09010500	-2.89416000	1.91070686
F	1.09010500	-2.89416000	1.91070686
C	0.00000000	-3.86065100	-3.79025014
F	0.00000000	-5.17103300	-3.46508014
F	1.08822900	-3.63824200	-4.57108414
F	-1.08822900	-3.63824200	-4.57108414
C	2.53088200	-0.00000000	2.24932186
F	3.27628600	-1.08395000	2.56617986
F	3.27628600	1.08395000	2.56617986
F	2.39474200	-0.00000000	0.88863086
C	-2.53088200	-0.00000000	2.24932186
F	-3.27628600	1.08395000	2.56617986
F	-3.27628600	-1.08395000	2.56617986
F	-2.39474200	-0.00000000	0.88863086
H	0.00000000	4.56618600	-1.15640814
H	0.00000000	1.17551900	-3.79465214
H	0.00000000	-1.17551900	-3.79465214
H	0.00000000	-4.56618600	-1.15640814
H	2.14809900	-0.00000000	4.86683786
H	0.00000000	-0.00000000	6.12357786
H	-2.14809900	-0.00000000	4.86683786

^{F}Xyl^{F}Bf

DFT B3LYP/6-31G+g(d), gas phase, S_1

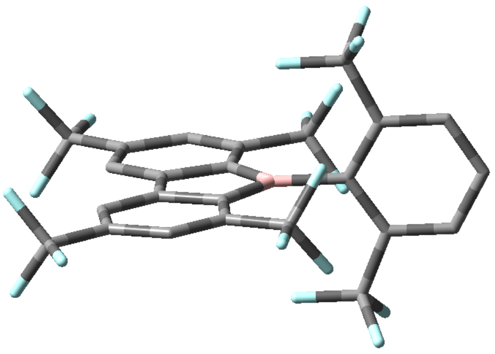

Point group: C_1

Total energy: -1, 720,013.49 kcal mol^{-1}

Dipole moment: 0.96 D

C	0.20467774	2.61737848	0.00033540
C	1.32386459	3.44869365	0.00044778
C	2.64220554	2.92520122	0.00038683
C	2.84500620	1.55834781	0.00020850
C	1.72197197	0.70652135	0.00009486
C	0.34067396	1.21433146	0.00015963
C	1.72196541	-0.70653626	-0.00009671
C	2.84499164	-1.55837328	-0.00021666
C	2.64217811	-2.92522481	-0.00039836
C	1.32383224	-3.44870487	-0.00045653
C	0.20465324	-2.61737913	-0.00033866
C	0.34066271	-1.21433346	-0.00015890
B	-0.62292180	0.00000337	0.00000449
C	-2.20078218	0.00001028	0.00000427
C	-2.95287330	-0.00027246	1.19407634
C	-4.35069064	-0.00027505	1.19818982
C	-5.05591982	0.00002129	0.00000222
C	-4.35068897	0.00031180	-1.19818439
C	-2.95287168	0.00029817	-1.19406867
C	-1.16181618	3.26244211	0.00039648
F	-1.08733526	4.61651342	0.00061558
F	-1.88224418	2.91032784	1.09041761
F	-1.88217549	2.91067944	-1.08978327
C	3.83620723	3.84224770	0.00048028
F	3.48565610	5.14593856	0.00087071
F	4.62238863	3.63340864	-1.08706959
F	4.62266536	3.63282544	1.08771563
C	-1.16184700	-3.26242940	-0.00039837
F	-1.08737951	-4.61650146	-0.00062295
F	-1.88227464	-2.91030361	-1.09041602
F	-1.88219975	-2.91066374	1.08978475
C	3.83617118	-3.84228251	-0.00048840
F	3.48560781	-5.14597004	-0.00096036
F	4.62230818	-3.63350748	1.08710625
F	4.62267763	-3.63281102	-1.08767893
C	-2.32361720	-0.00060340	2.56747692
F	-2.70630354	-1.08684985	3.29016508
F	-2.70648961	1.08517431	3.29077269
F	-0.96763219	-0.00048105	2.56234241
C	-2.32361257	0.00062255	-2.56746776
F	-2.70628861	1.08687124	-3.29015805
F	-2.70649166	-1.08515317	-3.29076301
F	-0.96762756	0.00048954	-2.56232963
H	1.19089832	4.52330627	0.00058170
H	3.85186862	1.15252442	0.00015793
H	3.85185786	-1.15255935	-0.00016988
H	1.19085586	-4.52331624	-0.00059364
H	-4.88369816	-0.00050115	2.14400550
H	-6.14128708	0.00002548	0.00000146
H	-4.88369509	0.00054164	-2.14400086

p-NMe$_2$-^{F}Xyl^{F}Bf

DFT B3LYP/6-31G+g(d), gas phase, S_0

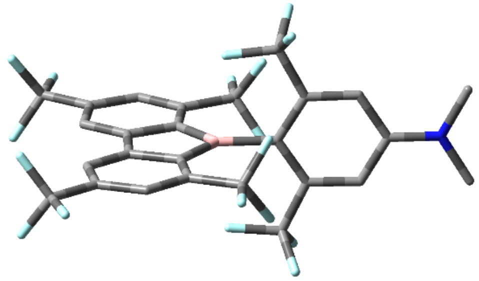

Point group: C_{2v}

Total energy: -1,803,445.62 kcal mol^{-1}

Dipole moment: 5.97 D

Imaginary frequencies: 1

C	1.20112308	0.00000000	-3.81673326
C	1.18370906	0.00000000	-2.42688726
C	0.00000000	0.00000000	-1.67009928
C	-1.18370906	0.00000000	-2.42688726
C	-1.20112308	0.00000000	-3.81673326
C	0.00000000	0.00000000	-4.55742728
N	0.00000000	0.00000000	-5.93371328
C	1.25449813	0.00000000	-6.65511526
C	-1.25449813	0.00000000	-6.65511526
B	0.00000000	0.00000000	-0.10525628
C	0.00000000	-1.25324200	0.86214066
C	0.00000000	-0.76422305	2.19100068
C	0.00000000	0.76422305	2.19100068
C	0.00000000	1.25324200	0.86214066
C	0.00000000	-2.63914799	0.67217861
C	0.00000000	-3.51052704	1.77114557
C	0.00000000	-2.99574209	3.06469259
C	0.00000000	-1.61387010	3.28827165
C	0.00000000	1.61387010	3.28827165
C	0.00000000	2.99574209	3.06469259
C	0.00000000	3.51052704	1.77114557
C	0.00000000	2.63914799	0.67217861
C	-2.52494395	0.00000000	-1.74389632
F	-3.27317297	1.11897607	-2.04530029
F	-2.39677198	0.00000000	-0.38250132
F	-3.27317297	-1.11897607	-2.04530029
C	2.52494395	0.00000000	-1.74389632
F	3.27317297	-1.11897607	-2.04530029
F	2.39677198	0.00000000	-0.38250132
F	3.27317297	1.11897607	-2.04530029
C	0.00000000	-3.24350793	-0.71386842
F	0.00000000	-4.59805993	-0.68439648
F	1.08917609	-2.87129488	-1.42708939
F	-1.08917609	-2.87129488	-1.42708939
C	0.00000000	-3.91447114	4.25948155
F	0.00000000	-5.22044713	3.91572850
F	-1.07885000	-3.69439919	5.05400254
F	1.07885000	-3.69439919	5.05400254
C	0.00000000	3.24350793	-0.71386842
F	0.00000000	4.59805993	-0.68439648
F	-1.08917609	2.87129488	-1.42708939
F	1.08917609	2.87129488	-1.42708939
C	0.00000000	3.91447114	4.25948155
F	0.00000000	5.22044713	3.91572850
F	1.07885000	3.69439919	5.05400254
F	-1.07885000	3.69439919	5.05400254
H	2.15867509	0.00000000	-4.32149625
H	-2.15867509	0.00000000	-4.32149625
H	1.95565911	0.77374634	-6.39097322
H	1.95565911	-0.77374634	-6.39097322
H	1.05865914	0.00000000	-7.72575626
H	-1.95565911	-0.77374634	-6.39097322
H	-1.95565911	0.77374634	-6.39097322
H	-1.05865914	0.00000000	-7.72575626
H	0.00000000	-4.58123803	1.61625252
H	0.00000000	-1.22968214	4.30369067
H	0.00000000	1.22968214	4.30369067
H	0.00000000	4.58123803	1.61625252

***p*-NMe$_2$-^{F}Xyl^{F}Bf**

DFT B3LYP/6-31G+g(d), gas phase, S_0

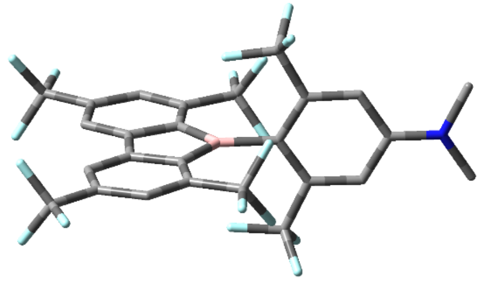

Point group: C_1

Total energy: -1,803,464.96 kcal mol^{-1}

Dipole moment: 5.87 D

Imaginary frequencies: 0

C	-3.84207727	-0.06005421	1.20112311
C	-2.45223116	-0.04258615	1.18370911
C	-1.69544310	-0.03433611	0.00000102
C	-2.45225516	-0.04322715	-1.18368807
C	-3.84210126	-0.06069021	-1.20106707
C	-4.58277132	-0.07935024	0.00004102
N	-5.95905743	-0.11886830	0.00006402
C	-6.68045948	0.02704768	1.25449811
C	-6.68048150	0.02638168	-1.25443308
B	-0.13059998	-0.01059804	-0.00001698
C	0.83679704	1.25324209	-0.00036998
C	2.16565716	0.76422311	-0.00026398
C	2.18757922	-0.71686800	0.00013302
C	0.87377615	-1.24493310	0.00029902
C	0.64683496	2.63914819	-0.00086498
C	1.74580201	3.51052730	-0.00122498
C	3.03934913	2.99574232	-0.00121698
C	3.26292821	1.61387022	-0.00067798
C	3.30941134	-1.53380102	0.00047902
C	3.12662439	-2.92167013	0.00101502
C	1.84880731	-3.47442322	0.00110202
C	0.72467219	-2.63581121	0.00079802
C	-1.76924011	-0.03088712	-2.52494418
F	-2.07064408	-1.11897621	-3.27317323
F	-0.40784500	-0.00987706	-2.39677217
F	-2.10467218	1.04844296	-3.27133123
C	-1.76919311	-0.02938712	2.52494621
F	-2.10441618	1.05056895	3.27053227
F	-0.40779500	-0.00870506	2.39674020
F	-2.07078508	-1.11685321	3.27399227
C	-0.73921217	3.24350818	-0.00113398
F	-0.70974022	4.59806028	-0.00142998
F	-1.45243321	2.87129512	1.08917610
F	-1.45222020	2.87081412	-1.09143207
C	4.23413819	3.91447144	0.00035002
F	3.89038510	5.22044752	-0.01458298
F	5.02865925	3.69439946	-1.07885007
F	5.00996125	3.71463546	1.09709510
C	-0.64320588	-3.28033432	0.00112202
F	-0.57418682	-4.63338941	0.00147102
F	-1.36670295	-2.92890032	-1.08926006
F	-1.36645896	-2.92832032	1.09149610
C	4.34801752	-3.80473714	-0.00034198
F	4.04298455	-5.12028726	0.01254402
F	5.13447057	-3.56268909	1.08008210
F	5.11885357	-3.58061610	-1.09590906
H	-4.34684030	-0.05871523	2.15867518
H	-4.34688230	-0.05986923	-2.15861014
H	-6.41631743	-0.77374637	1.95565917
H	-6.47993152	0.99440077	1.74003915
H	-7.75110058	-0.04388737	1.05865910
H	-6.47997253	0.99348276	-1.74047912
H	-6.41633542	-0.77477437	-1.95518213
H	-7.75112058	-0.04446137	-1.05854507
H	1.59090896	4.58123838	-0.00174498
H	4.27834730	1.22968224	-0.00078798
H	4.31307540	-1.11988694	0.00051202
H	1.72550135	-4.54923831	0.00161002

***p*-NMe$_2$-^{F}Xyl^{F}Bf**

DFT UB3LYP/6-31G+g(d), gas phase, T_1

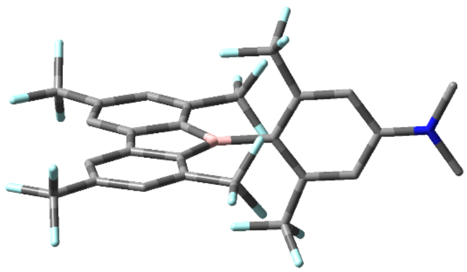

Point group: C_1

Total energy: -1,804,106.70 kcal mol^{-1}

Dipole moment: 7.93 D

C	-3.85183400	0.00044600	1.16148900
C	-2.46360700	0.00025800	1.18328700
C	-1.66794500	0.00003400	0.01575700
C	-2.39973300	0.00003400	-1.19334800
C	-3.78784400	0.00023000	-1.24286200
C	-4.55797900	0.00048000	-0.06060400
N	-5.93086900	0.00081300	-0.09674500
C	-6.69389100	-0.00035400	1.14274100
C	-6.62696900	-0.00076900	-1.37494100
B	-0.09296400	-0.00001300	0.06042100
C	0.86484900	1.21727500	0.03525700
C	2.23700500	0.70966000	0.00983200
C	2.23699800	-0.70969700	0.00990200
C	0.86486400	-1.21731000	0.03537000
C	0.71466300	2.62676400	0.02116300
C	1.81808600	3.46110300	0.01461400
C	3.14536800	2.93038000	0.00183700
C	3.35506900	1.57011000	-0.00106600
C	3.35504700	-1.57014800	-0.00096400
C	3.14529900	-2.93043600	0.00216100
C	1.81810900	-3.46111400	0.01512300
C	0.71464400	-2.62674200	0.02152300
C	-1.73870900	-0.00024500	-2.55573300
F	-2.10993900	-1.08660800	-3.28479500
F	-0.38885000	0.00016500	-2.51759000
F	-2.11056200	1.08542700	-3.28553400
C	-1.86617200	0.00029100	2.57379100
F	-2.26821300	1.08601400	3.28689200
F	-0.51525100	0.00047400	2.58973800
F	-2.26793600	-1.08554900	3.28684300
C	-0.65684400	3.25548500	0.03539400
F	-0.60830900	4.60985000	0.05336300
F	-1.37490000	2.87875800	1.12287300
F	-1.38439600	2.90919000	-1.05635600
C	4.28870600	3.90162700	-0.01110500
F	4.23854700	4.72024500	-1.09592300
F	5.49343900	3.28780000	-0.02901200
F	4.26792400	4.71424200	1.07915000
C	-0.65686200	-3.25544300	0.03595300
F	-0.60835900	-4.60980900	0.05414700
F	-1.38447700	-2.90929500	-1.05579600
F	-1.37484800	-2.87849000	1.12340900
C	4.28862200	-3.90175100	-0.01124300
F	4.26817000	-4.71447000	1.07891400
F	5.49344800	-3.28795700	-0.02946300
F	4.23808700	-4.72022600	-1.09603800
H	-4.38581500	0.00059000	2.10283100
H	-4.27198400	0.00023900	-2.21069200
H	-6.47600100	-0.89199800	1.74518400
H	-6.47529600	0.88982000	1.74703300
H	-7.75784500	0.00039400	0.90623600
H	-6.37650400	0.88929300	-1.96693400
H	-6.37692700	-0.89245400	-1.96472500

p-NMe$_2$-^{F}Xyl^{F}Bf

DFT UCAAM-B3LYP/6-31G+g(d), hexane, T_1

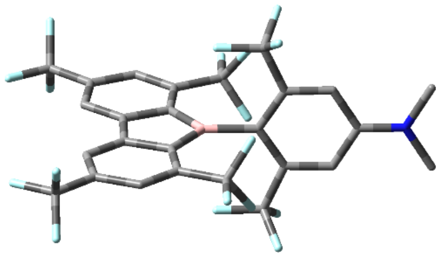

Point group: C_1

Total energy: -1,803,470.87 kcal mol^{-1}

Dipole moment: 23.71 D

C	3.76660480	0.00019287	1.21556178
C	2.39662714	0.00013211	1.20051867
C	1.63182251	0.00027786	0.00004650
C	2.39660591	0.00043269	-1.20043549
C	3.76658341	0.00035773	-1.21550791
C	4.50216839	0.00023631	0.00002019
N	5.84779225	0.00022210	0.00000794
C	6.59688715	0.00067702	1.25848785
C	6.59686525	0.00019520	-1.25848504
B	0.05207328	0.00009301	0.00004629
C	-0.88163949	-1.22264183	-0.00001917
C	-2.22194334	-0.73049371	-0.00001727
C	-2.22215054	0.73004754	0.00000447
C	-0.88198593	1.22258700	-0.00003694
C	-0.71780386	-2.62552282	-0.00007470
C	-1.80812812	-3.47891721	-0.00016893
C	-3.10696883	-2.95724994	-0.00019439
C	-3.31596920	-1.58094883	-0.00009580
C	-3.31642758	1.58017909	0.00004258
C	-3.10783573	2.95653977	-0.00000433
C	-1.80915209	3.47858911	-0.00009574
C	-0.71857023	2.62552155	-0.00012493
C	1.77586238	0.00061326	-2.58656866
F	2.19562579	1.08134415	-3.28352425
F	0.44780659	0.00119007	-2.60906597
F	2.19469918	-1.08051398	-3.28343253
C	1.77592914	-0.00018181	2.58667488
F	2.19510220	-1.08138596	3.28323901
F	0.44787502	-0.00000103	2.60923252
F	2.19541573	1.08046717	3.28390101
C	0.65425168	-3.21591139	-0.00005796
F	0.66389497	-4.56200601	0.00001602
F	1.38594258	-2.83183437	1.08092008
F	1.38591633	-2.83196080	-1.08109738
C	-4.25361858	-3.91243828	-0.00010227
F	-4.23962883	-4.73461009	-1.07922720
F	-5.45150898	-3.29400030	-0.00164696
F	-4.24134400	-4.73239651	1.08075252
C	0.65328726	3.21636904	-0.00025627
F	0.66246667	4.56246465	-0.00047291
F	1.38507575	2.83244382	-1.08122608
F	1.38512638	2.83280131	1.08080279
C	-4.25476899	3.91138835	0.00028238
F	-4.24260073	4.73127808	1.08119027
F	-5.45247589	3.29259552	-0.00115309
F	-4.24115735	4.73363537	-1.07878978
H	4.28426011	0.00016292	2.16536621
H	4.28422147	0.00047958	-2.16532183
H	6.35719621	0.89383472	1.84071995
H	6.35652204	-0.89157892	1.84179714
H	7.66084030	0.00010541	1.03766564
H	6.35634678	-0.89220266	-1.84151602
H	6.35730745	0.89321113	-1.84098739
H	7.66082241	-0.00046239	-1.03768142
H	-1.66067883	-4.55266420	-0.00024177
H	-4.32733155	-1.18856158	-0.00012188
H	-4.32767310	1.18749219	0.00007497
H	-1.66202296	4.55238028	-0.00016451

p-NMe$_2$-^{F}Xyl^{F}Bf

DFT CAM-B3LYP/6-31G+g(d), hexane, S$_1$

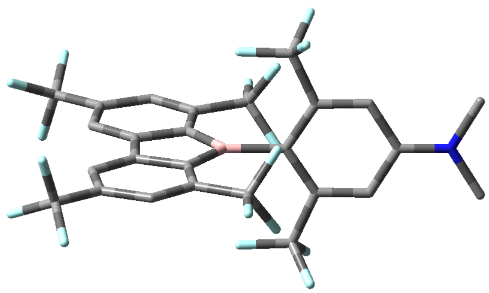

Point group: C$_1$

Total energy: -1,803,461.12 kcal mol^{-1}

Dipole moment: 22.68 D

C	-3.76258331	0.00828866	-1.21833235
C	-2.39865521	0.00535924	-1.20470481
C	-1.62620314	0.00006400	-0.00000155
C	-2.39868428	-0.00518267	1.20468279
C	-3.76261265	-0.00811045	1.21827729
C	-4.50192576	0.00007279	-0.00003645
N	-5.83873920	0.00004455	-0.00005365
C	-6.58872872	0.04193054	-1.25666128
C	-6.58876121	-0.04188720	1.25653325
B	-0.06012645	0.00000820	0.00001422
C	0.87470590	-1.22407905	0.00411113
C	2.21542513	-0.73092709	0.00207032
C	2.21547965	0.73077464	-0.00206223
C	0.87480350	1.22402459	-0.00408349
C	0.70929148	-2.62439039	0.01846658
C	1.80164826	-3.47950857	0.02525225
C	3.09756432	-2.95960366	0.02155367
C	3.30636484	-1.58009134	0.00844141
C	3.30648878	1.57985871	-0.00844113
C	3.09778905	2.95938003	-0.02154289
C	1.80190627	3.47938234	-0.02522423
C	0.70948874	2.62434897	-0.01842849
C	-1.77815773	0.00323085	2.59247046
F	-2.14210175	1.12326838	3.25502837
F	-0.45197829	-0.06403051	2.61627466
F	-2.25087705	-1.03536461	3.31754077
C	-1.77809100	-0.00304040	-2.59247631
F	-2.14187199	-1.12314969	-3.25499829
F	-0.45191964	0.06439486	-2.61624936
F	-2.25092306	1.03546624	-3.31760356
C	-0.66207744	-3.21446030	0.03603450
F	-0.67164694	-4.56006441	0.03833449
F	-1.40699060	-2.83110493	-1.03580848
F	-1.38055924	-2.82842538	1.12577587
C	4.26071865	-3.89320476	-0.02711034
F	4.02570683	-5.04802779	0.63542980
F	5.37930089	-3.35474724	0.50465292
F	4.58549318	-4.25354523	-1.29658862
C	-0.66183945	3.21451721	-0.03597019
F	-0.67131400	4.56012128	-0.03827385
F	-1.40675449	2.83121729	1.03589048
F	-1.38037146	2.82852569	-1.12569375
C	4.26096192	3.89296032	0.02709639
F	4.02629220	5.04738745	-0.63627241
F	5.37977408	3.35415039	-0.50380270
F	4.58512252	4.25409828	1.29650398
H	-4.28298713	0.01077705	-2.16587383
H	-4.28303987	-0.01057644	2.16580621
H	-6.31357439	0.92598258	-1.83606041
H	-6.39592103	-0.85723354	-1.84745006
H	-7.65051494	0.09094412	-1.03138129
H	-6.31357963	-0.92593085	1.83593236
H	-6.39601454	0.85728116	1.84733527
H	-7.65053891	-0.09094908	1.03122353
H	1.65289693	-4.55211963	0.03963291
H	4.31821015	-1.18761225	0.00930261
H	4.31830311	1.18730463	-0.00931611
H	1.65323625	4.55200660	-0.03959564

References

[1] R. Uson, L. A. Oro, J. A. Cabeza, H. E. Bryndza, M. P. Stepro, *Inorg. Synth.*, **1985**, 23, pp. 126-130.

[2] G. R. Fulmer, A. J. M. Miller, N. H. Sherden, H. E. Gottlieb, A. Nudelman, B. M. Stoltz, J. E. Bercaw, K. I. Goldberg, *Organometallics* **2010**, *29*, 2176-2179.

[3] G. M. Sheldrick, *Acta Crystallogr. A* **2015**, *71*, 3-8.

[4] G. Sheldrick, *Acta Crystallogr. A* **2008**, *64*, 112-122.

[5] C. B. Hubschle, G. M. Sheldrick, B. Dittrich, *J. Appl. Crystallogr.* **2011**, *44*, 1281-1284.

[6] Diamond, version 4.6.0; K. Brandenburg & M. Berndt GbR; Bonn (Germany), **2017**.

[7] O. V. Dolomanov, L. J. Bourhis, R. J. Gildea, J. A. K. Howard, H. Puschmann, *J. Appl. Crystallogr.* **2009**, *42*, 339-341.

[8] S. Stoll, A. Schweiger, *J. Magn. Reson.* **2006**, *178*, 42-55.

[9] Gaussian 09, Revision 9.E.01, M. J. Frisch, G. W. Trucks, H. B. Schlegel, G. E. Scuseria, M. A. Robb, J. R. Cheeseman, G. Scalmani, V. Barone, G. A. Petersson, H. Nakatsuji, X. Li, M. Caricato, A. V. Marenich, J. Bloino, B. G. Janesko, R. Gomperts, B. Mennucci, H. P. Hratchian, J. V. Ortiz, A. F. Izmaylov, J. L. Sonnenberg, D. Williams-Young, F. Ding, F. Lipparini, F. Egidi, J. Goings, B. Peng, A. Petrone, T. Henderson, D. Ranasinghe, V. G. Zakrzewski, J. Gao, N. Rega, G. Zheng, W. Liang, M. Hada, M. Ehara, K. Toyota, R. Fukuda, J. Hasegawa, M. Ishida, T. Nakajima, Y. Honda, O. Kitao, H. Nakai, T. Vreven, K. Throssell, J. A. Montgomery, Jr., J. E. Peralta, F. Ogliaro, M. J. Bearpark, J. J. Heyd, E. N. Brothers, K. N. Kudin, V. N. Staroverov, T. A. Keith, R. Kobayashi, J. Normand, K. Raghavachari, A. P. Rendell, J. C. Burant, S. S. Iyengar, J. Tomasi, M. Cossi, J. M. Millam, M. Klene, C. Adamo, R. Cammi, J. W. Ochterski, R. L. Martin, K. Morokuma, O. Farkas, J. B. Foresman, and D. J. Fox, Gaussian, Inc., Wallingford CT, **2016**.

[10] T. Lu, F. Chen, *J. Comput. Chem.* **2012**, *33*, 580-592.

[11] C. Lee, W. Yang, R. G. Parr, *Phys. Rev. B: Condens. Matter Mater. Phys.* **1988**, *37*, 785-789.

[12] G. A. Petersson, M. A. Al-Laham, *J. Chem. Phys.* **1991**, *94*, 6081-6090.

[13] G. A. Petersson, A. Bennett, T. G. Tensfeldt, M. A. Al-Laham, W. A. Shirley, J. Mantzaris, *J. Chem. Phys.* **1988**, *89*, 2193-2218.

[14] S. Grimme, *Chem. Eur. J.* **2004**, *10*, 3423-3429.

[15] T. Yanai, D. P. Tew, N. C. Handy, *Chem. Phys. Lett.* **2004**, *393*, 51-57.

[16] M. J. G. Peach, P. Benfield, T. Helgaker, D. J. Tozer, *J. Chem. Phys.* **2008**, *128*, 044118.

[17] A. Castello-Mico, S. A. Herbert, T. Leon, T. Bein, P. Knochel, *Angew. Chem. Int. Ed.* **2016**, *55*, 401-404.

[18] D. E. Grocock, T. K. Jones, G. Hallas, J. D. Hepworth, *J. Chem. Soc. C Org.* **1971**, 3305-3308.

[19] S. J. Cassidy, I. Brettell-Adams, L. E. McNamara, M. F. Smith, M. Bautista, H. Cao, M. Vasiliu, D. L. Gerlach, F. Qu, N. I. Hammer, D. A. Dixon, P. A. Rupar, *Organometallics* **2018**, *37*, 3732-3741.
